# Short, enantioselective, gram-scale synthesis of (−)-zephyranthine†

**DOI:** 10.1039/d1sc03147c

**Published:** 2021-06-21

**Authors:** Yuxiang Zhao, Yanren Zhu, Guolan Ma, Qi Wei, Shaoxiong Yang, Xiaoyu Zeng, Hongbin Zhang, Jingbo Chen

**Affiliations:** Key Laboratory of Medicinal Chemistry for Natural Resource, Ministry of Education, Yunnan Provincial Center for Research & Development of Natural Products, School of Chemical Science and Technology, Yunnan University Kunming 650091 P. R. China zhanghb@ynu.edu.cn chenjb@ynu.edu.cn

## Abstract

A reasonable synthesis design by strategically integrating functional group manipulation into the ring system construction resulted in a short, enantioselective, gram-scale total synthesis of (−)-zephyranthine. The concise route includes a catalytic Michael/Michael cascade for the asymmetric synthesis of a penta-substituted cyclohexane with three contiguous stereogenic centers, a remarkable 8-step one-pot operation to easily assemble the zephyranthine tetracyclic skeleton, the regioselective construction of a double bond in the C ring and an asymmetric dihydroxylation. This synthesis is also flexible and paves a potential path to a variety of cyclohexylamine-fused tricyclic or polycyclic alkaloids.

## Abstract 2

A reasonable synthesis design by strategically integrating functional group manipulation into the ring system construction resulted in a short, enantioselective, gram-scale total synthesis of (−)-zephyranthine.

## Graphical abstract

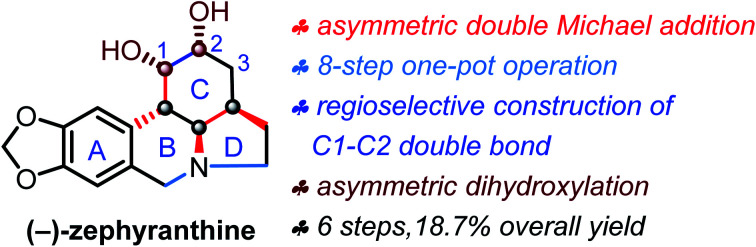

## Introduction

**Fig. 1:**
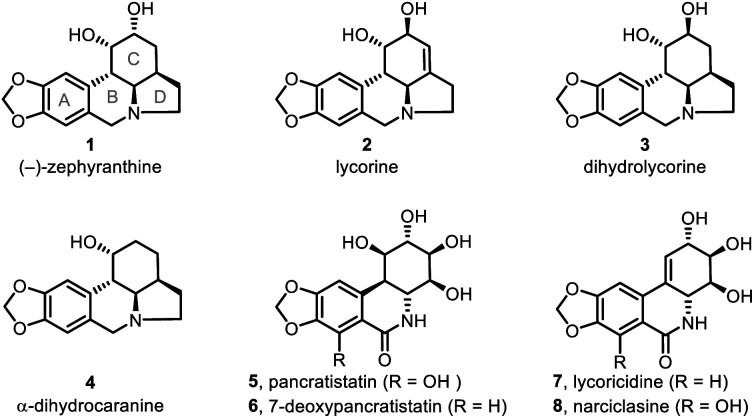
Selected Amaryllidaceae alkaloids.

Lycorine-type alkaloids (*e.g.*, **1–4**; Fig. 1)^1^ are members of an Amaryllidaceae alkaloid sub-class^2^ and display useful biological properties,^3^ including anticholinergic, antiviral, insect antifeedant, and antineoplastic activities, as well as other pharmacological properties.^4^ These alkaloids have attracted substantial synthetic attention because of their tetracyclic core structure, multiple chiral centers and bioactivities. As a result, significant effort has been devoted to assembling the tetracyclic skeleton of such alkaloids^5^ and to the syntheses of the natural products themselves.^6–10^

Unlike other members of the lycorine family, (−)-zephyranthine (**1**)^11^ has only a limited number of syntheses reported for its fabrication,^6*e*,12^ none of which detail a catalytic asymmetric approach. Herein, we report an efficient, enantioselective, gram-scale protocol for **1** that takes advantage of two one-pot reactions. The first is a catalytic asymmetric double Michael addition to construct the C ring with three consecutive chiral centers. The second is a novel 8-step procedure involving double deacetalyzation, nitro group reduction to its corresponding amine, tandem double ring-closing reductive amination, and then double ester hydrolysis with subsequent tandem decarboxylation to give the tetracyclic skeleton of **1**. Although we have successfully developed a remarkably facile route to **1**, we encountered obstacles at a later stage. Unfortunately, the crucial regioselective construction of the C1–C2 double bond in the C ring was hindered by mutable substrates containing nitro groups or amine-type nitrogen atoms. However, this failure was counteracted with the successful, kinetically controlled regioselective enolization of the C ring ketone moiety.

## Results and discussion

The catalytic double Michael addition of γ,δ-unsaturated-β-ketoester and nitroolefin was previously developed by our group^13*d*^ for the asymmetric synthesis of multiple-substituted cyclohexanes bearing 3–5 stereogenic centers, which is expected to develop into the key step of a general method to stereoselectively synthesize a variety of cyclohexylamine-fused alkaloids, including (−)-zephyranthine and lepadiformine-type alkaloids.^14^

**Scheme 1:**
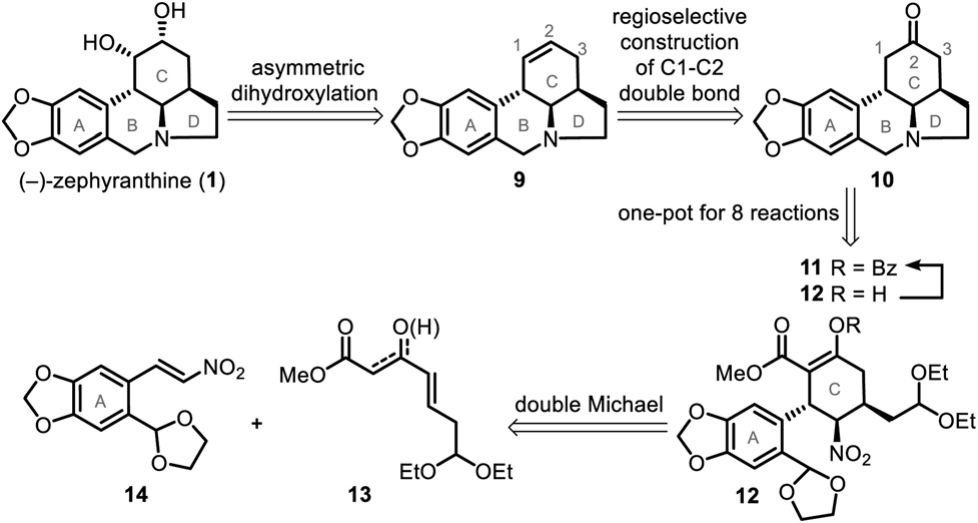
Retrosynthetic analysis of (−)-zephyranthine (**1**).

Our simple retrosynthetic analysis of the target natural product (Scheme 1) revealed that penta-substituted cyclohexane **12**, which arose from a catalytic asymmetric double Michael addition of **13** and **14**, was likely a key intermediate that would result in the direct formation of tetracyclic ketone **10***via* a one-pot operation. Subsequent regioselective construction of a double bond in the C ring, followed by dihydroxylation, would lead to **1**.

**Scheme 2:**
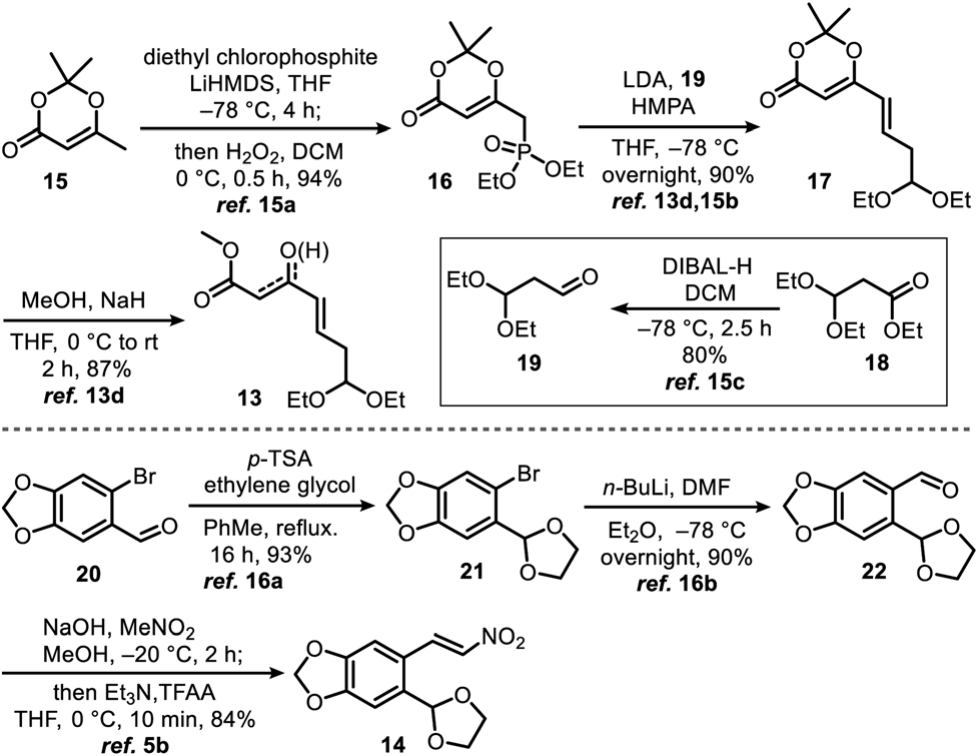
Synthesis of γ,δ-unsaturated-β-ketoester **13** and nitroolefin **14**.

γ,δ-Unsaturated-β-ketoester **13** (ref. 13*d* and 15) and nitroolefin **14** (ref. 5*b* and 16) were prepared on 10 gram scales using a literature method with minor modifications (Scheme 2, see ESI† for detailed preparation methods).

The synthetic journey commenced with a catalytic asymmetric double Michael addition cascade reaction of **13** and **14**, which was promoted by Evans' chiral nickel(ii) catalyst (**23**).^13^ Condition screening (Table 1) revealed that the 1st Michael addition, unlike that in the synthesis of (−)-stenine,^13*d*^ was very sluggish with the low conversion (<10%) even after 10 days' reaction at room temperature with THF as solvent or in solvent-free conditions; DCM as solvent brought about the fastest reaction that afforded 84% yield of the product with inadequate ee (entry 3) in 48 hours. Finally we found that PhMe as the solvent and Triton B as the base gave both the highest enantioselectivity (90%) and diastereoselectivity (>20 : 1), as well as high yield (85%) of penta-substituted cyclohexane **12**, which was identified as the single isomer of an enol ester. Furthermore, NMR analysis showed that three consecutive chiral centers (in the C ring) had been correctly constructed so that **1** would be produced in the subsequent steps.

**Table 1:** Optimization of conditions for asymmetric double Michael addition^a^

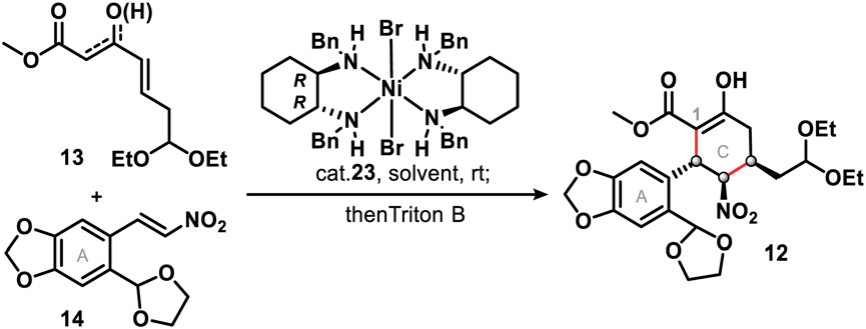						
Entry	**13** : **14**	Solvent	Time (h)	Cat. 23 (mol%)	Yield^b^ (%)	ee^c^ (%)
1	1 : 1	—	240	2	Trace^d^	ND
2	1 : 1	THF	240	2	Trace^d^	ND
3	1 : 1	DCM	48	2	84	76
4	1 : 1	PhMe	96	2	76	90
5	1 : 1	PhMe	96	3	78	90
6	1.1 : 1	PhMe	96	2	81	90
7	**1.2 **:** 1**	**PhMe**	**96**	**2**	**85**	**90**
8	1.3 : 1	PhMe	96	2	85	90

a The reaction was performed in the presence of Triton B as a base (1.0 equiv.) at room temperature.

b Isolated yields after chromatographic purification.

c Enantiomeric excess was determined by high performance liquid chromatography (HPLC), chiracel columns.

d Reaction was very sluggish with the low conversion after 10 days.

**Scheme 3:**
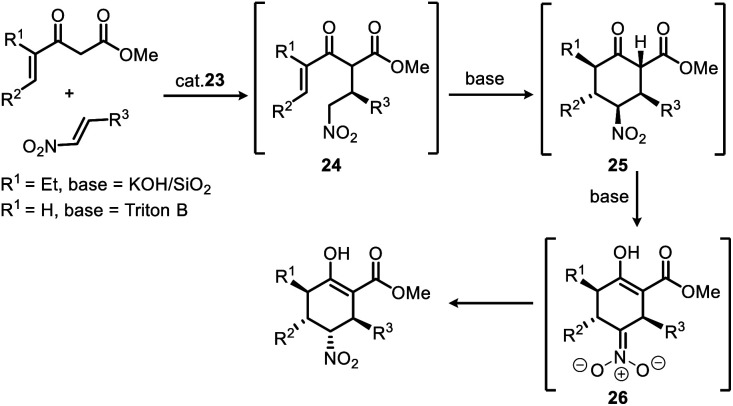
Stereoselective synthesis of multiple-substituted cyclohexanes *via* a Michael/Michael/isomerization cascade reaction.

It can be speculated, as shown in Scheme 3, the steric and electronic effects caused by the γ-ethyl group of the product (R^1^ = Et, for enantioselective synthesis of stenine)^13*d*^ of 1st Michael addition make the intermediate **24** (R^1^ = Et) a less active Michael acceptor in the 2nd Michael addition, therefore, a heterogeneous strong base (KOH/SiO_2_) condition was required to promote this reaction while avoiding damage to the nitro group. Moreover, an isomerisation phenomenon was observed after the 2nd Michael addition that the keto ester intermediate **25** gradually transformed into its enol ester isomer accompanied by inversion of configuration at the N_α_-carbon. Evidently, γ,δ-unsaturated-β-ketoester (R^1^ = H) in this work is more active, and the 2nd Michael addition as well as the subsequent isomerisation progressed rapidly and completed in 20 minutes after addition of Triton B to the reaction mixture upon completion of 1st Michael addition.

**Scheme 4:**
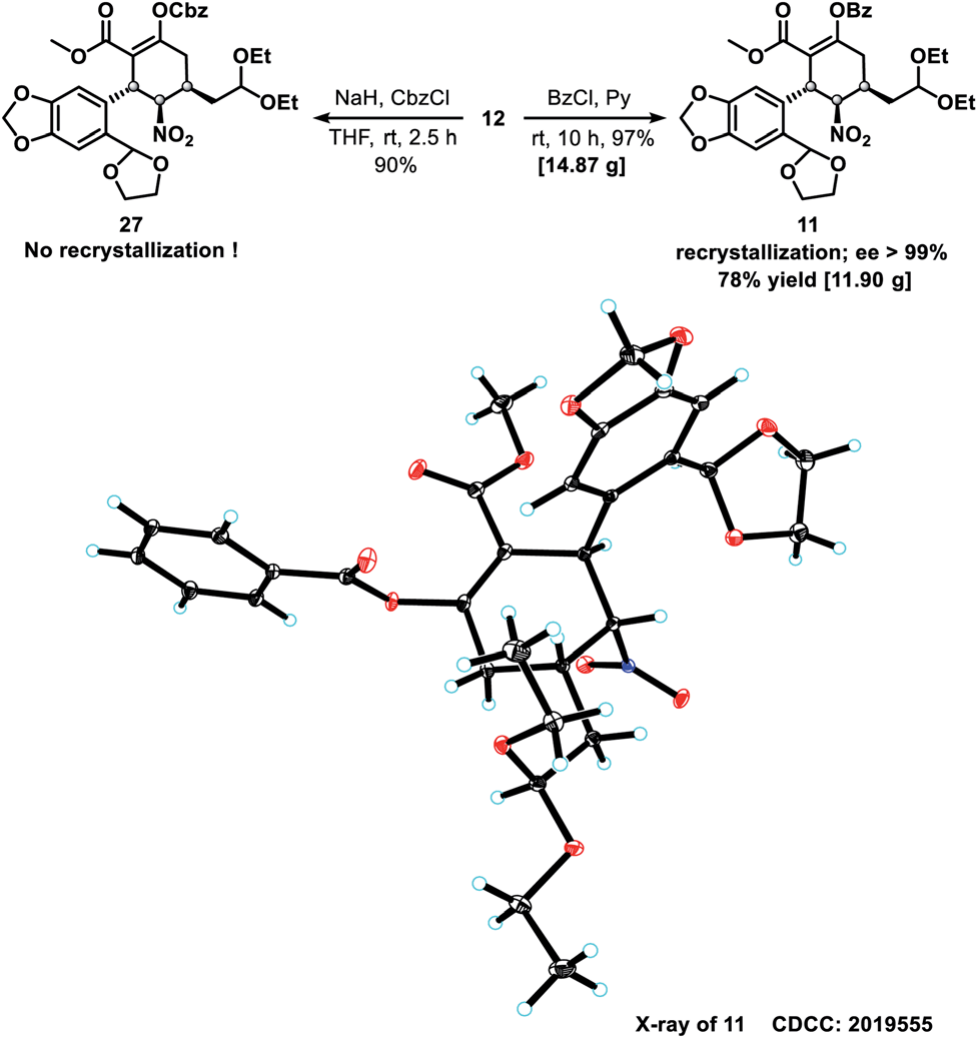
Protection of the enol hydroxy group of **12** and the absolute configuration of compound **11**.

To confirm the absolute configuration of compound **12**, its enol moiety was either benzyloxycarbonyl (Cbz)- or benzoyl (Bz)-protected to prevent unwanted aldol reactions between the α-carbon (C1) of the β-keto ester and the aldehydes that would form from the acetal moieties upon their subsequent deprotection. Cbz-protection was achieved by treating **12** with benzyl carbonochloridate (CbzCl) in the presence of NaH to give ester **27** in 90% yield. However, **27** was difficult to purify by recrystallization. By replacing CbzCl with benzoyl chloride (BzCl), similar esterification of **12** afforded benzoate **11** in quantitative yield. After recrystallization, the isomeric purity of **11** was greater than 99% ee, as determined by high performance liquid chromatography (HPLC). The absolute configuration of benzoate **11** was confirmed by X-ray crystallography (Scheme 4) with Cu-K_α_ radiation.

**Scheme 5:**
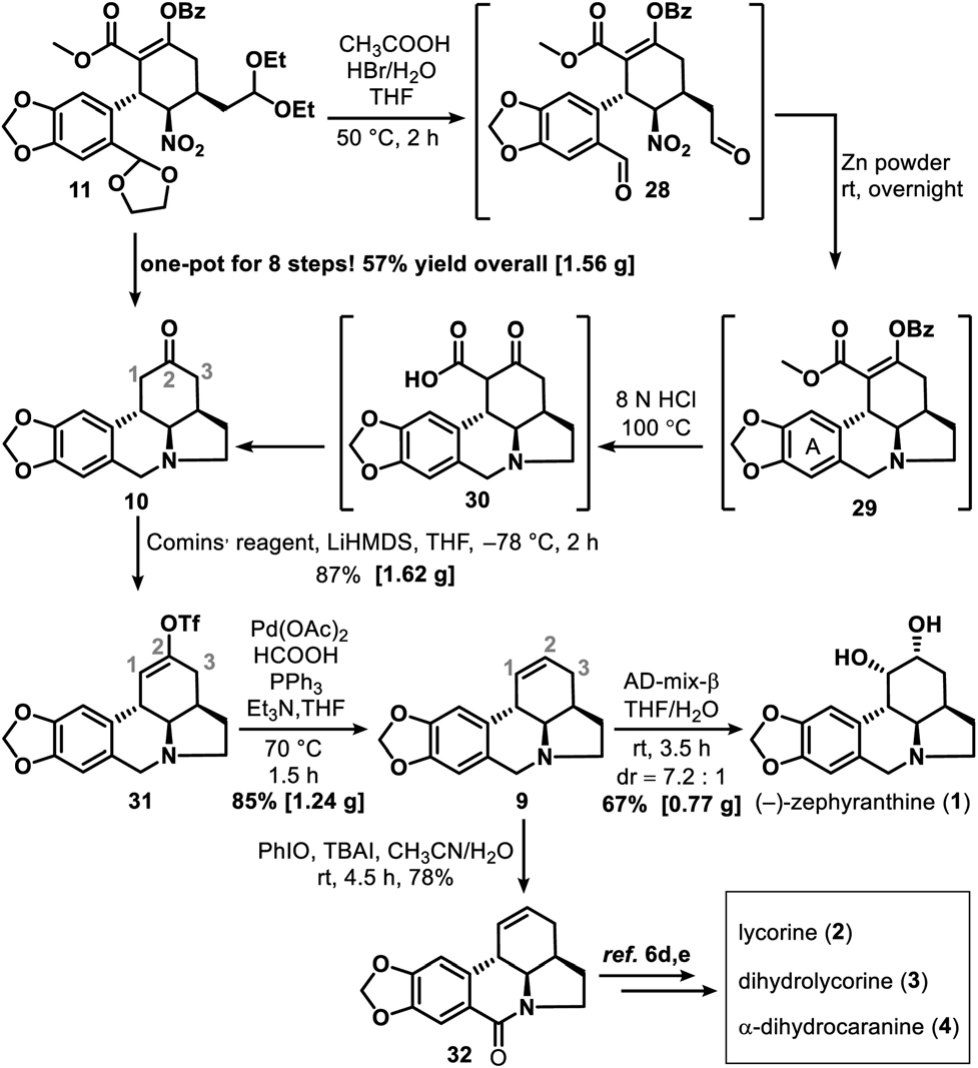
Total synthesis of (−)-zephyranthine (**1**) and synthesis of **32**.

Successful construction of the three contiguous stereogenic centers in the newly formed cyclohexane ring allowed us to begin synthesizing **1**. Cyclization of **11** to form tetracyclic ketone **10** was accomplished through a multistep one-pot operation (Scheme 5), which began by treating **11** with HBr (1.0 equiv., 33% in HOAc) in HOAc–THF–H_2_O (5 : 1 : 1) at 50 °C for 2 h to give dialdehyde **28**. Subsequent reaction of **28** with zinc powder at room temperature overnight gave **29**. After a simple filtration to remove the excess zinc and other solid substances, HCl (8.0 N, 100 equiv.) was added to the reaction mixture to hydrolyze **29** into **30**. Tandem decarboxylation of **30** then delivered key intermediate **10** in a total yield of 57% *via* an eight-step one-pot synthesis.

The reaction of ketone **10** with lithium bis(trimethylsilyl)amide and Comins' reagent^17^ at −78 °C was a kinetically controlled regioselective enolization, which was followed by triflation to afford enol triflate **31** in 87% yield. This then underwent a palladium-promoted hydrogenolysis^18^ to give **9** in 85% yield. In the last step, an attempt to avoid oxidative damage of the amino nitrogen atom was made by adding some acid to the reaction system; however, this failed owing to the deactivation of AD-mix-β under acidic conditions. Fortunately, the most conventional sharpless asymmetric dihydroxylation^19^ of **9** with AD-mix-β under acid-free conditions proceeded smoothly and gave **1** in 67% isolated yield (76% yield of **1** and its diastereoisomer in a ratio of 7.2 : 1). After that, amide **32** was synthesized in 78% yield *via* a PhIO promoted oxidation^20^ of **9**. Our approach thus provided a formal synthesis of a number of other lycorine-type alkaloids^12*a*^ (Scheme 5), such as lycorine (**2**),^6*d*,*e*^ dihydrolycorine (**3**)^6*d*,*e*^ and α-dihydrocaranine (**4**).^6*d*,*e*^

**Scheme 6:**
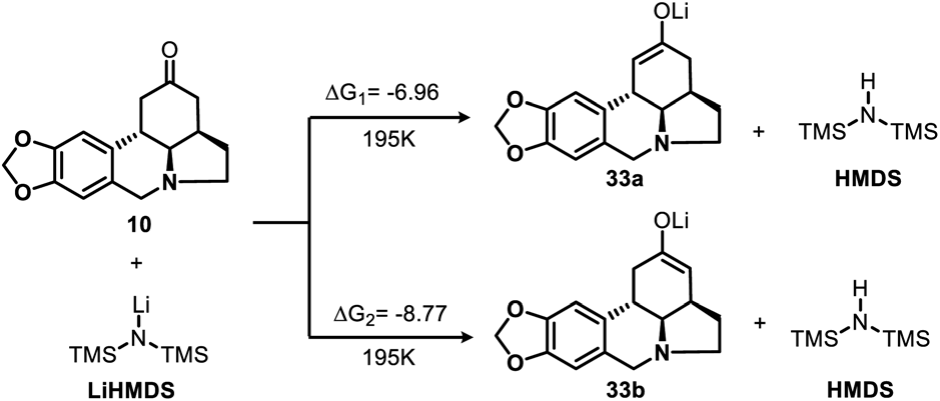
DFT calculations for enolization reaction of **10** (kcal mol^−1^).

To gain additional insight into the nature of the regioselective enolization of ketone **10**, we conducted a theoretical study and the DFT quantum-chemical calculations (Scheme 6, see ESI† for details) revealed that the formation of intermediate **33a** is kinetically favored over that of **33b**.

**Scheme 7:**
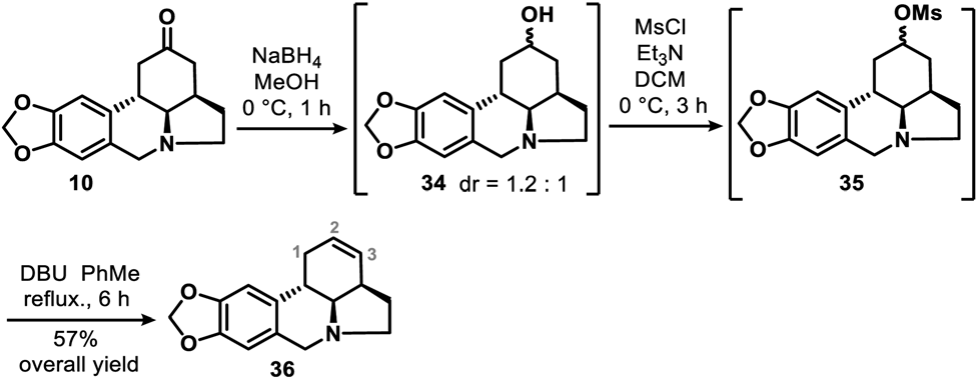
Synthesis of the double bond positional isomer (**36**) of **9**.

We also obtained **36**, the double bond positional isomer of **9**, from the same intermediate **10** that gave **9**. This was accomplished through a 3-step chemical manipulation of the ketone moiety of the C ring (Scheme 7). Intermediate **10** was reduced with sodium borohydride in methanol to give secondary alcohol **34** (dr = 1.2 : 1), which underwent mesylation and then DBU-promoted, thermodynamically controlled methanesulfonic acid elimination to afford **36** as a single regioisomer in 57% overall yield.

**Scheme 8:**
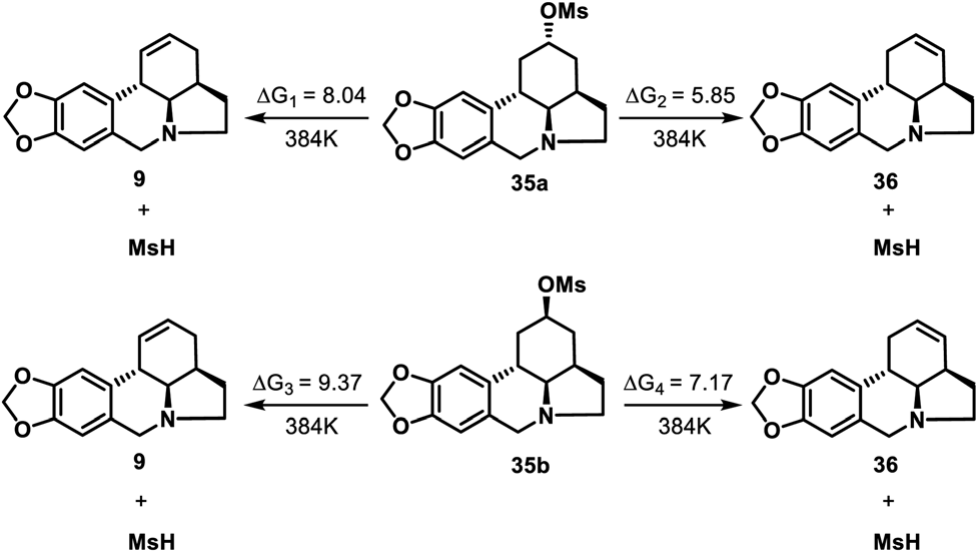
DFT calculations for elimination reaction of **35** (kcal mol^−1^).

To confirm the proposed thermodynamically controlled process, we conducted DFT calculations (see ESI† for details) of elimination reactions of mesylate **35** as indicated in Scheme 8. For both **35a** and **35b**, the formation of olefin **36** is more favorable than formation of **9** according to the free energy changes. **35a** is more likely to undergo elimination than **35b** to form compound **36** as less energy required. The calculation results supported our conclusion that the formation of **36** by elimination reaction of **35** (both **35a** and **35b**) is a thermodynamically controlled process.

**Scheme 9:**
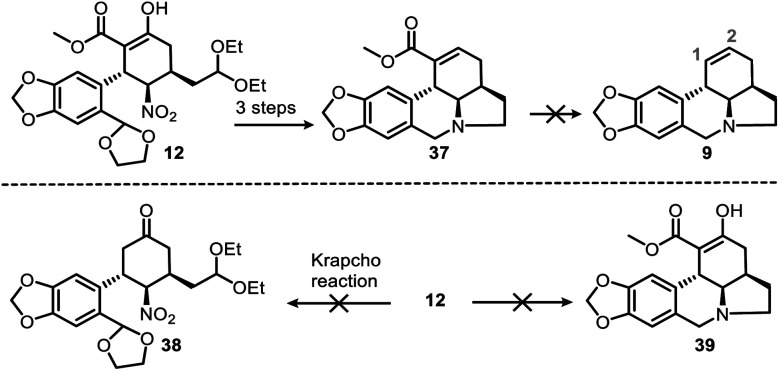
Failed alternative routes to produce (top) the C-ring double bond and (bottom) compounds **38** and **39** from **12**.

We had also attempted other routes to regioselectively construct the double bond in the C ring, but these did not proceed as we expected (see ESI† for detailed informations). Ideally, deesterification of **37** could efficiently provide **9** (Scheme 9, top) and ensure that the double bond remained in the correct position (C1–C2); however, this reaction was unsuccessful. We did manage to convert the ester group of **37** into a carboxyl or aldehyde group, but the subsequent decarboxylation or deformylation failed. In addition, transformation of **12** to **38** (Scheme 9, bottom) could not be achieved through direct deesterification, and attempts to obtain **39** from **12** with the same one-pot protocol that gave **10** from **11** (Scheme 5) were also unsuccessful due to unwanted aldol reactions.

## Conclusions

The natural product (−)-zephyranthine (**1**) was synthesized using a highly efficient and practical approach. Strategically integrating functional group manipulation into the ring system construction resulted in two, multi-step, one-pot reactions that greatly simplified the overall operation and improved its efficiency. From readily available **13** and **14**, only six steps (18.7% overall isolated yield) were necessary to acquire 1 g of (−)-**1**. In addition, regioselective construction of the C ring double bond from **10** delivered **9** or **36** through kinetically or thermodynamically controlled pathways, respectively. This, together with the concise synthesis of amide **32**, provided a flexible and practical synthetic pathway for lycorine-type alkaloids and their analogs. The development of multistep one-pot reactions with greater efficiency and further applications in lepadiformine-type alkaloid syntheses are currently underway.

## Data availability

All computational data associated with this article have been inserted in ESI.

## Author contributions

H. Z. and J. C. conceived the idea. Y. Z. conducted the most of experiments. Y. Z., G. M., Q. W., S. Y. and X. Z co-synthesized part of substrates. H. Z. and J. C. co-wrote the paper. All the authors discussed the results and commented on the manuscript.

## Conflicts of interest

There are no conflicts to declare.

## Supplementary Material

SC-012-D1SC03147C-s001

SC-012-D1SC03147C-s002
