# Supplementary material for: Short, enantioselective, gram-scale synthesis of (−)-zephyranthine
Source: Chem Sci. 2021 Jun 21;12(27):9452–7. doi: 10.1039/d1sc03147c (PMC8278928; doi:10.1039/d1sc03147c)
Supplement: SC-012-D1SC03147C-s001 [file SC-012-D1SC03147C-s001.pdf]

Electronic Supplementary Material (ESI) for Chemical Science.
This journal is © The Royal Society of Chemistry 2021

Supporting Information

Short, Enantioselective, Gram-scale Synthesis of (−)-zephyranthine

Yuxiang Zhao, Yanren Zhu, Guolan Ma, Qi Wei, Shaoxiong Yang, Xiaoyu Zeng, Hongbin Zhang,* and Jingbo Chen*

Key Laboratory of Medicinal Chemistry for Natural Resource, Ministry of Education; Yunnan Research & Development Center for Natural Products; School of Chemical Science and Technology, Yunnan University,
Kunming, 650091, P. R. China
E-mail: zhanghb@ynu.edu.cn; chenjb@ynu.edu.cn

Table of Contents

1. General information.....2

2. Experimental section.....3

3. Crude ^{1}H NMR for AD-mix-β mediated asymmetric Dihydroxylation.....22

4. Crude ^{1}H NMR of 34.....22

5. Computational details.....23

6. References.....34

7. ^{1}H and ^{13}C NMR spectra for all compounds.....35

9. Determination of the Enantiomeric Excess by HPLC Analysis.....62

10. X-Ray Crystallographic Data for 11.....64

1. General information

Proton nuclear magnetic resonance (^{1}H-NMR) spectra were measured on Bruker Avance 400 spectrometers at 400 MHz. Carbon-13 nuclear magnetic resonance (^{13}C-NMR) spectra were recorded on Bruker Avance 400 spectrometers at 100 MHz. Chemical shifts (δ scale) are reported in parts per million (ppm). Data are reported as follows: chemical shifts (ppm), multiplicity (s = singlet, d = doublet, t = triplet, q = quartet, br = broad, m = multiplet), coupling constants (Hz), and integration. Melting points were determined on a XT-4 melting point apparatus. Optical rotations were recorded on a JASCO P-2000 polarimeter. High-resolution mass spectra (HRMS) were acquired using Varian 7.0T FTMS or Agilent 6520 Q-TOF LC/MS with electrospray ionization (ESI) source. Chiral HPLC analyses were performed on Agilent 1100 series with a tunable UV detector at wavelength λ = 250 nm. Column chromatography was performed on silica gel (200–300 mesh) and silica GF254 for TLC were produced by Merch Chemicals Co. Ltd. (Shanghai). Toluene and THF were freshly distilled from sodium/benzophenone prior to use under an argon atmosphere. Reagents used in reactions were obtained commercially from Acros, Aldrich, Adamas-beta®, and were used without purification, unless otherwise indicated. All moisture-sensitive reactions were conducted in oven-dried glassware under a positive pressure of dry nitrogen or argon. Reagents and starting materials were accordingly transferred via syringe or cannula. Unless otherwise stated, all other reactions were performed under a positive nitrogen atmosphere. Reaction temperatures refer to the external oil bath temperature.

2. Experimental section

Preparation of diethyl ((2,2-dimethyl-4-oxo-4*H*-1,3-dioxin-6-yl)methyl) phosphonate (16)[1a]

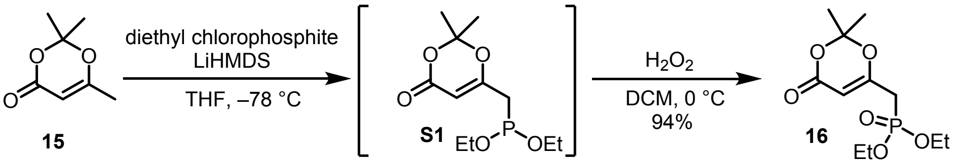

To a stirred solution of **15** (12.4 g, 87.2 mmol) in dry THF (200 mL) at −78 ºC under N_2 atmosphere was slowly added LiHMDS (87.2 mL, 1 M in THF, 87.2 mmol) via syringe over a period of 60 min. After stirring at the same temperature for another 3 h, diethyl chlorophosphite (10.5 g, 67.1 mmol) in THF (50 mL) was slowly added via syringe over a period of 30 min and the resulting mixture was stirred for another 30 min before warming up to room temperature. After stirring at room temperature for 1 h, the mixture was treated with aq. satd. NH_4Cl (120 mL). Then the organic layer was separated and the aqueous layer was extracted with dichloromethane (3 × 100 mL), the organic extracts were combined and washed with brine (100 mL), dried over Na_2SO_4, and concentrated under vacuum to get product **S1** as a yellow oil. After that, the residue (**S1**) was dissolved in dichloromethane (200 mL) at 0 ºC, H_2O_2 (30 w %, 20.8 mL, 201 mmol) was slowly added over a period of 30 min and the reaction mixture kept stirring for another 30 min, the organic layer was separated and the aqueous layer was extracted with dichloromethane (3 × 100 mL). The combined organic phase was washed with brine (100 mL), dried over Na_2SO_4, filtered and concentrated under reduced pressure. The residue was purified by silica gel column chromatography (Petroleum ether : Ethyl acetate = 10 : 1 to Petroleum ether : Ethyl acetate : Triethylamine = 100 : 50 : 0.15) to afford the starting material **15** (2.40 g) and the product **16** (14.2 g, 76%, brsm 94%) as a yellow oil.

Rf = 0.25 (silica, Ethyl acetate). ^{1}H NMR (400 MHz, $CDCl_3$) δ 5.38 (d, *J* = 3.6 Hz, 1H), 4.18 – 4.11 (m, 4H), 2.79 (d, *J* = 22.1 Hz, 2H), 1.71 (s, 6H), 1.34 (t, *J* = 7.1 Hz, 6H). ^{13}C NMR (100 MHz, $CDCl_3$) δ 163.1, 163.0, 160.49, 160.46, 107.1, 96.1, 96.0, 62.61, 62.55, 33.0, 31.6, 24.9, 16.32, 16.26. The other characterization data matched the reported literature values.[1b-c]

Preparation of (*E*)-6-(4,4-diethoxybut-1-en-1-yl)-2,2-dimethyl-4*H*-1,3-dioxin -4-one (17) [2a]

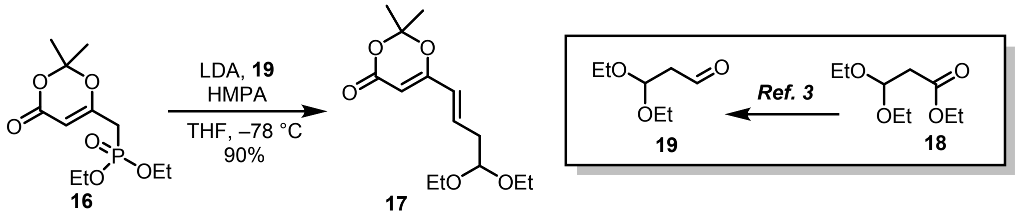

To a stirred solution of **16** (13.0 g, 46.7 mmol) in dry THF (160 mL) at −78 ºC under N_2 atmosphere was slowly added LDA (28.0 mL, 2 M in THF, 56.1 mmol). Then HMPA (10.1 g, 56.1 mmol) was added to the reaction mixture. The resulting mixture was allowed to keep stirring for 1.5 h at the same temperature. A solution of aldehyde **19** [6] (9.4 g, 80 w %, 51.4 mmol) in dry THF (100 mL) was added to the above mixture. The resulting mixture was allowed to warm up slowly to room temperature and was stirred overnight. The reaction was quenched by addition of aq. satd. NH_4Cl (80 mL) and then extracted with ethyl acetate (3 ×100 mL). The combined organic phase was washed with brine (3 ×100 mL), dried over Na_2SO_4, filtered and concentrated under reduced pressure. The residue was purified by silica gel column chromatography (Petroleum ether : Ethyl acetate = 8 : 1) to afford the product **17** (11.4 g, 90%) as a yellow oil.

Rf = 0.50 (silica, Petroleum ether : Ethyl acetate = 2 : 1). ^{1}H NMR (400 MHz, $CDCl_3$) δ 6. 51 – 6.43 (m, 1H), 5.94 (d, *J* = 15.6 Hz, 1H), 5.21 (s, 1H), 4.52 (t, *J* = 5.5 Hz, 1H), 3.61 (dq, *J* = 9.3, 7.1 Hz, 2H), 3.47 (dq, *J* = 9.4, 7.0 Hz, 2H), 2.77 – 2.35 (m, 2H), 1.65 (s, 6H), 1.16 (t, *J* = 7.1 Hz, 6H). ^{13}C NMR (100 MHz, $CDCl_3$) δ 163.2, 162.1, 136.8, 125.0, 106.5, 101.5, 94.0, 61.8, 37.5, 25.2, 15.4. HRMS (ESI): m/z calcd for $C_{14}H_{22}O_5Na$ $[M + Na]^+$: 293.1359, found: 293.1361.

Preparation of methyl (*E*)-7,7-diethoxy-3-oxohept-4-enoate and methyl (2*Z*,4*E*)-7,7-diethoxy-3-hydroxy-hepta-2,4-dienoate (13) [2b,2d]

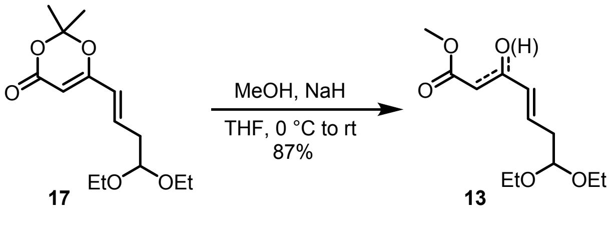

To a mixture of sodium hydride (60% in mineral oil, 8.10 g, 203 mmol, freshly washed with anhydrous hexane three times under nitrogen) in anhydrous THF (100 mL) at 0 °C under N_2 atmosphere was added methanol (16.5 mL, 407 mmol) via syringe over a period of 40 min. After stirring at room temperature for another 30 min, a solution of olefin **17** (11.0 g, 40.7 mmol) in THF (100 mL) was added dropwise. The resulting mixture was stirred at room temperature for another 40 min under nitrogen. A powder of NH_4Cl (21.8 g, 407 mmol) was added and the mixture was stirred for 20 min. The reaction mixture was diluted with ethyl acetate (200 mL) and water (120 mL). After separation of the organic layer, the aqueous layer was extracted with ethyl acetate (3 × 100 mL). The combined organic phase was washed with brine (3 ×100 mL), dried over Na_2SO_4, filtered and concentrated under reduced pressure. The residue was purified by silica gel column chromatography (Petroleum ether : Ethyl acetate = 10 : 1) to afford the product **13** (8.70 g, 87%) as a yellow oil.

Rf = 0.30 (silica, Petroleum ether : Ethyl acetate = 4 : 1). ^{1}H NMR (400 MHz, $CDCl_3$) δ 11.77 (s, 1H), 6.82 (dt, *J* = 15.8, 7.1 Hz, 2H), 6.60 (dt, *J* = 15.1, 7.3 Hz, 1H), 6.21 (d, *J* = 16.0 Hz, 2H), 5.94 – 5.81 (m, 1H), 5.00 (s, 1H), 4.56 (dt, *J* = 11.0, 5.6 Hz, 3H), 3.73 (s, 3H), 3.72 (s, 6H), 3.65 (dq, *J* = 9.3, 7.1 Hz, 5H), 3.60 (s, 4H), 3.50 (dq, *J* = 9.2, 7.0 Hz, 7H), 2.63 – 2.51 (m, 4H), 2.51 (t, *J* = 6.2 Hz, 2H), 1.19 (t, *J* = 7.1 Hz, 18H). ^{13}C NMR (100 MHz, $CDCl_3$) δ 192.0, 173.4, 169.3, 168.0, 144.5, 135.5, 131.8, 126.8, 101.8, 101.3, 90.3, 61.8, 61.6, 52.5, 51.4, 46.6, 37.5, 37.5, 15.4, 15.4. HRMS (ESI): m/z calcd for $C_{14}H_{20}O_5Na$ $[M + Na]^+$: 267.1203, found: 267.1205.

Preparation of 6-(1,3-Dioxolan-2-yl)-1,3-benzodioxole-5-carboxaldehyde (22) [4-5]

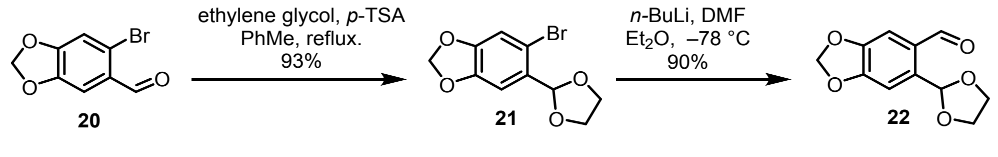

21 (> 20 g scale, 93%) was prepared by following literature[4a-b] procedure. white solid. ^{1}H NMR (400 MHz, $CDCl_3$) δ 7.07 (s, 1H), 7.00 (s, 1H), 6.01 (s, 1H), 5.98 (s, 2H), 4.18 – 4.01 (m, 4H). ^{13}C NMR (100 MHz, $CDCl_3$) δ 149.2, 147.6, 130.1, 114.1,

113.0, 107.8, 102.7, 102.1, 65.6. The other characterization data matched the reported literature values.[4c-e]

Improved synthetic method for the preparation of 22 based on literature [5]

To a stirred solution of **21** (24.0 g, 87.9 mmol) in Et_2O (250 mL) at −78 °C under N_2 atmosphere was slowly added *n*-BuLi (42.2 mL, 2.5 M in hexanes, 105 mmol) via syringe over a period of 45 min. The reaction mixture was stirred for additional 1 h. DMF (13.6 mL, 176 mmol) was added dropwise and the resulting mixture was stirred for 2 h at the same temperature. The reaction was allowed to warm up slowly to room temperature and then was stirred overnight. The reaction was quenched by addition of aq. satd. NH_4Cl (120 mL) and the mixture was extracted with ethyl acetate (3 × 100 mL). The combined organic phase was washed with brine (3 ×100 mL), dried over Na_2SO_4, filtered and concentrated under reduced pressure. The residue was purified by silica gel column chromatography (Petroleum ether : Dichloromethane = 1 : 2) to afford the product **22** (17.6 g, 90%) as a white solid.

Rf = 0.16 (silica, Dichloromethane). ^{1}H NMR (400 MHz, $CDCl_3$) δ 10.26 (s, 1H), 7.39 (s, 1H), 7.17 (s, 1H), 6.34 (s, 1H), 6.06 (s, 2H), 4.17 – 3.04 (m, 4H). ^{13}C NMR (100 MHz, $CDCl_3$) δ 189.3, 152.3, 148.7, 136.8, 129.7, 108.3, 107.0, 102.3, 100.4, 65.5. The other characterization data matched the reported literature values.[5]

Preparation of 5-(1,3-Dioxolan-2-yl)-6-[(1*E*)-2-nitroethenyl]-1,3-benzodioxole (14) [6]

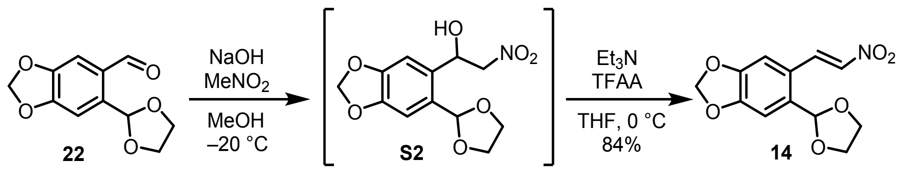

To a stirred solution of aldehyde **22** (12.0 g, 54.0 mmol) and nitromethane (3.5 mL, 64.8 mmol) in methanol (160 mL) was added an aqueous solution of sodium hydroxide (5.40 mL, 10 M, 54.0 mmol) at −20 ºC. The resulting mixture was then stirred for 2 h at the same temperature. When the starting material was consumed completely, acetic acid (3.10 mL, 54.0 mmol) was added to the mixture and stirred for 10 min. Then removed the solvent under reduced pressure and the residue was treated with

dichloromethane (200 mL) and half-saturated brine (100 mL). The organic layer was separated and the aqueous layer was extracted with dichloromethane (3 × 100 mL). The combined organic phase was dried over Na_2SO_4, filtered and concentrated under reduced pressure to afford the product **S2** (13.0 g, 45.9 mmol) as a faint yellow oil. To the solution of crude oil of **S2** in anhydrous THF (200 mL) and triethylamine (14.0 mL, 101 mmol) at 0 ºC under N_2 atmosphere was slowly added trifluoroacetic anhydride (7.10 mL, 50.5 mmol) and the resulting mixture was stirred for 15 min. The reaction was diluted with ice-water (100 mL) and dichloromethane (400 mL). After separation of the organic layer, the aqueous layer was extracted with dichloromethane (3 × 40 mL). The combined organic phase was washed with brine (3× 100 mL) and dried over Na_2SO_4. After filtration and the solvent was removed under reduced pressure, the residue was purified by silica gel column chromatography (Dichloromethane) to afford the product **14** (12.0 g, 84%,) as a yellow solid.

Rf = 0.60 (silica, Dichloromethane), Mp: 178−180 °C, (literature [3d]: Mp: 179−180 °C). ^{1}H NMR (400 MHz, $CDCl_3$) δ 8.43 (d, *J* = 13.5 Hz, 1H), 7.43 (d, *J* = 13.4 Hz, 1H), 7.16 (s, 1H), 7.00 (s, 1H), 6.06 (s, 2H), 5.95 (s, 1H), 4.21 – 4.05 (m, 4H). ^{13}C NMR (100 MHz, $CDCl_3$) δ 151.0, 148.9, 136.8, 136.0, 134.2, 123.0, 108.1, 106.8, 102.3, 101.3, 65.6. HRMS (ESI): m/z calcd for $C_{12}H_{11}NO_6Na$ [M + Na]$^+$: 288.0479, found: 288.0480.

Preparation of methyl (4*S*,5*R*,6*R*)-6-(6-(1,3-dioxolan-2-yl)benzo[*d*][1,3]dioxol -5-yl)-4-(2,2-diethoxyethyl)-2-hydroxy-5-nitrocyclohex-1-ene-1-carboxylate (12)

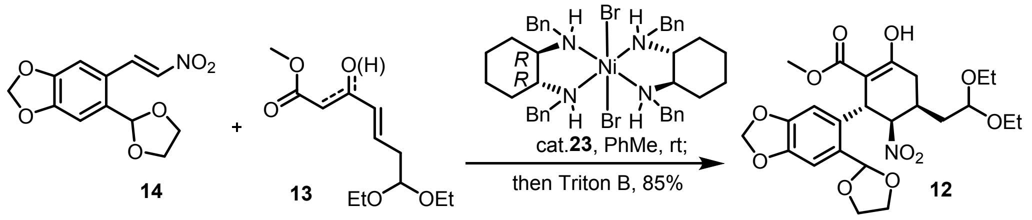

To a stirred solution of **13** (8.46 g, 34.6 mmol) and **14** (7.65 g, 28.9 mmol) in toluene (100 mL) were added Evans' catalyst (**23**)[7] (474 mg, 0.580 mmol). The resulting yellow mixture was stirred at room temperature for 96 h. After this time, the reaction mixture was diluted with THF (100 mL) and cooled to 0 ºC, Benzyltrimethylammonium

Hydroxide (11.4 mL, 40% in methanol, 28.9 mmol) was added and kept stirring for another 20 min. The reaction was quenched by addition of aq. satd. NH_4Cl (100 mL), after separation of organic layer, the aqueous layer was extracted with ethyl acetate (3 × 120 mL). The combined organic phase was washed with brine (3 × 80 mL), dried over Na_2SO_4, filtered and concentrated under reduced pressure. The residue was purified by silica gel column chromatography (Dichloromethane) to afford the product **12** (12.5 g, 85%) as a yellow oil.

Rf = 0.32 (silica, Dichloromethane). ^1H NMR (400 MHz, $CDCl_3$) δ 12.45 (s, 1H), 7.11 (s, 1H), 6.61 (s, 1H), 5.98 (s, 1H), 5.95 (s, 1H), 5.90 (s, 1H), 4.88 (s, 1H), 4.76 (s, 1H), 4.51 (t, J = 5.7 Hz, 1H), 4.17 – 3.98 (m, 4H), 3.55 – 3.49 (m, 5H), 3.43 – 3.35 (m, 1H), 3.29 – 3.21 (m, 1H), 2.57 – 2.53 (m, 1H), 2.43 – 2.31(m, 2H), 1.78 – 1.62 (m, 2H), 1.09 (t, J = 7.0 Hz, 3H), 0.95 (t, J = 7.0 Hz, 3H). ^{13}C NMR (100 MHz, $CDCl_3$) δ 172.1, 171.8, 148.2, 146.8, 134.0, 128.8, 108.3, 107.6, 101.5, 101.4, 100.5, 96.8, 87.9, 65.3, 65.2, 62.1, 61.9, 51.9, 39.1, 36.0, 31.0, 27.4, 15.3, 15.0. HRMS (ESI): m/z calcd for $C_{24}H_{31}NO_{11}Na$ $[M + Na]^+$: 532.1789, found: 532.1789.

Chiral HPLC analysis: 25 ºC; column: DAICEL Chiralcel AD-H (4.6mm × 250 mm); mobile phase: hexane / isopropanol = 90 / 10; flow rate:1.0 mL/min; detection, UV 250 nm; t_R for major isomer: 12.3 min; minor isomer: 15.8 min. The racemic sample was prepared by conducting the reaction with racemic catalyst **23** (prepared with a mixture of (1*R*, 2*R*) - (–) -1,2-diaminocyclo-hexane and (1*S*, 2*S*) - (+) -1,2-diaminocyclohexane, 1 : 1).

Preparation of methyl (4*S*,5*R*,6*R*)-6-(6-(1,3-dioxolan-2-yl)benzo[*d*][1,3] dioxol-5-yl)-2-(((benzyloxy)carbonyl)oxy)-4-(2,2-diethoxyethyl)-5-nitrocyclohex-1-ene-1-carboxylate (27)

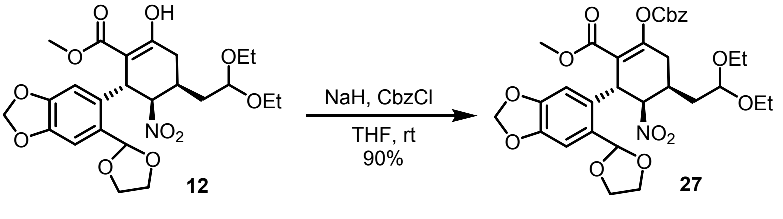

To a suspension of sodium hydride (60% in mineral oil, 51.6 mg, 2.13 mmol, freshly washed with anhydrous hexane three times under nitrogen) in anhydrous THF (20 mL) was added **12** (542 mg, 1.64 mmol in THF (30 mL) via syringe at room temperature.

After stirring at room temperature for 1.5 h, the solution of CbzCl (0.23 mL, 1.60 mmol) in THF (10 mL) was slowly added. The resulting mixture was stirred at room temperature for 1 h. After full consumption of the starting material, a powder of NH_4Cl (2.28 g, 42.6 mmol) was added and the mixture kept stirring for another 20 min. The reaction was quenched by addition of HCl (2 M, 5 mL), diluted with ethyl acetate (60 mL) and water (30 mL). The organic layer was separated and the aqueous layer was extracted with ethyl acetate (3 × 60 mL). The combined organic phase was washed with brine (3 × 60 mL), dried over Na_2SO_4, filtered and concentrated under reduced pressure. The residue was purified by silica gel column chromatography (Petroleum ether : Dichloromethane = 5 : 1) to afford the product **27** (618 mg, 90%) as white foam.

Rf = 0.20 (silica, Petroleum ether : Dichloromethane = 3 : 1). ^{1}H NMR (400 MHz, $CDCl_3$) δ 7.49 – 7.31 (m, 5H), 7.12 (s, 1H), 6.87 (s, 1H), 6.00 – 5.90 (m, 3H), 5.30 (s, 2H), 5.00 (s, 2H), 4.94 (d, J = 3.2 Hz, 2H), 4.54 (t, J = 5.8 Hz, 1H), 4.17 – 4.10 (m, 1H), 4.11 – 3.94 (m, 3H), 3.50 – 3.59 (m, 2H), 3.48 – 3.35 (m, 4H), 3.32 – 3.20 (m, 1H), 2.65 – 2.44 (m, 2H), 2.36 – 2.23 (m, 1H), 1.80 – 1.71 (m, 1H), 1.65 (dt, J = 13.7, 6.5 Hz, 1H), 1.11 (t, J = 7.0 Hz, 3H), 0.94 (t, J = 7.0 Hz, 3H). ^{13}C NMR (100 MHz, $CDCl_3$) δ 164.3, 155.6, 152.4, 148.4, 147.2, 134.9, 132.0, 129.2, 128.9, 128.8, 128.7, 128.3, 117.2, 109.1, 108.1, 101.8, 101.6, 100.5, 87.3, 70.7, 65.3, 65.2, 62.2, 62.1, 52.2, 41.0, 35.7, 31.2, 28.3, 15.4, 15.0. HRMS (ESI): m/z calcd for $C_{32}H_{37}NO_{13}$ $[M + Na]^+$: 666.2157, found: 666.2156.

Preparation of methyl (4*S*,5*R*,6*R*)-6-(6-(1,3-dioxolan-2-yl)benzo[*d*][1,3] dioxol-5-yl)-4-(2,2-diethoxyethyl)-1-(methoxycarbonyl)-5-nitrocyclohex-2-en-2-yl benzoate (11)

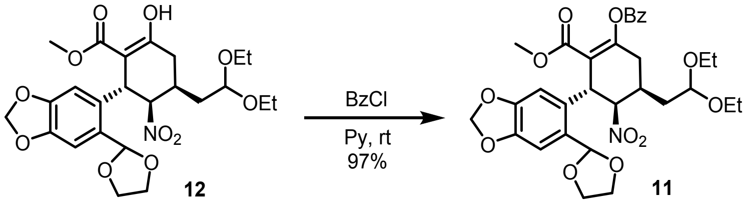

To a stirred solution of **12** (12.7 g, 24.9 mmol) in pyridine (150 mL) at 0 ºC under N_2 atmosphere was added Benzoyl chloride (5.74 mL, 49.9 mmol) dropwise. The reaction mixture was allowed to warm up to room temperature and stirred for another

10 h. The reaction was quenched by addition of HCl (2 M, 20 mL) and extracted with Dichloromethane (3 × 150 mL). The combined organic phase was washed with aq. satd. NH_4Cl (3 × 100 mL) and brine (3 × 100 mL), dried over Na_2SO_4, filtered and concentrated under reduced pressure. The residue was purified by silica gel column chromatography (Dichloromethane : Petroleum ether : Ethyl acetate = 10 : 5 : 1) to afford product **11** (14.87 g, 97%, ee* = 90%) as a faint yellow oil. It was found that a little amount of racemic crystal could be filtered off after recrystallization of the column chromatography-purified product **11** from ethyl acetate/hexane (v/v =1:10, 1.1 mol/L, 220 mL, 14.87g) at room temperature for 4 h, and then vacuum evaporation of the filtrate provided a faint yellow oil with higher optical purity (11.9 g, 78%, ee* = 99.1%).

Rf = 0.30 (Dichloromethane : Petroleum ether : Ethyl acetate = 10 : 5 : 1), Mp: 172−174 °C, $[\alpha]^{20}_{D}$ = –78.0° (c = 1.0, $CHCl_3$). ^1H NMR (400 MHz, $CDCl_3$) δ 8.15 (d, J = 7.7 Hz, 2H), 7.63 (t, J = 7.4 Hz, 1H), 7.50 (t, J = 7.7 Hz, 2H), 7.13 (s, 1H), 7.07 (s, 1H), 6.00 (s, 1H), 5.97 – 5.91 (m, 2H), 5.06 (s, 1H), 5.02 – 4.97 (m, 1H), 4.56 (t, J = 5.8 Hz, 1H), 4.15 (t, J = 5.8 Hz, 1H), 4.10 – 4.01 (m, 3H), 3.59 – 3.53 (m, 2H), 3.44 – 3.40 (m, 4H), 3.29 – 3.25 (m, 1H), 2.71 – 2.59 (m, 2H), 2.40 – 2.36 (m, 1H), 1.78 – 1.75 (m, 1H), 1.70 – 1.65 (m, 1H), 1.12 (t, J = 7.0 Hz, 3H), 0.96 (t, J = 7.0 Hz, 3H). ^{13}C NMR (100 MHz, $CDCl_3$) δ 164.9, 164.4, 156.5, 148.5, 147.2, 133.8, 132.2, 130.4, 129.3, 129.2, 128.7, 117.1, 109.3, 108.0, 101.9, 101.6, 100.6, 87.6, 65.3, 65.2, 62.2, 52.1, 41.3, 35.8, 31.7, 28.3, 15.3, 15.0. HRMS (ESI): m/z calcd for $C_{31}H_{35}NO_{12}Na$ $[M + Na]^+$: 636.2051, found: 636.2051.

Chiral HPLC analysis: 25 ºC; column: DAICEL Chiralcel AD-H (4.6mm × 250 mm); mobile phase: Hexane / Isopropanol (80 / 20); flow rate: 1.0 mL/min; detection, UV 254 nm; t_R for major isomer: 17.3 min; minor isomer: 14.5 min.

Single crystal of **11** growed from Hexane at room temperature, its crystal structure was determined by single - crystal X-ray diffraction (CCDC: 2019555).

One-pot operation for the preparation of (3^aR,$3^{a1}R$,12^bS)-3^a,3^{a1},4,5,7,12^b-hexahydro-1*H*-[1,3] dioxolo[4,5-*j*]pyrrolo[3,2,1-*de*]phenanthridin-2(3*H*)-one (10)

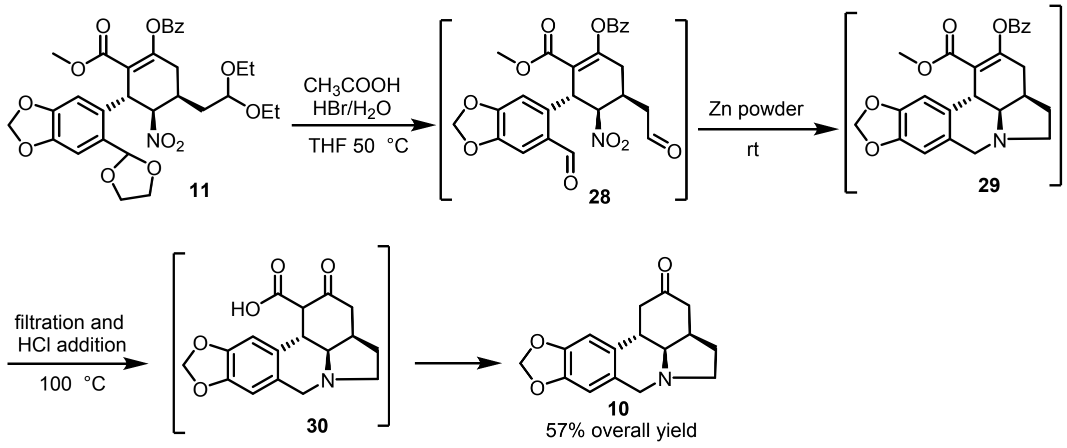

To a stirred solution of **11** (6.14 g, 10.0 mmol) in the mixture of THF (40 mL), acetic acid (60 mL) and H_2O (50 mL) at room temperature was slowly added HBr (2.4 mL, 33% in CH_3COOH). The reaction mixture was stirred at 50 ºC for additional 2 h under N_2 atmosphere before being cooled to room temperature. Then zinc powder (26.2 g, 400 mmol) was added, the resulting mixture was stirred overnight at room temperature under N_2 atmosphere. After Solid substances were filtered off from the reaction mixture, HCl (8 M, 125 mL) was added to the filtrate and the mixture was heated to 100 ºC, and kept stirring for 16 h before being cooled to room temperature. The reaction was quenched by addition of solid K_2CO_3 (276 g, 2.0 mol). The resulting mixture was extracted with ethyl acetate (3 × 150 mL). The combined organic phase was washed with brine (3 × 60 mL), dried over Na_2SO_4, filtered and concentrated under reduced pressure. The residue was purified by silica gel column chromatography (Ethyl acetate : Triethylamine = 100 : 1) to afford product **10** (1.56 g, 57% overall yield) as a faint yellow solid.

Rf = 0.28 (silica, Ethyl acetate : Triethylamine = 60 : 1), Mp: 127−129 °C, $[\alpha]^{20}_D$ = –266.2° (c = 1.0, $CHCl_3$). ^1H NMR (400 MHz, $CDCl_3$) δ 6.61 (s, 2H), 5.92 (s, 2H), 4.08 (d, J = 14.2 Hz, 1H), 3.81 (d, J = 14.2 Hz, 1H), 3.25 – 3.20 (m, 1H), 3.12 (t, J = 7.1 Hz, 1H), 2.91 (dd, J = 18.3, 4.1 Hz, 1H), 2.61 – 2.49 (m, 5H), 2.45 – 2.15 (m, 1H), 2.11 (dd, J = 18.2, 13.9 Hz, 1H), 1.75 – 1.67 (m, 1H). ^{13}C NMR (100 MHz, $CDCl_3$) δ 212.0, 146.5, 146.3, 131.7, 129.0, 107.3, 105.2, 101.1, 64.7, 55.8, 53.5, 44.2, 39.7, 36.6, 33.6, 32.8. HRMS (ESI): m/z calcd for $C_{16}H_{18}NO_3$ $[M + H]^+$: 272.1281, found: 272.1283.

Preparation of (3aR,3a1R,12bS)-3a,3a1,4,5,7,12b-hexahydro-3H-[1,3]dioxolo[4,5-j]pyrrolo[3,2,1-de]phenanthridin-2-yl trifluoromethanesulfonate (31)

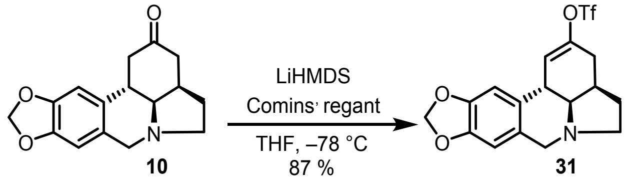

To a stirred solution of **10** (1.25 g, 4.61mmol) in dry THF (30 mL) at −78 °C under N_2 atmosphere was slowly added LiHMDS (9.21 mL, 1.0 M in THF, 9.21 mmol). The reaction mixture was stirred for additional 1 h. Then Comins' reagent [8] (2.71g, 6.91 mmol) in THF (20 mL) was added dropwise. After stirring for 1 h, the reaction was quenched by addition of aq. satd. NH_4Cl (30 mL) and the resulting mixture was extracted with ethyl acetate (3 × 80 mL). The combined organic phase was washed with brine (3 × 60 mL), dried over Na_2SO_4, filtered and concentrated under reduced pressure. The residue was purified by silica gel column chromatography (Petroleum ether : Triethylamine : Ethyl acetate = 30 : 10 : 1) to afford product **31** (1.62 g, 87%) as a tans oil.

Rf = 0.28 (silica, Petroleum ether : Ethyl acetate: Triethylamine = 30 : 1 : 10), $[\alpha]^{20}_{D}$ = –110.9° (c = 0.82, $CHCl_3$). ^{1}H NMR (400 MHz, $CDCl_3$) δ 6.74 (s, 1H), 6.63 (s, 1H), 6.25 (t, J = 2.9 Hz, 1H), 5.93 (s, 2H), 4.05 (d, J = 14.8 Hz, 1H), 3.87 (d, J = 14.8 Hz, 1H), 3.33 – 3.24 (m, 1H), 3.06 – 2.93 (m, 2H), 2.76 – 2.59 (m, 3H), 2.48 – 2.33 (m, 1H), 2.23 – 2.11 (m, 1H), 1.70 (dq, J = 12.2, 8.1 Hz, 1H); ^{13}C NMR (100 MHz, $CDCl_3$) δ 150.6, 146.6, 146.2, 130.5, 128.9, 123.5, 120.3, 117.6, 117.1, 113.9, 107.5, 104.7, 101.1, 64.9, 55.0, 53.9, 36.8, 36.3, 32.8, 31.2. HRMS (ESI): m/z calcd for $C_{17}H_{17}SF_3NO_5$ $[M + H]^+$: 404.0774, found: 404.0772.

Preparation of (3aS,3a1R,12bS)-3a,3a1,4,5,7,12b-hexahydro-3H-[1,3]dioxolo [4,5-j]-pyrrolo[3,2,1-de]phenanthridine (9)

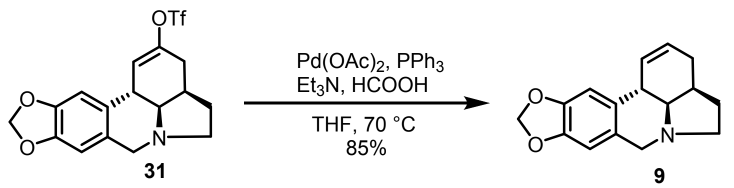

To a stirred solution of **31** (2.30 g, 5.70 mmol) in THF (80 ml) were orderly added

PPh$_3$ (598 mg, 2.28 mmol), Pd(OAc)$_2$ (256 mg, 1.14 mmol), triethylamine (7.90 mL, 57.0 mmol) and HCO_2H (2.13 mL, 57.0 mmol) at room temperature under N_2 atmosphere. The reaction mixture was heated to 70 ℃ and stirred for additional 1.5 h. The reaction was quenched by addition of aq. satd. $NaHCO_3$ (40 mL) and the resulting mixture was extracted with ethyl acetate (3 × 80 mL). The combined organic phase was washed with brine (3 × 40 mL), dried over Na_2SO_4, filtered and concentrated under reduced pressure. The residue was purified by silica gel column chromatography (Petroleum ether : Triethylamine : Ethyl acetate = 50 : 10 : 1) to afford product **9** (1.24 g, 85%) as a tans oil.

Rf = 0.45 (silica, Petroleum ether : Triethylamine : Ethyl acetate = 30 : 10 : 1), $[\alpha]^{20}_{D}$ = –212.3° (c = 0.46, $CHCl_3$). ^{1}H NMR (400 MHz, $CDCl_3$) δ 6.88 (s, 1H), 6.61 (s, 1H), 6.29 (dt, *J* = 9.3, 3.3 Hz, 1H), 6.06 (td, *J* = 6.5, 3.3 Hz, 1H), 3.91 (s, 2H), 3.13 (d, *J* = 9.4 Hz, 1H), 2.99 – 2.95 (m, 1H), 2.74 (dd, *J* = 8.52, 8.52 Hz, 1H), 2.55 – 2.43 (m, 2H), 2.29 (dt, *J* = 17.04, 16.96 Hz, 1H), 2.13 – 2.06 (m, 1H), 1.93 – 1.85 (m, 1H), 1.68 – 1.59 (m, 1H). ^{13}C NMR (100 MHz, $CDCl_3$) δ 146.3, 145.6, 132.8, 130.7, 129.2, 128.5, 107.4, 105.1, 100.8, 66.3, 55.5, 53.9, 38.2, 36.3, 32.0, 30.2. HRMS (ESI): m/z calcd for $C_{16}H_{18}NO_2$ $[M + H]^+$: 256.1332, found: 256.1332.

Synthesis of (–)-zephyranthine

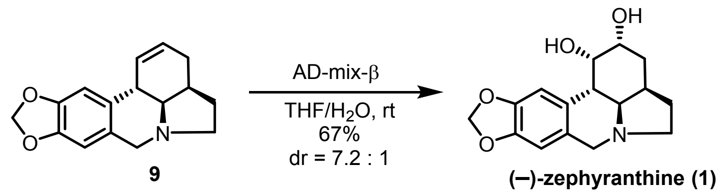

To a stirred solution of **9** (1.02 g, 4.00 mmol) in THF/H_2O (40 mL, v/v = 1 : 1) was added AD-mix-β [9] (4.67g, 6.00 mmol) at room temperature. The reaction mixture was stirred for additional 3.5 h before quenched by addition of aq. satd. $NaHCO_3$, and the resulting mixture was extracted with THF (3 × 30 mL). The combined organic phase was dried over Na_2SO_4, filtered and concentrated under reduced pressure. The residue was purified by silica gel column chromatography (Dichloromethane : Methanol : Triethylamine = 40 : 5 : 1) to afford (–)-zephyranthine (770 mg, 67%) as a white solid.

Rf = 0.48 (silica, Dichloromethane : Methanol : Triethylamine = 40 : 5 : 1), Mp: 168−170 °C, $[\alpha]^{20}_{D}$ = – 31.3° (c = 0.62, MeOH). ^{1}H NMR (400 MHz, CD_3OD) δ 6.96 (s, 1H), 6.64 (s, 1H), 5.89 (d, *J* = 4.3 Hz, 2H), 4.48 (s, 1H), 4.14 (d, *J* = 15.4 Hz, 1H), 3.97 – 3.82 (m, 1H), 3.70 (d, *J* = 15.4 Hz, 1H), 3.25 (q, *J* = 8.7 Hz, 1H), 3.05 (dd, *J* = 10.8, 6.2 Hz, 1H), 2.84 (td, *J* = 9.4, 5.1 Hz, 1H), 2.63 – 2.39 (m, 2H), 2.14 – 2.04 (m, 1H), 1.99 – 1.82 (m, 3H). ^{13}C NMR (100 MHz, CD_3OD) δ 148.1, 147.2, 132.4, 128.9, 107.7, 106.3, 102.1, 69.9, 69.7, 59.9, 54.9, 54.1, 38.4, 37.0, 29.7, 28.1. HRMS (ESI): m/z calcd for $C_{16}H_{20}NO_4$ $[M + H]^+$: 290.1387, found: 290.1384.

For comparison:

J. Bastida[10]

(−)-zephyranthine $[\alpha]^{20}_{D}$ = −30.6 (c = 0.56, Methanol)

^{1}H NMR (500 MHz, CD_3OD) δ 7.00 (s, 1H), 6.71 (s, 1H), 5.93 – 5.91 (d, *J* = 1.5, 2H), 4.51 (s, 1H), 4.27 (d, *J* = 15.0, 1H), 3.90 (ddd, *J* = 11.5, 5.0, 2.5, 1H), 3.81 (d, *J* = 15.0, 1H), 3.37 (td, *J* = 10.0, 9.5, 1H), 3.19 (dd, *J* = 11.0, 6.5, 1H), 3.02 (ddd, *J* = 10.0, 9.5, 3.0, 1H), 2.66 (d, *J* = 11.0, 1H), 2.58 (qd, *J* = 6.5, 1.5, 1H), 2.11 (ddd, *J* = 13.5, 11.5, 6.5, 1H), 2.04 (m, 1H), 1.98 (m, 1H), 1.89 (ddd, *J* = 13.5, 5.0, 1.5, 1H).

^{13}C NMR (75 MHz, CD_3OD) δ = 148.6, 147.4, 132.6, 127.4, 107.9, 106.5, 102.3, 69.8, 69.4, 61.4, 55.6, 54.1, 38.4, 37.4, 29.2, 28.3

Sun [11]

(−)-zephyranthine $[\alpha]^{20}_{D}$ = −28.9 (c = 0.40, Methanol).

^{1}H NMR (400 MHz, $CDCl_3$) δ = 6.88 (s, 1H), 6.59 (s, 1H), 5.92 (d, *J* = 1.2, 1H), 5.90 (d, *J* = 1.2, 1H), 4.59 (s, 1H), 4.22 (d, *J* = 16.4, 1H), 4.02 – 3.97 (m, 1H), 3.75 (d, *J* = 16.8, 1H), 3.24 (td, *J* = 8.8, 7.2, 1H), 3.06 (dd, *J* = 10.4, 6.4, 1H), 2.77 (td, *J* = 10.0, 4.4, 1H), 2.64 – 2.54 (m, 1H), 2.48 (d, *J* = 10.4, 1H), 2.08 – 1.87 (m, 5H), 1.77 – 1.69 (m, 1H).

^{13}C NMR (100 MHz, $CDCl_3$) δ = 146.4, 145.9, 129.4, 129.1, 107.1, 104.4, 100.8, 68.9, 68.8, 57.5, 53.0, 52.9, 36.4, 35.0, 30.1, 27.2.

Chida[12]

(−)-zephyranthine[α] $^{29}{}_{D}$ = −30.4 (c = 0.56, Methanol).

^{1}H NMR (500 MHz, $CDCl_3$) δ = 6.88 (s, 1H), 6.59 (s, 1H), 5.92 (d, *J* = 1.5, 1H), 5.91 (d, *J* = 1.5, 1H), 4.60 (dd, *J* = 3.2, 2.5, 1H), 4.21 (d, *J* = 16.4, 1H), 4.99 (ddd, *J* = 10.6, 5.2, 3.2, 1H), 3.74 (d, *J* = 16.4, 1H), 3.24 (ddd, *J* = 10.1, 8.9, 6.9, 1H), 3.06 (dd, *J* = 10.3, 6.0, 1H), 2.77 (ddd, *J* = 10.1, 10.1, 4.3, 1H), 2.63 – 2.55 (m, 1H), 2.48 (d, *J* = 10.3, 1H), 2.04 (ddd, *J* = 13.5, 10.6, 6.1, 1H), 1.99 (dddd, *J* = 13.2, 9.2, 8.9, 4.3, 1H), 1.90 (ddd, *J* = 13.5, 5.2, 2.9, 1H), 1.77 –1.68, (m, 1H).

^{13}C NMR (125 MHz, $CDCl_3$) δ = 146.5, 146.1, 129.6, 129.3, 107.2, 104.5, 100.9, 69.1, 68.9, 57.6, 53.2, 53.0, 36.6, 35.1, 30.3, 27.4.

Preparation of (3aS,3a1R,12bS)-3,3a,3a1,4,5,12b-hexahydro-7H-[1,3]dioxolo[4,5-j] pyrrolo[3,2,1-de]phenanthridin-7-one (32) [13]

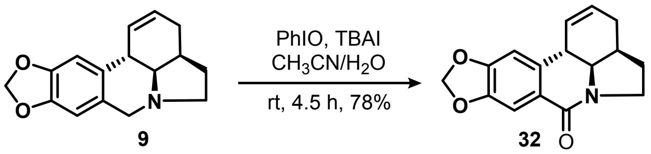

To a stirred solution of **9** (52.0 mg, 0.203 mmol) in CH_3CN/H_2O (10 mL, V/V = 9 : 1) was added PhIO (98.6 mg, 0.449 mmol) and TBAI (15.1 mg, 0.041 mmol) at room temperature. The reaction mixture was stirred for additional 4.5 h before quenched by addition of aq. satd. $Na_2S_2O_3$ (10 mL), and the resulting mixture was extracted with DCM (3 × 40 mL). The combined organic phase was dried over Na_2SO_4, filtered and concentrated under reduced pressure. The residue was purified by silica gel column chromatography (Dichloromethane : Petroleum ether : Ethyl acetate = 2 : 1 : 1) to afford **32** (43.0 mg, 78%) as a white solid.

Rf = 0.32 (silica, Dichloromethane : Petroleum ether : Ethyl acetate = 2 : 1 : 1), Mp: 145−147 °C, [α] $^{20}{}_{D}$ = – 264.5° (c = 1.0, $CHCl_3$). ^{1}H NMR (400 MHz, $CDCl_3$) δ 7.53 (s, 1H), 6.90 (s, 1H), 6.30 (dt, *J* = 9.2, 3.2 Hz, 1H), 6.20 – 6.15 (m, 1H), 6.01 (s, 2H), 4.19 (dd, *J* = 11.8, 7.7 Hz, 1H), 3.49 (dd, *J* = 12.0, 10.3 Hz, 1H), 3.33 – 3.17 (m, 2H), 2.73 – 2.66 (m, 1H), 2.61 – 2.49 (m, 1H), 2.23 (td, *J* = 12.6, 7.2, 5.5 Hz, 1H), 1.95 – 1.87 (m, 1H), 1.78 – 1.65 (m, 1H). ^{13}C NMR (100 MHz, $CDCl_3$) δ 162.4, 150.6, 146.7, 135.4, 131.7, 126.8, 125.5, 109.2, 104.2, 101.7, 62.0, 45.5, 39.0, 36.4, 32.9, 29.6.

HRMS (ESI): m/z calcd for $C_{16}H_{15}NO_3$ $[M + Na]^+$: 292.0944, found: 292.0944.

Preparation of (3^aR,$3^{a1}R$,12^bS)-3^a,3^{a1},4,5,7,12^b-hexahydro-1*H*-[1,3]dioxolo[4,5-*j*] pyrrolo[3,2,1-*de*]phenanthridine (36)

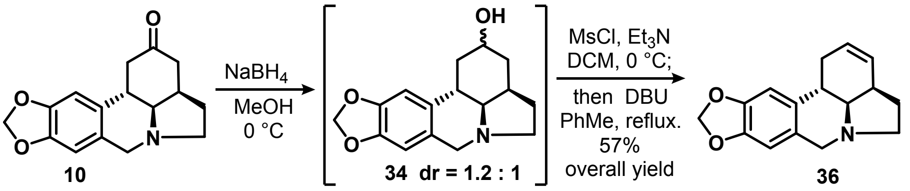

To a stirred solution of **10** (54.0 mg, 0.200 mmol) in methanol (15 mL) was added $NaBH_4$ (11.3 mg, 0.300 mmol) at 0 °C. The reaction mixture was stirred for additional 1 h at the same temperature before quenched by addition of water (10 mL), then the mixture was extracted with dichloromethane (4 × 40 mL). The combined organic phase was dried over Na_2SO_4, filtered and concentrated under reduced pressure to afford the crude product **34** (54.0 mg) as a white solid. The crude product **34** (without further purification) and triethylamine (0.14 mL, 0.990 mmol) were dissolved in dichloromethane (20 mL), and the resulting mixture was stirred for another 10 min at 0 °C. Then MsCl (0.0300 mL, 0.400 mmol) was slowly added and the mixture was stirred for 3 h before ice-cold water was added. After addition of HCl (2 M, 0.5 mL), the reaction mixture was neutralized with solid K_2CO_3 to pH = 8.0 and extracted with dichloromethane (4 × 30 mL). The combined organic phase was dried over Na_2SO_4, filtered and concentrated under reduced pressure to afford the crude mesylate product (96.0 mg) as a brown yellow oil. After that, to the solution of the above crude product (without further purification) in toluene (10 mL) was added DBU (1 mL) and DMF (2 mL), the reaction mixture was refluxed for 6 h under N_2 atmosphere before cooled to room temperature. The mixture was diluted with dichloromethane (30 mL), and then aq. satd. K_2CO_3 was added. The organic layer was separated, and the aqueous layer was extracted with dichloromethane (4 × 20 mL). The combined organic phase was dried over Na_2SO_4, filtered and concentrated under reduced pressure, the residue was purified by silica gel column chromatography (Ethyl acetate : Triethylamine = 200 : 1) to afford product **36** (29.0 mg, 57%) as a light yellow solid.

Rf = 0.18 (silica, Petroleum ether : Ethyl acetate :Triethylamine = 20 : 10 : 1), Mp: 112−115 °C, $[\alpha]^{20}_{D}$ = – 106.2° (c = 0.39, $CHCl_3$), ^1H NMR (400 MHz, $CDCl_3$) δ 6.72 (s, 1H), 6.61 (s, 1H), 5.90 (q, J = 1.5 Hz, 2H), 5.87 – 5.79 (m, 2H), 4.24 (d, J = 15.7 Hz, 1H), 3.78 (d, J = 15.7 Hz, 1H), 3.24 –3.18 (m, 1H), 2.95 – 2.79 (m, 3H), 2.65 (dt, J = 16.4, 5.0 Hz, 1H), 2.52 (td, J = 10.9, 4.4 Hz, 1H), 2.18 – 2.09 (m, 1H), 2.03 – 1.94 (m, 1H), 1.65 – 1.56 (m, 1H). ^{13}C NMR (101 MHz, $CDCl_3$) δ 146.4, 145.7, 132.7, 130.1, 128.8, 125.7, 106.8, 104.9, 100. 8, 61.5, 52.5, 52.4, 39.71, 30.4, 29.9, 27.5. HRMS (ESI): m/z calcd for $C_{16}H_{18}NO_2$ $[M + H]^+$: 256.1332, found: 256.1332.

Preparation of methyl (4*S*,5*R*,6*R*)-6-(6-(1,3-dioxolan-2-yl)benzo[*d*][1,3]dioxol -5-yl)-4-(2,2-diethoxyethyl)-5-nitro-2-(((trifluoromethyl)sulfonyl)oxy)cyclohex-1-ene-1-carboxylate (40)

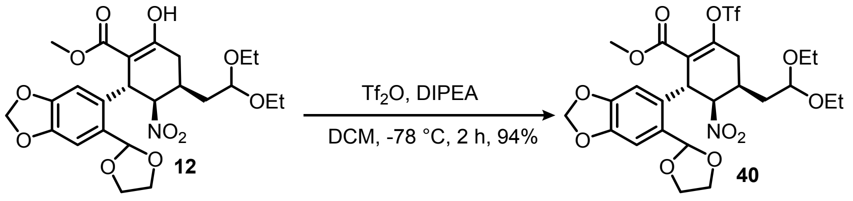

To a stirred solution of **12** (4.00 g, 7.60 mmol) and DIPEA (7.70 mL, 45.8 mmol) in dry DCM (40 mL) at −78 ºC under N_2 atmosphere was slowly added Tf_2O (3.90 mL, 22.9 mmol). The resulting mixture was then stirred for 2 h at the same temperature. The reaction was quenched by addition of aq. satd. $NaHCO_3$ (20 mL) and then extracted with DCM (3 × 40 mL). The combined organic phase was washed with brine (3 × 40 mL), dried over Na_2SO_4, filtered and concentrated under reduced pressure. The residue was purified by silica gel column chromatography (Petroleum ether : Dichloromethane = 2 : 1) to afford product **40** (4.70 g, 94%) as a tans oil.

Rf = 0.48 (silica, PE : DCM = 1 : 1); ^1H NMR (400 MHz, $CDCl_3$) δ 7.11 (s, 1H), 6.63 (s, 1H), 5.97 (d, J = 1.4 Hz, 1H), 5.94 (s, 1H), 5.90 (s, 1H), 5.07 (s, 1H), 5.03 – 4.79 (m, 1H), 4.53 (dd, J = 6.3, 4.8 Hz, 1H), 4.14 – 4.09 (m, 1H), 4.107 – 3.98 (m, 3H), 3.68 (s, 3H), 3.62 – 3.50 (m, 2H), 3.41 – 3.37 (m, 1H), 3.31–3.24 (m, 1H), 2.75 (dd, J = 18.5, 5.6 Hz, 1H), 2.55 – 2.50 (m, 1H), 2.41 – 2.33 (m, 1H), 1.80 – 1.74 (m, 1H), 1.71

– 1.56 (m, 1H), 1.11 (t, J = 7.1 Hz, 3H), 0.96 (t, J = 7.0 Hz, 3H). ^{13}C NMR (101 MHz, $CDCl_3$) δ 163.5, 151.6, 148.4, 147.5, 130.3, 129.4, 123.2, 121.9, 120.0, 116.7, 113.7, 108.6, 102.0, 101.8, 100.5, 87.1, 65.3, 65.2, 62.4, 62.2, 52.6, 41.8, 35.6, 30.7, 29.8, 28.8, 15.3, 14.9. HRMS (ESI): m/z calcd for $C_{25}H_{30}NO_{13}SF_3Na$ $[M + Na]^+$: 646.1282; found: 646.1286.

Preparation of methyl (3^a*R*,3^a1*R*,12^b*R*)-2-(((trifluoromethyl)sulfonyl)oxy)-3^a,3^a1,4,5,7,12^b-hexahydro-3H-[1,3]dioxolo[4,5-*j*]pyrrolo[3,2,1-*de*]phenanthridine-1-carboxylate (41)

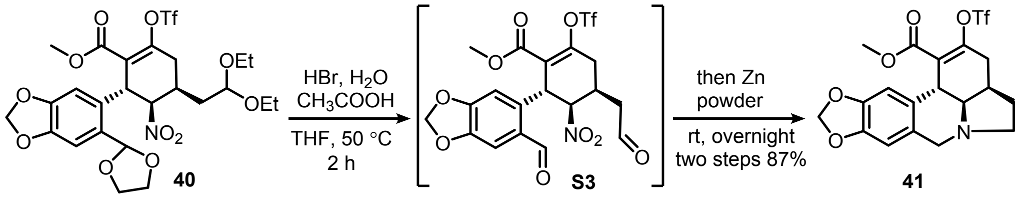

To a stirred solution of **40** (3.50 g, 5.50 mmol) in the mixture of THF (20 mL), acetic acid (30 mL) and H_2O (20 mL) at room temperature was slowly added HBr (1.00 mL, 33% in CH_3COOH). The reaction mixture was stirred at 50 ºC for additional 2 h under N_2 atmosphere before being cooled to room temperature to give **S3**. Then zinc powder (14.3 g, 218 mmol) was slowly added, the resulting mixture was stirred overnight at room temperature under N_2 atmosphere. The reaction was quenched by addition of solid K_2CO_3 (69.0 g, 0.500 mol). The resulting mixture was diluted with ethyl acetate (150 mL) and water (120 mL), After separation of the organic layer, the aqueous layer was extracted with ethyl acetate (3 × 100 mL). The combined organic phase was washed with brine (3 × 60 mL), dried over Na_2SO_4, filtered and concentrated under reduced pressure. The residue was purified by silica gel column chromatography (Ethyl acetate : Triethylamine = 100 : 1) to afford product **41** (1.87 g, 87% overall yield) as a faint yellow oil.

Rf = 0.28 (silica, EA : Et_3N = 40 : 1); ^1H NMR (400 MHz, $CDCl_3$) δ 6.61 (s, 1H), 6.43 (d, J = 0.9 Hz, 1H), 5.91 (q, J = 1.5 Hz, 2H), 3.98 (dd, J = 13.8, 1.2 Hz, 1H), 3.86 (s, 1H), 3.82 (s, 3H), 3.57 (d, J = 9.4 Hz, 1H), 3.09 – 2.88 (m, 2H), 2.87 – 2.71 (m, 2H), 2.69 – 2.55 (m, 1H), 2.47 (td, J = 16.7, 6.9, 1.6 Hz, 1H), 2.12 –2.08 (m, 1H), 1.76 –1.69

(m, 1H). ^{13}C NMR (101 MHz, $CDCl_3$) δ 164.8, 150.5, 146.4, 146.2, 129.4, 129.2, 125.5, 123.2, 120.0, 116.8, 113.7, 107.7, 105.8, 101.1, 66.0, 55.7, 54.6, 52.6, 40.6, 36.5, 32.9, 30.6. HRMS (ESI): m/z calcd for $C_{19}H_{19}F_3NO_7S$ $[M + H]^+$: 462.0829; found: 462.0831.

Preparation of methyl (3ᵃ*S*,3ᵃ¹*R*,12ᵇ*R*)-3ᵃ,3ᵃ¹,4,5,7,12ᵇ-hexahydro-3H-[1,3]dioxolo [4,5-*j*]pyrrolo[3,2,1-de]phenanthridine-1-carboxylate (37)

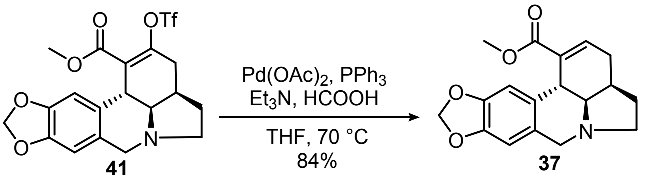

To a stirred solution of **41** (2.14 g, 4.64 mmol) in THF (80 ml) were orderly added PPh_3 (804 mg, 0.696 mmol), $Pd(OAc)_2$ (238 mg, 1.06 mmol), triethylamine (7.72 mL, 55.7 mmol) and HCO_2H (2.37 mL, 55.7 mmol) at room temperature. The reaction mixture was heated to 70 ℃ and stirred for additional 2 h under N_2 atmosphere. The reaction was quenched by addition of aq. satd. $NaHCO_3$ (40 mL) and the resulting mixture was extracted with ethyl acetate (3 × 80 mL). The combined organic phase was washed with brine (3 × 50 mL), dried over Na_2SO_4, filtered and concentrated under reduced pressure. The residue was purified by silica gel column chromatography (Ethyl acetate: Triethylamine = 70 : 1) to afford product **37** (1.14 g, 84%) as a tans oil.

Rf = 0.34 (silica, EA : Et_3N = 30 : 1); ^1H NMR (400 MHz, $CDCl_3$) δ 6.91 (td, *J* = 4.6, 2.6 Hz, 1H), 6.65 (s, 1H), 6.38 (d, *J* = 0.9 Hz, 1H), 6.01 – 5.52 (m, 2H), 4.11 (d, *J* = 13.9 Hz, 1H), 3.76 (s, 3H), 3.75 (d, *J* = 13.8 Hz, 1H) 3.37 (dd, *J* = 9.3, 2.6 Hz, 1H), 3.19 (td, *J* = 9.7, 7.1 Hz, 1H), 2.88 (td, *J* = 9.5, 2.1 Hz, 1H), 2.80 – 2.54 (m, 2H), 2.45 – 2.35 (m, 1H), 2.31 – 2.24 (m, 1H), 2.04 – 1.95 (m, 1H), 1.74 – 1.63 (m, 1H). ^{13}C NMR (101 MHz, $CDCl_3$) δ 168.6, 146.1, 145.4, 140.2, 133.6, 131.2, 129.4, 107.8, 105.8, 100.9, 56.5, 55.3, 51.9, 37.2, 34.8, 30.4, 28.6; HRMS (ESI): m/z calcd for $C_{18}H_{20}NO_4$ $[M + H]^+$: 314.1387; found: 314.1387.

Attempt to obtain intermediate 9 through a deester process[14]

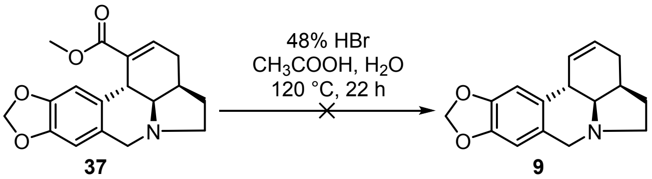

37 9

To a stirred solution of **37** (31.3 mg, 0.1 mmol) in CH_3COOH (135 μL) was added 48% HBr (270 μL) and H_2O (65μL) at room temperature. The reaction mixture was heated to 120 ℃ and stirred for 22 h under N_2 atmosphere. TLC analysis indicated that **37** decomposed under this condition and no product **9** was formed.

Attempt to obtain intermediate 9 through an ester hydrolysis and subsequent tandem decarboxylation process

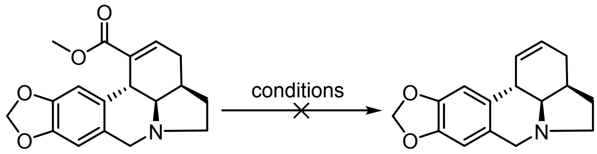

37 9

Table S1 Optimization of conditions for ester hydrolysis and decarboxylation[a, b]

Entry	Solvent	reagent	Temp. (°C)	Result[c]
1	MeOH	KOH then CH_3COOH	0 to 60	failed
2	MeOH	K_2CO_3 then CH_3COOH	20 to 80	failed
3	EtOH	KOH then CH_3COOH	0 to 60	failed
4	EtOH	K_2CO_3 then CH_3COOH	20 to 80	failed
5	THF	K_2CO_3 then CH_3COOH	20 to 80	failed
6	THF	K_2CO_3 then HCl (2 N)	20 to 80	failed

[a] Reactions were conducted on a 0.1 mmol scale using 1.0 equiv. **37**, 2.0 equiv. base (aqueous, 2.0 mol/L) and 2.0 mL solvent. [b] The reaction mixture of **37**, base and solvent was stirred at 0 °C (KOH as the base) or 20 °C (K_2CO_3 as the base). Acid was added and the reaction mixture was heated until the starting material (**37**) had been completely consumed as judged by TLC analysis. [c] Compound **37** was found to be unstable under basic conditions and no product **9** was obtained with all the above-mentioned conditions

Attempt to obtain intermediate 9 through a deformylation process[15]

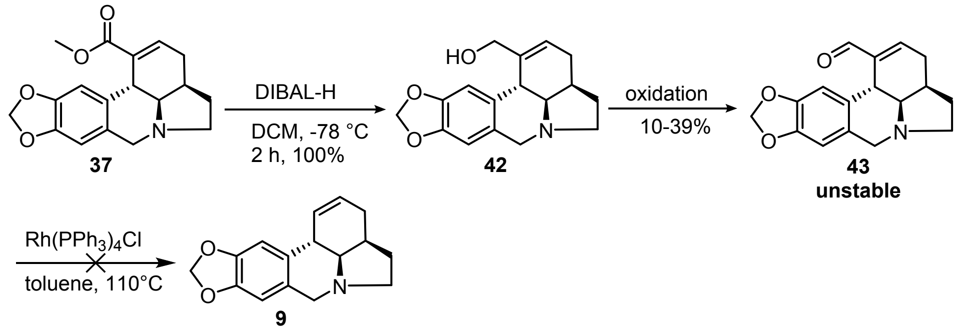

To a stirred solution of **37** (1.02 g, 3.26 mmol) in dry DCM (100 mL) at −78 ºC under N_2 atmosphere was slowly added DIBAL-H (6.51 mL, 6.51 mmol) and the resulting mixture was then stirred for 2 h at the same temperature. The reaction was quenched by addition of H_2O (4 mL) and then extracted with DCM (3 ×40 mL). The combined organic phase was washed with brine (3 × 40 mL), dried over Na_2SO_4, filtered and concentrated under reduced pressure to afford product **42** (928 mg, 100%) as a white solid which was used for the next step without further purification.

Rf = 0.28 (silica, EA : Et_3N = 30 : 1),^{1}H NMR (400 MHz, $CDCl_3$) δ 7.19 (s, 1H), 6.58 (s, 1H), 6.28 – 5.99 (m, 1H), 6.00 – 5.75 (m, 2H), 4.35 (q, *J* = 13.0 Hz, 2H), 3.97 (d, *J* = 14.0 Hz, 1H), 3.80 – 3.59 (m, 1H), 3.49 (d, *J* = 10.1 Hz, 1H), 3.19 – 3.05 (m, 1H), 2.62 – 2.44 (m, 2H), 2.37 (t, *J* = 10.4 Hz, 1H), 2.31 – 2.12 (m, 1H), 2.02 –2.07 (m, 1H), 1.97 – 1.82 (m, 1H), 1.73 – 1.49 (m, 2H). ^{13}C NMR (101 MHz, $CDCl_3$) δ 146.3, 145.8, 142.3, 130.5, 130.1, 130.0, 108.6, 107.7, 101.0, 68.8, 64.0, 56.4, 54.6, 42.8, 37.5, 31.60, 29.9.

The yield of **43** in the next step (**42**→**43**) is low under various oxidation conditions (Table S2), Swern oxidation gave the highest yield of 39%. aldehyde **43** was found to be unstable at room temperature and an attempt to obtain intermediate **9** through a deformylation of **43** was unsuccessful.

Table S2 Optimization of conditions for the oxidation of **42** to **43**[a]

Entry	Solvent	Oxid. reagent	Temp.	Time (h)	Result (yield) [b]
1	DCM	PDC (2.0 equv.)	rt to reflux.	5	15%

2	DCM	PCC (2.0 equv.)	rt to reflux.	8	18%
3	DCM	DMP (1.2 equv.)	0 °C to rt	3	24%
4	DCM	SO_3•py (1.2 equv.)	0 °C to rt	2	30%
5	DCM	MnO_2 (5.0 equv.)	rt to reflux.	6	10%
6	DCM	Swern (1.5 equv.)	-78 °C	1.5	39%

[a] Reactions were conducted on a 0.1 mmol scale using 1.0 equiv. **42**, 5.0 mL DCM. [b] Isolated yields after chromatographic purification.

3. Crude ^{1}H NMR for AD-mix-β mediated asymmetric dihydroxylation

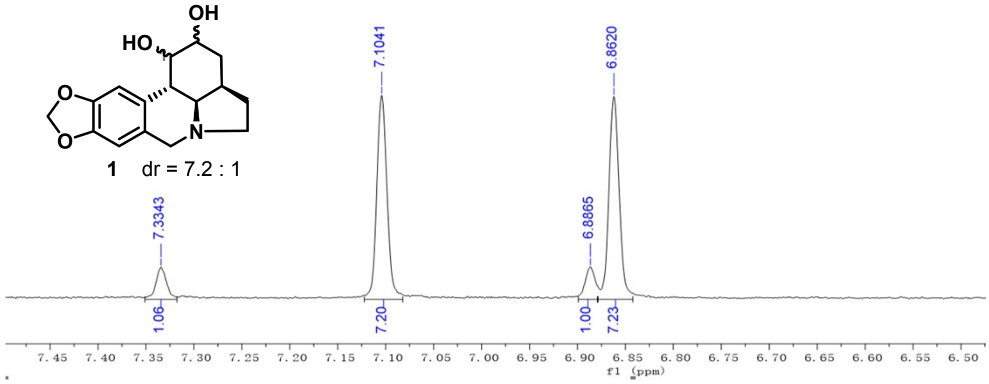

4. Crude ^{1}H NMR of 34

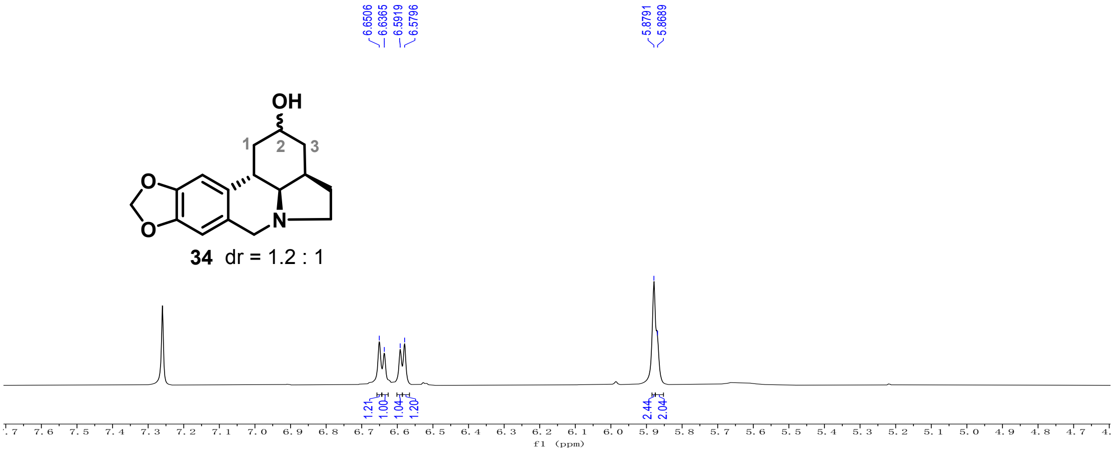

5. Computational details

Geometry optimizations and frequency analyses were performed at the B3LYP/6-31G(d)-PCM-(THF) level of theory using Gaussian 16 package.[16] Single-point energies were calculated at the theoretical level of B3LYP/6-311G(d,p)-PCM-(THF).

1. Enolization Reaction of **10**:

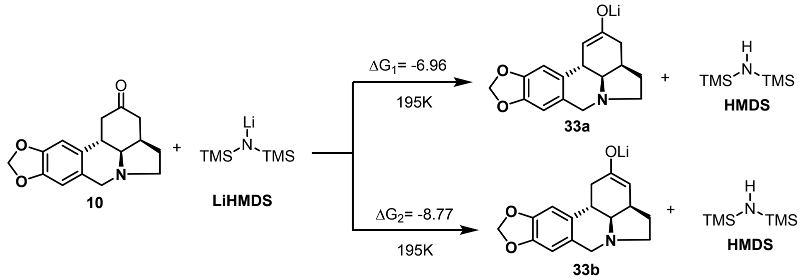

Scheme S1 Thermochemistry calculated for enolization reaction (kcal/mol)

Compound	E_{ele}	G_{corr}(195K)	G(195K)
**10**	-900.563842025	0.283948000	-900.279894025
**LiHMDS**	-881.075816349	0.204396000	-880.871420349
**33a**	-907.549585043	0.272329000	-907.277256043
**33b**	-907.552454108	0.272322000	-907.280132108
**HMDS**	-874.100653283	0.215499000	-873.885154283

Free Energy	calculation	result (Hartree)	result (kcal/mol)
ΔG_1	-0.011096	-0.011096	-6.96285096
ΔG_2	-0.013972	-0.013972	-8.76756972

Owing to complexity of the transition state, thermochemistry of the enolization reaction was investigated by DFT calculations. According to the free energy change, **33b** was more stable than **33a**, but **33a** was exclusively produced, implying that the selectivity for this reaction may be kinetic control process instead of thermodynamic control.

2. Elimination Reaction of **35**:

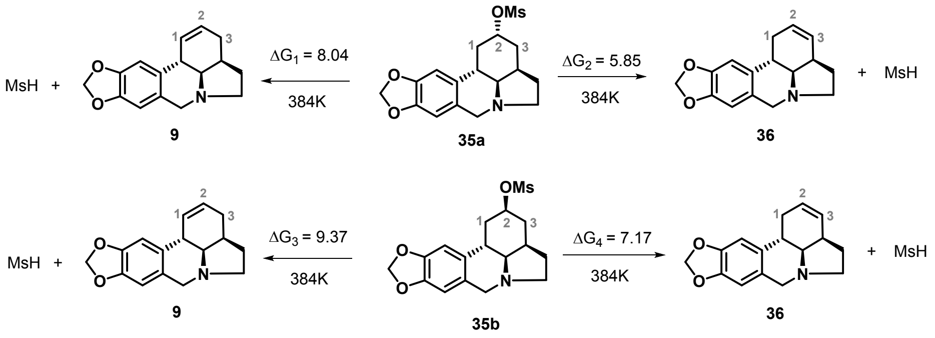

Scheme S2 Thermochemistry calculated for elimination reaction (kcal/mol)

Compound	E_{ele}	G_{corr}(384K)	G(384K)
**35a**	-1414.494276	0.296305	-1414.197971
**35b**	-1414.495885	0.295804	-1414.200081
**36**	-825.2929114	0.245344	-825.0475674
**9**	-825.2896096	0.245536	-825.0440736
**MsH**	-589.1609153	0.019835	-589.1410803

Free Energy	result (Hartree)	result (kcal/mol)
ΔG_1	0.012817	8.042997728
ΔG_2	0.009324	5.850625253
ΔG_3	0.014927	9.36714423
ΔG_4	0.011434	7.174771755

DFT calculations were conducted to shed light on the thermochemistry of elimination reactions (Scheme **S2**). For both **35a** and **35b**, the formation of compound **36** is favorable than formation of **9** according to the free energy change, but all process cannot proceed spontaneously. **35a** is more likely to undergo elimination than **35b** to form compound **36**. The calculated results supported the conclusion that elimination reaction of **36** is a thermodynamic control process.

Cartesian coordinates and energy

10

Charge = 0 Multiplicity = 1

C	2.9286	0.77777	-0.09473
C	3.26689	-0.56101	0.07673
C	2.3038	-1.54374	0.14791
C	0.95028	-1.15578	0.04015
C	0.60482	0.20134	-0.12869
C	1.61653	1.18617	-0.1999
O	4.07059	1.5468	-0.10336
C	5.15987	0.61279	-0.13323
O	4.63331	-0.68509	0.18217
C	-0.86526	0.58191	-0.27866

C	-1.70259	-0.49216	0.41369
C	-3.24246	-0.31379	0.31896
C	-1.30217	1.96342	0.22496
C	-3.62743	0.97711	-0.4279
C	-2.81325	2.17476	0.05509
O	-3.32519	3.25584	0.29215
C	-3.7315	-1.64421	-0.31342
N	-1.41232	-1.76648	-0.23395
C	-0.10287	-2.25711	0.148
C	-2.57575	-2.62069	-0.0297
H	2.57637	-2.58616	0.28416
H	1.37563	2.23521	-0.33149
H	5.90203	0.89549	0.61653
H	5.59887	0.59455	-1.13948
H	-1.10814	0.52863	-1.34996
H	-1.41071	-0.51317	1.48302
H	-3.64062	-0.24062	1.33689
H	-1.0687	2.08799	1.29216
H	-0.8015	2.78677	-0.29657
H	-4.69056	1.21383	-0.32767
H	-3.41907	0.85524	-1.50164
H	-4.68522	-1.98283	0.10185
H	-3.85479	-1.53027	-1.39655
H	-0.10206	-2.64929	1.18805
H	0.1744	-3.09942	-0.49932
H	-2.55714	-3.47908	-0.71089
H	-2.6373	-3.01271	1.00529

Zero-point correction= 0.308116 (Hartree)
Thermal correction to Energy= 0.315020
Thermal correction to Enthalpy= 0.315638
Thermal correction to Gibbs Free Energy= 0.283948
FINAL SINGLE POINT ENERGY -900.563842025

33a

Charge = 0 Multiplicity = 1

C	2.94283	0.87605	-0.11998
C	3.36419	-0.42598	0.1262
C	2.46557	-1.46586	0.23213
C	1.09172	-1.17462	0.08433
C	0.66054	0.14943	-0.15538
C	1.60834	1.19136	-0.26464
O	4.03566	1.71802	-0.15056
C	5.17833	0.85154	-0.13963
O	4.73686	-0.45282	0.26203

C	-0.82597	0.42533	-0.35216
C	-1.59179	-0.67453	0.38579
C	-3.13206	-0.58149	0.27434
C	-1.39605	1.77231	0.0325
C	-3.5639	0.69462	-0.48065
C	-2.74979	1.92248	-0.03137
C	-3.53588	-1.95227	-0.33252
N	-1.21889	-1.95877	-0.19991
C	0.11149	-2.34288	0.22444
C	-2.34004	-2.86368	0.01115
H	2.80407	-2.48007	0.42456
H	1.29366	2.21032	-0.46102
H	5.91092	1.22451	0.57946
H	5.6068	0.79795	-1.15009
H	-1.02275	0.23846	-1.42457
H	-1.30082	-0.627	1.45343
H	-3.54163	-0.5168	1.28942
H	-0.76017	2.59562	0.3495
H	-4.62964	0.89811	-0.3277
H	-3.42011	0.53568	-1.56139
H	-4.48396	-2.33216	0.06211
H	-3.63059	-1.87584	-1.42254
H	0.12063	-2.68784	1.28223
H	0.46164	-3.19231	-0.37793
H	-2.26497	-3.74712	-0.63503
H	-2.40902	-3.22086	1.05955
O	-3.41982	3.01662	0.2451
Li	-4.2495	4.40841	0.8132

Zero-point correction= 0.296859 (Hartree)
Thermal correction to Energy= 0.304316
Thermal correction to Enthalpy= 0.304933
Thermal correction to Gibbs Free Energy= 0.272329
FINAL SINGLE POINT ENERGY -907.549585043

33b

Charge = 0 Multiplicity = 1

C	-2.91639	0.87512	0.06261
C	-3.35409	-0.43937	-0.0569
C	-2.4658	-1.49225	-0.10056
C	-1.08673	-1.20167	-0.01647
C	-0.6376	0.1332	0.10052
C	-1.57635	1.18912	0.14147
O	-4.00124	1.72716	0.04825
C	-5.15071	0.87462	0.14401

O	-4.72945	-0.46487	-0.15028
C	0.85972	0.41613	0.2134
C	1.58886	-0.73357	-0.47385
C	3.13515	-0.67679	-0.44568
C	1.39775	1.75733	-0.29319
C	3.66635	0.68898	-0.09373
C	2.91858	1.82841	-0.09503
C	3.52042	-1.8874	0.45278
N	1.22089	-1.9493	0.24923
C	-0.1153	-2.38222	-0.0819
C	2.35173	-2.86241	0.21822
H	-2.81568	-2.51615	-0.19728
H	-1.25407	2.22085	0.22933
H	-5.89882	1.18902	-0.58692
H	-5.55282	0.91551	1.16569
H	1.13115	0.35489	1.27711
H	1.24528	-0.77333	-1.52696
H	3.4794	-0.9486	-1.45867
H	1.14004	1.91053	-1.35312
H	4.74466	0.78734	0.04156
H	4.49419	-2.31502	0.19063
H	3.54606	-1.59274	1.50827
H	-0.17294	-2.83556	-1.09676
H	-0.4355	-3.16357	0.62125
H	2.25637	-3.6298	0.99653
H	2.4656	-3.38495	-0.75419
O	3.40629	3.03766	0.07915
Li	3.96758	4.63509	0.35667
H	0.9598	2.59841	0.25772

Zero-point correction= 0.296967 (Hartree)
Thermal correction to Energy= 0.304470
Thermal correction to Enthalpy= 0.305088
Thermal correction to Gibbs Free Energy= 0.272322
FINAL SINGLE POINT ENERGY -907.552454108

LiHMDS

Charge = 0 Multiplicity = 1

Si	-2.16901	1.991	-0.01441
Si	-2.17351	-0.68191	-1.5928
C	-1.34007	2.56796	1.6099
H	-1.54784	3.62643	1.8139
H	-0.24623	2.45709	1.57037
H	-1.70227	1.9967	2.47653
C	-4.03683	2.32067	0.21753

H	-4.23299	3.37078	0.47352
H	-4.45336	1.70024	1.02192
H	-4.60315	2.09252	-0.69441
C	-1.58429	3.22046	-1.35559
H	-1.81785	4.2582	-1.08119
H	-2.06269	3.02023	-2.32287
H	-0.4993	3.15517	-1.51051
C	-1.42354	-2.41318	-1.28063
H	-1.66897	-3.10832	-2.09428
H	-1.80304	-2.85902	-0.35038
H	-0.32695	-2.37608	-1.21042
C	-4.04897	-0.96634	-1.82428
H	-4.25123	-1.69289	-2.623
H	-4.56865	-0.03596	-2.08694
H	-4.51145	-1.34793	-0.90457
C	-1.51465	-0.13691	-3.3023
H	-1.75756	-0.87055	-4.08304
H	-0.4233	-0.01686	-3.28903
H	-1.94275	0.82559	-3.61035
Li	-0.60595	-0.38334	1.01423
N	-1.75981	0.3605	-0.30705

Zero-point correction= 0.228164 (Hartree)
Thermal correction to Energy= 0.236069
Thermal correction to Enthalpy= 0.236687
Thermal correction to Gibbs Free Energy= 0.204396
FINAL SINGLE POINT ENERGY -881.075816349

HMDS

Charge = 0 Multiplicity = 1

N	-3.61279	0.40754	-0.2807
Si	-4.09992	2.04243	0.1333
Si	-3.90673	-0.63883	-1.65976
C	-2.86861	2.69097	1.41517
H	-3.14445	3.69873	1.74937
H	-1.85283	2.74102	1.00522
H	-2.83861	2.04854	2.30443
C	-5.84194	2.09832	0.8768
H	-6.13061	3.12398	1.14079
H	-5.90417	1.48957	1.78736
H	-6.58869	1.71416	0.17121
C	-4.07065	3.13989	-1.40749
H	-4.37412	4.16415	-1.15671
H	-4.75621	2.77947	-2.18412
H	-3.06624	3.18525	-1.84467

C	-3.5484	-2.41099	-1.10248
H	-3.68065	-3.1161	-1.93231
H	-4.21924	-2.72015	-0.29201
H	-2.51724	-2.51879	-0.7429
C	-5.70531	-0.49149	-2.22939
H	-5.89195	-1.1384	-3.09592
H	-5.95941	0.5329	-2.5281
H	-6.3993	-0.78755	-1.43383
C	-2.77956	-0.2159	-3.12277
H	-2.96397	-0.88485	-3.97335
H	-1.72177	-0.30926	-2.84757
H	-2.94153	0.81231	-3.46837
H	-3.05445	-0.0377	0.44385

Zero-point correction= 0.238822 (Hartree)
Thermal correction to Energy= 0.246667
Thermal correction to Enthalpy= 0.247285
Thermal correction to Gibbs Free Energy= 0.215499
FINAL SINGLE POINT ENERGY -874.100653283

35a

Charge = 0 Multiplicity = 1

C	-3.45006	-1.2885	-0.1667
C	-4.18408	-0.16333	0.1954
C	-3.58638	1.06882	0.34394
C	-2.19598	1.16382	0.11738
C	-1.4495	0.0233	-0.24417
C	-2.0923	-1.22652	-0.39275
O	-4.27937	-2.38566	-0.21892
C	-5.60738	-1.86899	-0.06098
O	-5.50197	-0.50937	0.38458
C	0.04148	0.17831	-0.52813
C	0.54682	1.38166	0.26311
C	2.04863	1.72383	0.07883
C	0.96486	-1.01183	-0.22238
C	2.72004	0.80148	-0.95028
C	2.38209	-0.70492	-0.78011
C	2.03902	3.23196	-0.29857
N	-0.18746	2.55989	-0.17751
C	-1.55118	2.5342	0.31712
C	0.64457	3.71255	0.14351
H	-4.16727	1.9414	0.62794
H	-1.54237	-2.11695	-0.67554
H	-6.13943	-2.45707	0.69086
H	-6.12891	-1.8976	-1.0274

H	0.14534	0.42346	-1.59506
H	0.35694	1.17682	1.33744
H	2.54903	1.60738	1.04644
H	0.98576	-1.17135	0.86338
H	0.61444	-1.94809	-0.66128
H	3.80671	0.93776	-0.96386
H	2.37391	1.08789	-1.95029
H	2.84889	3.79303	0.17709
H	2.13945	3.35226	-1.38327
H	-1.59209	2.78902	1.39859
H	-2.1414	3.30031	-0.20271
H	0.30505	4.60538	-0.39375
H	0.63581	3.94835	1.22717
H	2.48394	-1.1778	-1.76441
S	3.67918	-1.65108	0.11262
O	3.2075	-3.04673	0.2127
O	4.97111	-1.36631	-0.54413
C	3.7775	-0.98838	1.79407
H	4.11411	0.04864	1.7611
H	2.81154	-1.07803	2.29347
H	4.51894	-1.60701	2.30529

Zero-point correction= 0.366147 (Hartree)
Thermal correction to Energy= 0.397889
Thermal correction to Enthalpy= 0.399105
Thermal correction to Gibbs Free Energy= 0.296305
FINAL SINGLE POINT ENERGY -1414.494276

35b

Charge = 0 Multiplicity = 1

C	-3.27294	-1.35147	0.0468
C	-4.09578	-0.23524	-0.0651
C	-3.57537	1.03819	-0.13555
C	-2.1719	1.18489	-0.09055
C	-1.33614	0.05509	0.02944
C	-1.90052	-1.23914	0.0947
O	-4.04463	-2.48921	0.12534
C	-5.38431	-2.05964	-0.14779
O	-5.41486	-0.62804	-0.06183
C	0.17674	0.25114	0.03138
C	0.46653	1.65759	0.54589
C	1.96403	2.05691	0.57024
C	1.02186	-0.74538	0.83669
C	2.85192	0.93462	0.01073
C	2.54502	-0.41453	0.70076

C	2.00444	3.39433	-0.22003
N	-0.18304	2.61116	-0.34353
C	-1.61932	2.60809	-0.14509
C	0.53927	3.86873	-0.20772
H	-4.2254	1.90387	-0.22276
H	-1.28026	-2.1244	0.17754
H	-6.06126	-2.48242	0.59859
H	-5.66955	-2.37112	-1.16214
H	0.51357	0.20837	-1.01322
H	0.05837	1.73315	1.57505
H	2.25941	2.24313	1.60957
H	0.74945	-0.68982	1.89721
H	0.83565	-1.77922	0.5336
H	3.90867	1.19335	0.14506
H	2.68241	0.83324	-1.06652
H	2.68885	4.1258	0.22022
H	2.32066	3.2157	-1.25393
H	-1.89869	3.13285	0.7947
H	-2.10116	3.16104	-0.96226
H	0.29957	4.55185	-1.03062
H	0.30395	4.39046	0.74244
H	3.01326	-0.44863	1.69043
S	3.35268	-1.80034	-0.20379
O	3.06359	-3.04814	0.53141
O	2.99253	-1.70755	-1.6338
C	5.12878	-1.49191	-0.05387
H	5.39088	-0.56105	-0.55866
H	5.40467	-1.46308	1.00208
H	5.61845	-2.33577	-0.54552

Zero-point correction= 0.366179 (Hartree)
Thermal correction to Energy= 0.397953
Thermal correction to Enthalpy= 0.399170
Thermal correction to Gibbs Free Energy= 0.295804
FINAL SINGLE POINT ENERGY -1414.495885

36

Charge = 0 Multiplicity = 1

C	2.80159	0.69059	-0.0803
C	3.01906	-0.67397	0.07628
C	1.9719	-1.56758	0.13418
C	0.65935	-1.06028	0.02553
C	0.43479	0.32561	-0.1297
C	1.5313	1.21525	-0.18415
O	4.00878	1.35562	-0.07415

C	5.00689	0.32801	-0.11855
O	4.37004	-0.91991	0.18594
C	-0.9959	0.84052	-0.26717
C	-1.90077	-0.15126	0.46252
C	-3.41859	0.14726	0.42546
C	-1.30743	2.27776	0.17392
C	-3.72297	1.57374	0.04906
C	-2.79143	2.53541	-0.00266
C	-4.00298	-1.00095	-0.4411
N	-1.74155	-1.43017	-0.22095
C	-0.48948	-2.06722	0.10973
C	-3.00535	-2.14501	-0.1764
H	2.15106	-2.63138	0.26089
H	1.38451	2.28316	-0.30131
H	5.77582	0.53529	0.62971
H	5.43999	0.28124	-1.1276
H	-1.27614	0.77834	-1.32824
H	-1.56851	-0.20753	1.51778
H	-3.8097	-0.00467	1.44304
H	-1.00295	2.4446	1.21915
H	-0.74273	3.00293	-0.42457
H	-4.77053	1.83064	-0.10776
H	-5.03158	-1.25569	-0.16657
H	-3.98349	-0.73734	-1.50423
H	-0.49915	-2.51252	1.12923
H	-0.30544	-2.89675	-0.58678
H	-3.03632	-2.93203	-0.93937
H	-3.20798	-2.62103	0.8052
H	-3.09651	3.56086	-0.20607

Zero-point correction= 0.303642 (Hartree)
Thermal correction to Energy= 0.327254
Thermal correction to Enthalpy= 0.328470
Thermal correction to Gibbs Free Energy= 0.245344
FINAL SINGLE POINT ENERGY -825.2929114

9

Charge = 0 Multiplicity = 1

C	-2.82074	0.69723	0.07637
C	-3.0301	-0.66986	-0.07288
C	-1.97842	-1.55816	-0.13032
C	-0.66757	-1.0427	-0.03554
C	-0.45397	0.34536	0.10387
C	-1.55305	1.23003	0.16886
O	-4.03235	1.35339	0.08078

C	-5.02182	0.31831	0.1452
O	-4.38055	-0.92458	-0.16964
C	0.972	0.8608	0.24362
C	1.90512	-0.14582	-0.44132
C	3.41435	0.19112	-0.37133
C	1.33069	2.25096	-0.22948
C	3.66059	1.57973	0.26928
C	2.62854	2.58452	-0.20895
C	4.03066	-1.04226	0.33937
N	1.74317	-1.422	0.24242
C	0.48604	-2.04534	-0.11514
C	2.99234	-2.15421	0.08915
H	-2.15291	-2.62405	-0.2446
H	-1.40956	2.29784	0.29338
H	-5.80618	0.51864	-0.58849
H	-5.43477	0.26965	1.16264
H	1.22713	0.80733	1.31528
H	1.59769	-0.2169	-1.50242
H	3.79621	0.23319	-1.3974
H	0.57018	2.94347	-0.58196
H	4.67761	1.92645	0.05612
H	3.59137	1.48159	1.36461
H	5.0256	-1.29874	-0.03783
H	4.11333	-0.86183	1.41773
H	0.51606	-2.47086	-1.14228
H	0.28895	-2.88735	0.56195
H	3.05712	-2.97908	0.80878
H	3.1154	-2.58389	-0.92613
H	2.95978	3.56978	-0.52996

Zero-point correction= 0.303570 (Hartree)
Thermal correction to Energy= 0.327118
Thermal correction to Enthalpy= 0.328334
Thermal correction to Gibbs Free Energy= 0.245536
FINAL SINGLE POINT ENERGY -825.2896096

MsH

Charge = 0 Multiplicity = 1

S	-0.28349	-0.0141	0.
H	-1.35905	0.83266	0.
O	-0.28349	-0.73469	1.27937
O	-0.28349	-0.73469	-1.27937
C	1.06966	1.16712	0.
H	1.00896	1.77985	-0.90182
H	1.00896	1.77985	0.90182

H 1.99486 0.58552 0.
Zero-point correction= 0.057131 (Hartree)
Thermal correction to Energy= 0.064031
Thermal correction to Enthalpy= 0.065247
Thermal correction to Gibbs Free Energy= 0.019835
FINAL SINGLE POINT ENERGY -589.1609153

6. References

[1] (a) J. H. Sahner, H. Sucipto, S. C. Wenzel, M. Groh, R. W. Hartmann, R. Müller, *Chem. Bio. Chem*., 2015, **16**, 946; (b) R. K. Boeckman, A. J. Thomas, *J. Org. Chem.,* 1982, **14**, 2823; (c) D. Petrović, R. Brückner, *Org. Lett.,* 2011, **13**, 6524.

[2] (a) R. K. Jr. Boeckman, P. Shao, S. T. Wrobleski, D. J. Boehmler, G. R.Heintzelman, A. J. Barbosa, *J. Am. Chem. Soc.,* 2006, **128**, 10572; (b) D. Petrović, R. Brückner, *Org. Lett.,* 2011, **13**, 6524; (c) Q. Xiao, K. Young, A. Zakarian, *Org. Lett.,* 2013, **15**, 3314; (d) J. B. Chen, J. C. Chen, Y. Xie, H. B. Zhang, *Angew. Chem. Int. Ed.* 2012, **51**, 1024;

[3] B. M. Trost, W. M. Seganish, C. K. Chung, D. Amans, *Chem. Eur. J.,* 2012, **18**, 2948.

[4] (a) A. E. Gatland, B. S. Pilgrim, P. A. Procopiou, T. J. Donohoe, *Angew. Chem. Int. Ed.* 2014, **53**, 14555; (b) Y. Wu, H. Zhang, Y. Zhao, J. Zhao, J. Chen, L. Li, *Org. Lett.,* 2007, **9**, 1199; (c) P. Bałczewski, M. Koprowski, A. Bodzioch, B. Marciniak, E. Różycka-Sokołowska, *J. Org. Chem.,* 2006, **71**, 2899; (d) B. J. Arnold, S. M. Mellows, P. G. Sammes, *J. Chem. Soc. Perkin Trans. 1,* 1973, 1266; (e) G. R. Geen, I. S. Mann, V. Mullane, A. McKillop, *Tetrahedron.,* 1988, **54**, 9875.

[5] C. J. Moody, G. J. Warrellow, *Tetrahedron Lett.,* 1987, **28**, 6089.

[6] (a) A. P. Kozikowski, C. S. Li, *J. Org. Chem.,* 1985, **50**, 778; (b) L. E. Blidi, M. Ahbala, J. Boltea, M. Lemaire, *Tetrahedron,* 2006, **17**, 2684; c) T. Yasuhara, K. Nishimura, M. Yamashita, N. Fukuyama, K. Yamada, O. Muraoka, K. Tomioka, *Org. Lett.,* 2003, **5**, 71123; d) N. Kise, S. Isemoto, T. Sakurai, *J. Org. Chem.,* 2011, **76**, 9856.

[7] (a) D. A. Evans, D. Seidel, *J. Am. Chem. Soc.,* 2005, **127**, 9958; (b) D. A. Evans, S. Mito, D. Seidel, *J. Am. Chem. Soc.,* 2007, **129**, 11583.

[8] D. L. Comins, A. Dehghani, *Tetrahedron Lett.,* 1992, **33**, 6299.

[9] (a) S. G. Hentges, K. B. Sharpless, *J. Am. Chem. Soc.,* 1980, **102**, 4263; (b) E. N. Jacobsen, I. Marko, W. S. Mungall, G. Schroeder, K. B. Sharpless, *J. Am. Chem. Soc.,* 1988, **110**, 1968.

[10] M. R. Herreraa, A. K. Machochoa, J. J. Nair, W. E. Campbell, R. Brun, F. Viladomat, C. Codina, J. Bastida, *Fitoterapia,* 2001, **72**, 444.

[11] Y. J. Chen, S. L. Cai, C. C. Wang, J. D. Cheng, S. Kramer, X. W. Sun, *Chem. Asian. J.,* 2017, **12**, 1309.

[12] K. Ishii, Y. Seki-Yoritate, M. Ishibashi, M. W. Liaw, T. Oishi, T. Sato, N. Chida, *Heterocycles,* 2019, **99**, 111.

[13] W. J. Huang, O. V. Singh, C. H. Chen, S. Y. Chiou, S. S. Lee, *Helv. Chim. Acta.,* 2002, **85**, 1069.

[14] J. R. Dice and G. R. Allen, *J. Am. Chem. Soc.,* 1952, **74** , 1231.

[15] J. Tsuji, K. Ohno, *Tetrahedron Lett.*, 1967, **23**, 2173.

[16] Frisch, M. J.; Trucks, G. W.; Schlegel, H. B.; Scuseria, G. E.; Robb, M. A.; Cheeseman, J. R.;

Scalmani, G.; Barone, V.; Petersson, G. A.; Nakatsuji, H.; Li, X.; Caricato, M.; Marenich, A. V.; Bloino, J.; Janesko, B. G.; Gomperts, R.; Mennucci, B.; Hratchian, H. P.; Ortiz, J. V.; Izmaylov, A. F.; Sonnenberg, J. L.; Williams; Ding, F.; Lipparini, F.; Egidi, F.; Goings, J.; Peng, B.; Petrone, A.; Henderson, T.; Ranasinghe, D.; Zakrzewski, V. G.; Gao, J.; Rega, N.; Zheng, G.; Liang, W.; Hada, M.; Ehara, M.; Toyota, K.; Fukuda, R.; Hasegawa, J.; Ishida, M.; Nakajima, T.; Honda, Y.; Kitao, O.; Nakai, H.; Vreven, T.; Throssell, K.; Montgomery Jr., J. A.; Peralta, J. E.; Ogliaro, F.; Bearpark, M. J.; Heyd, J. J.; Brothers, E. N.; Kudin, K. N.; Staroverov, V. N.; Keith, T. A.; Kobayashi, R.; Normand, J.; Raghavachari, K.; Rendell, A. P.; Burant, J. C.; Iyengar, S. S.; Tomasi, J.; Cossi, M.; Millam, J. M.; Klene, M.; Adamo, C.; Cammi, R.; Ochterski, J. W.; Martin, R. L.; Morokuma, K.; Farkas, O.; Foresman, J. B.; Fox, D. J. *Gaussian 16 Rev. A.03*, Wallingford, CT, 2016.

7. ^{1}H and ^{13}C NMR spectra for all compounds

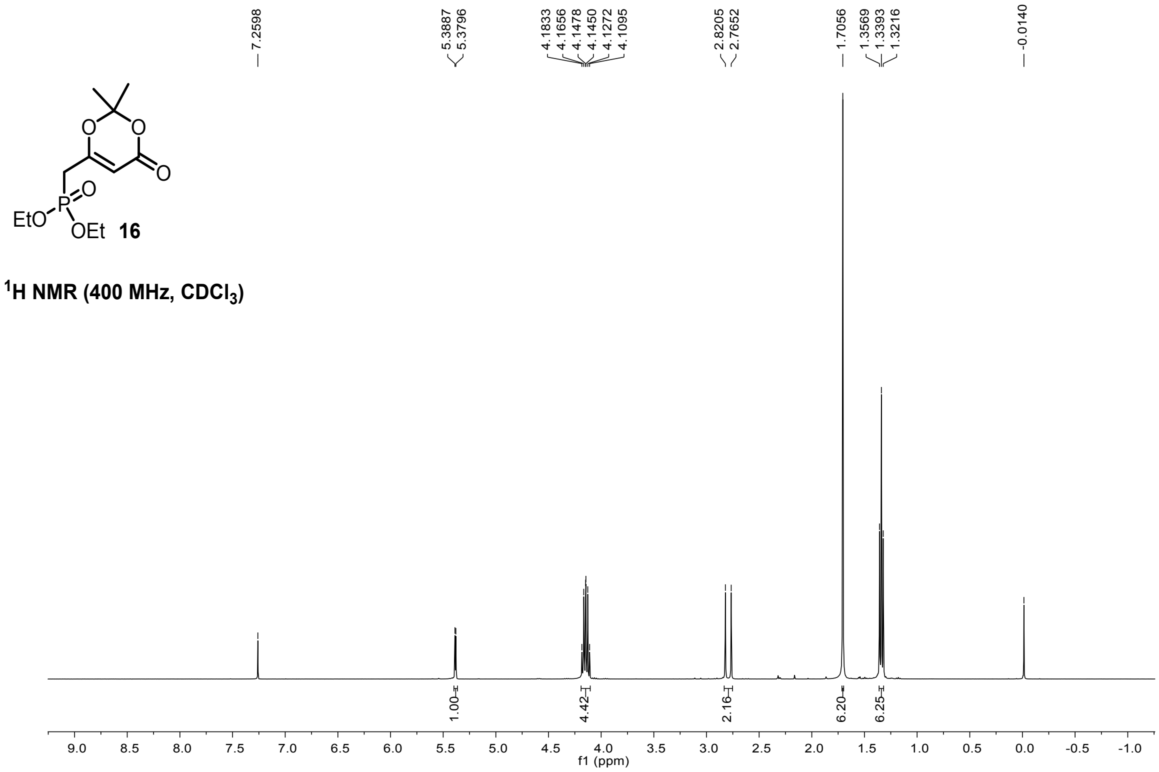

16

^{13}C NMR (100 MHz, CDCl$_3$)

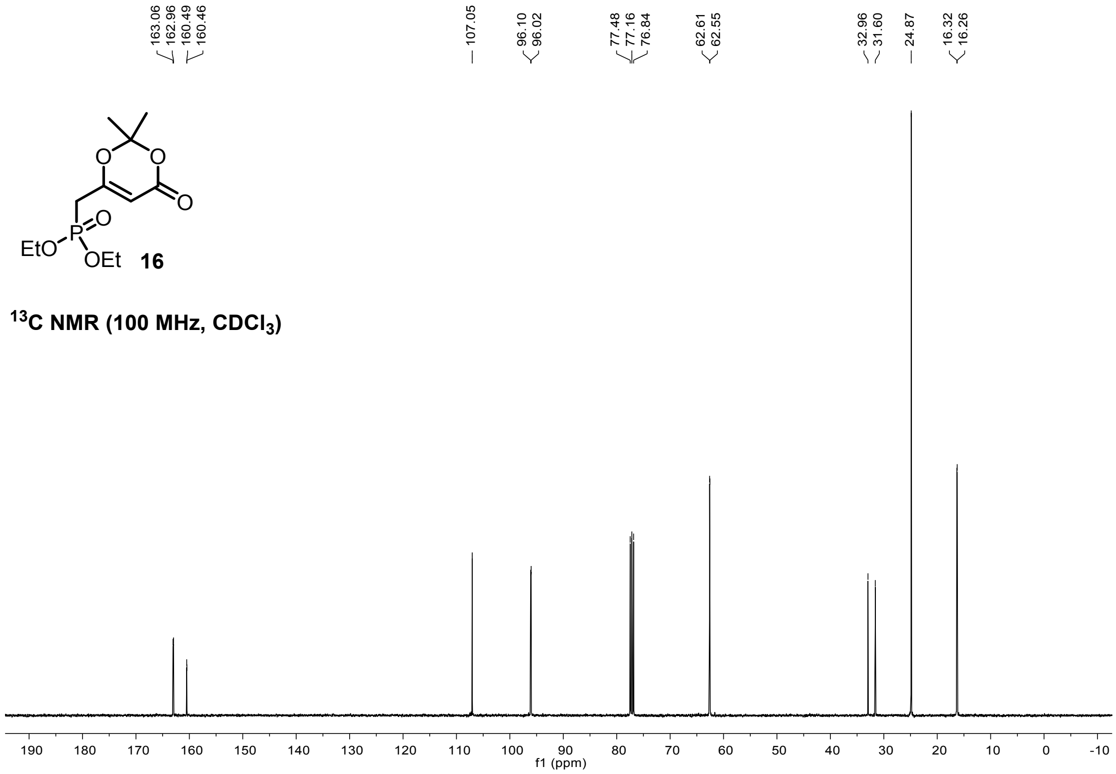

17

^{1}H NMR (400 MHz, CDCl$_3$)

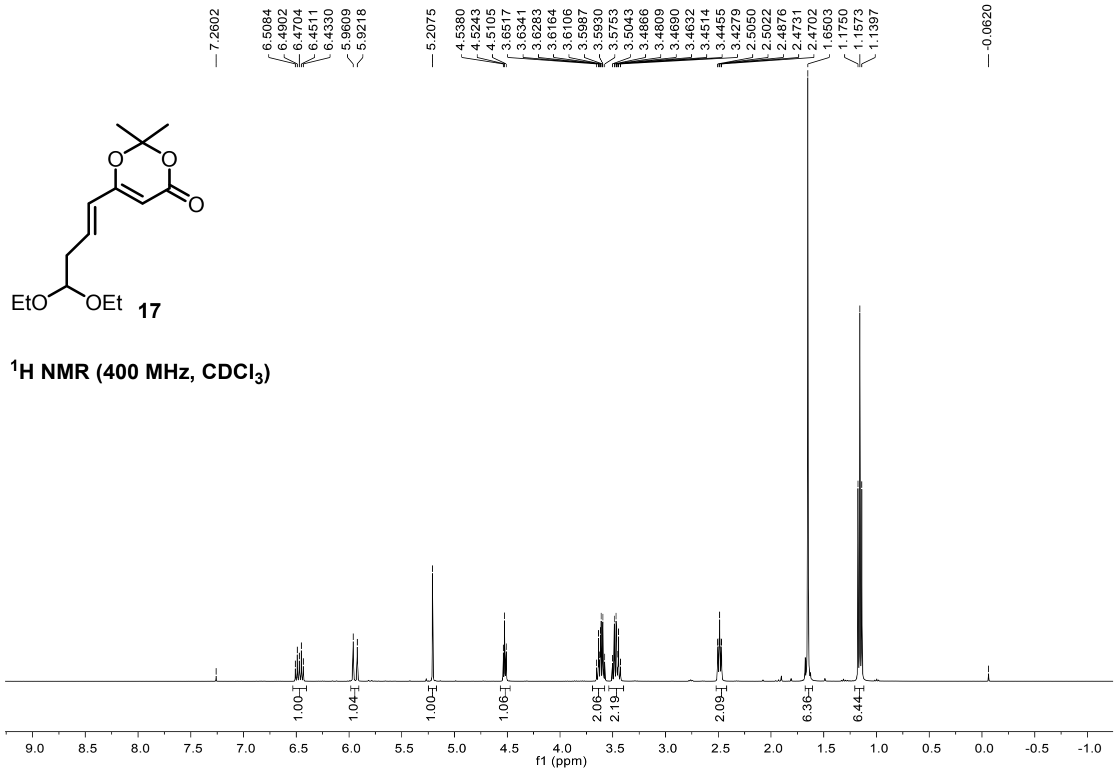

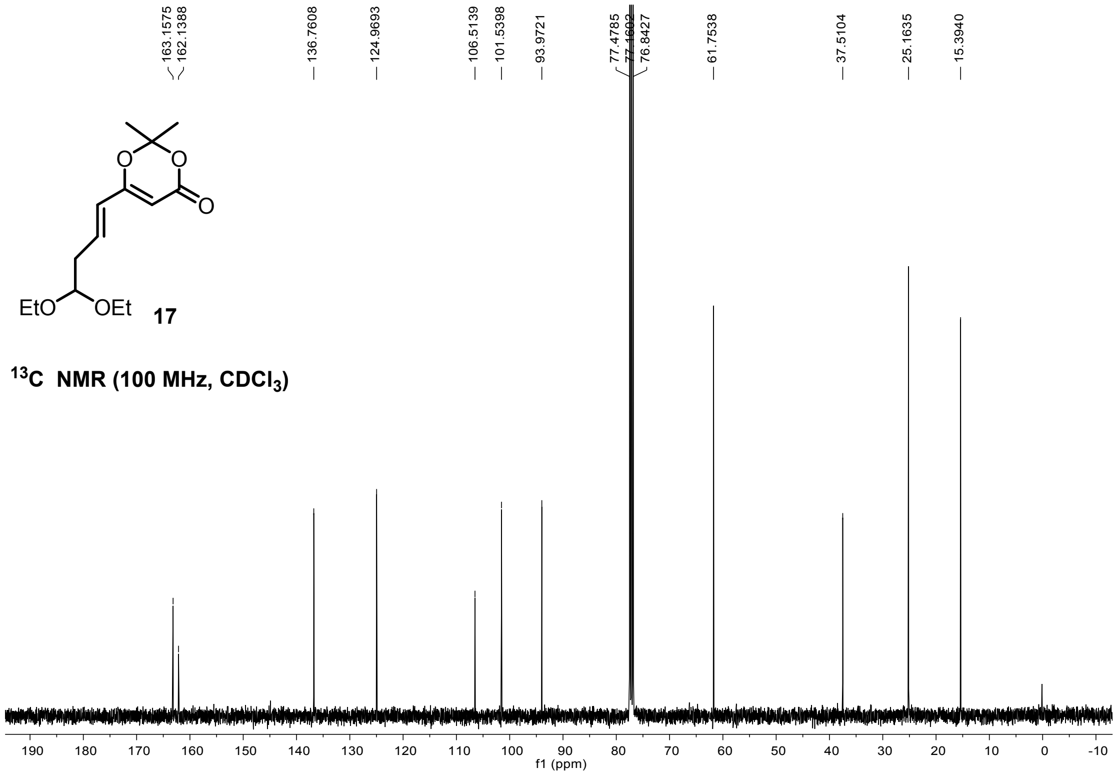
163.1575
162.1388
136.7608
124.9693
106.5139
101.5398
93.9721
77.4785
77.1602
76.8427
61.7538
37.5104
25.1635
15.3940
EtO
OEt
O
O
O
17
13C NMR (100 MHz, CDCl3)
190 180 170 160 150 140 130 120 110 100 90 80 70 60 50 40 30 20 10 0 -10
f1 (ppm)

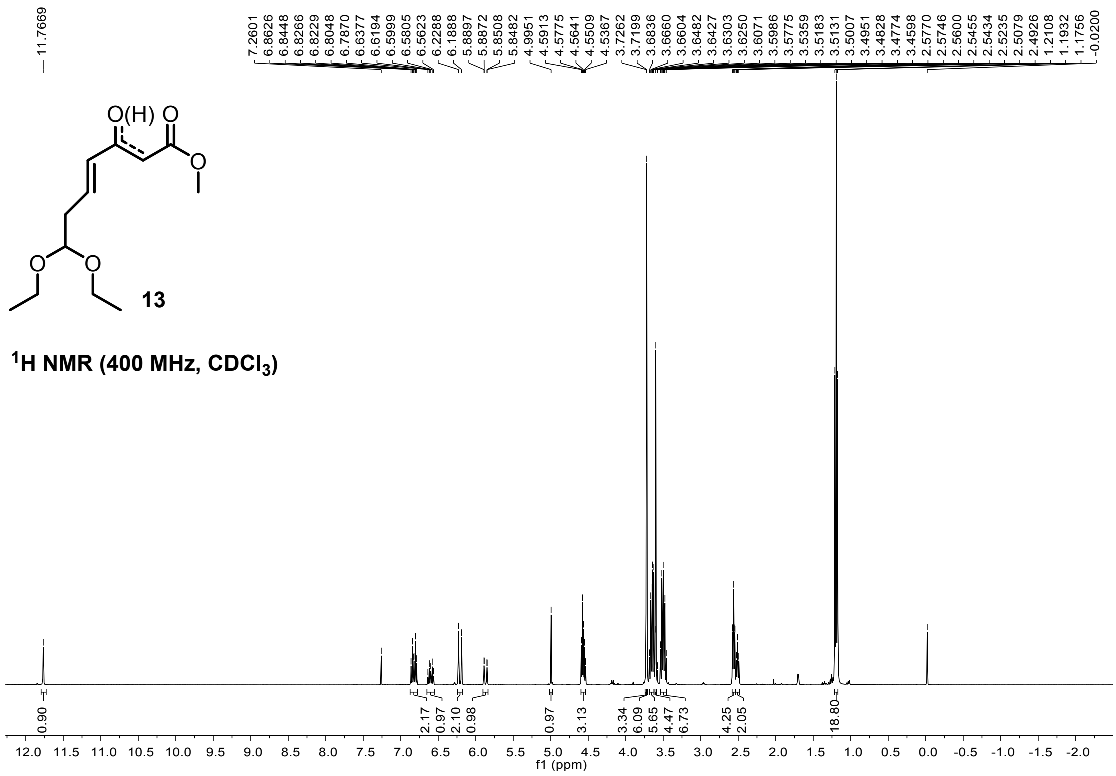
11.7669
7.2601
6.8626
6.8448
6.8266
6.8229
6.8048
6.7870
6.6377
6.6194
6.5999
6.5805
6.5623
6.2288
6.1888
5.8897
5.8872
5.8508
5.8482
4.9951
4.5913
4.5775
4.5641
4.5509
4.5367
3.7262
3.7199
3.6836
3.6660
3.6604
3.6482
3.6427
3.6303
3.6250
3.6071
3.5986
3.5775
3.5359
3.5183
3.5131
3.5007
3.4951
3.4828
3.4774
3.4598
2.5770
2.5746
2.5600
2.5455
2.5434
2.5235
2.5079
2.4926
1.2108
1.1932
1.1756
-0.0200
O(H)
O
O
O
O
13
1H NMR (400 MHz, CDCl3)
0.90
2.17
0.97
2.10
0.98
0.97
3.13
3.34
6.09
5.65
4.47
6.73
4.25
2.05
18.80
12.0 11.5 11.0 10.5 10.0 9.5 9.0 8.5 8.0 7.5 7.0 6.5 6.0 5.5 5.0 4.5 4.0 3.5 3.0 2.5 2.0 1.5 1.0 0.5 0.0 -0.5 -1.0 -1.5 -2.0
f1 (ppm)

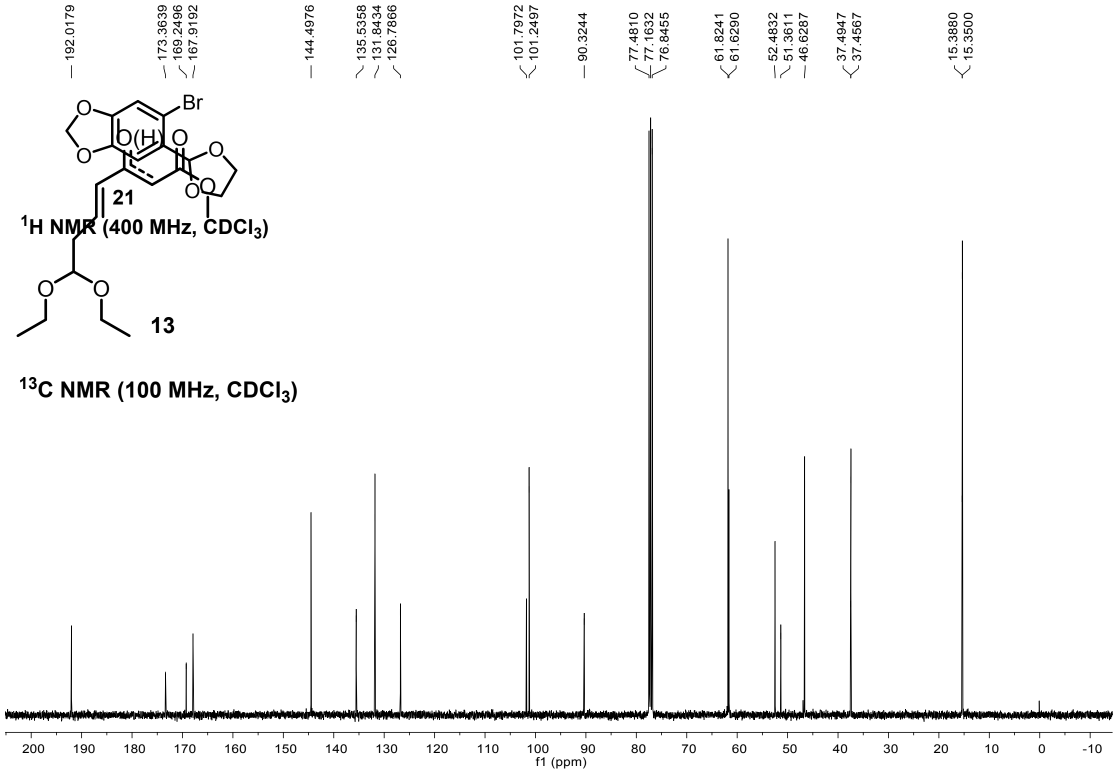
192.0179
173.3639
169.2496
167.9192
144.4976
135.5358
131.8434
126.7866
101.7972
101.2497
90.3244
77.4810
77.1632
76.8455
61.8241
61.6290
52.4832
51.3611
46.6287
37.4947
37.4567
15.3880
15.3500
Br
21
^{1}H NMR (400 MHz, $CDCl_3$)
13
^{13}C NMR (100 MHz, $CDCl_3$)
200 190 180 170 160 150 140 130 120 110 100 90 80 70 60 50 40 30 20 10 0 -10
f1 (ppm)

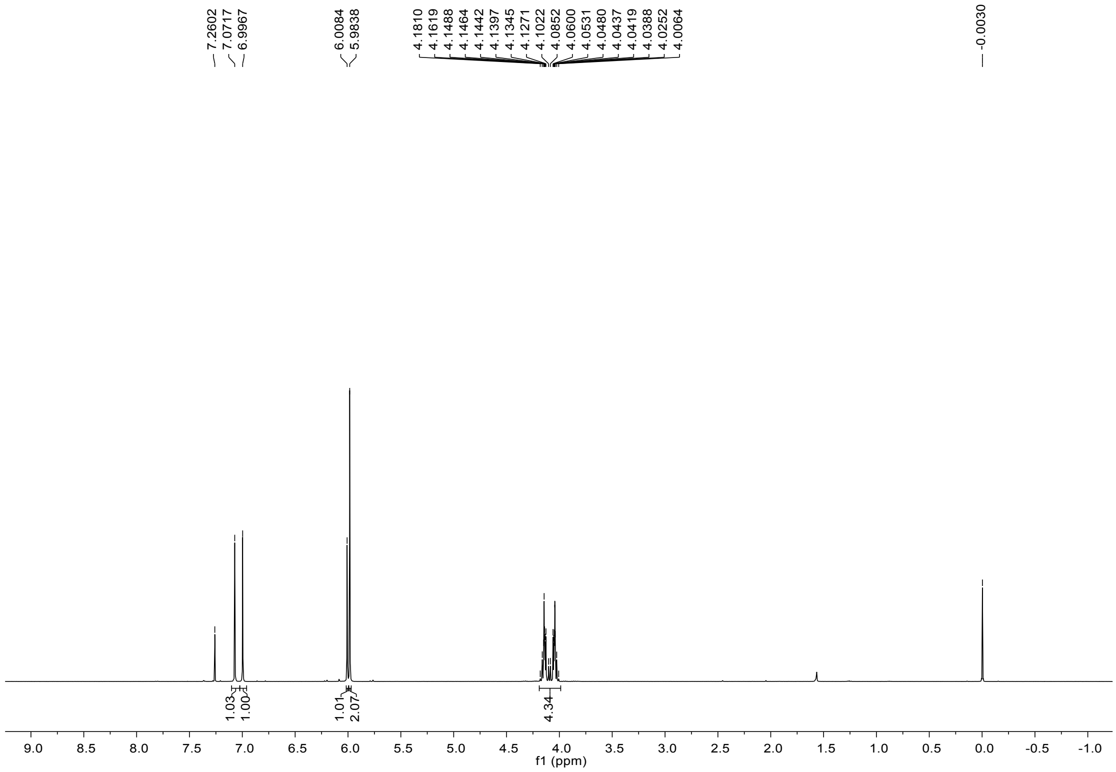
7.2602
7.0717
6.9967
6.0084
5.9838
4.1810
4.1619
4.1488
4.1464
4.1442
4.1397
4.1345
4.1271
4.1022
4.0852
4.0600
4.0531
4.0480
4.0437
4.0419
4.0388
4.0252
4.0064
-0.0030
1.03
1.00
1.01
2.07
4.34
9.0 8.5 8.0 7.5 7.0 6.5 6.0 5.5 5.0 4.5 4.0 3.5 3.0 2.5 2.0 1.5 1.0 0.5 0.0 -0.5 -1.0
f1 (ppm)

21

^{13}C NMR (100 MHz, CDCl$_3$)

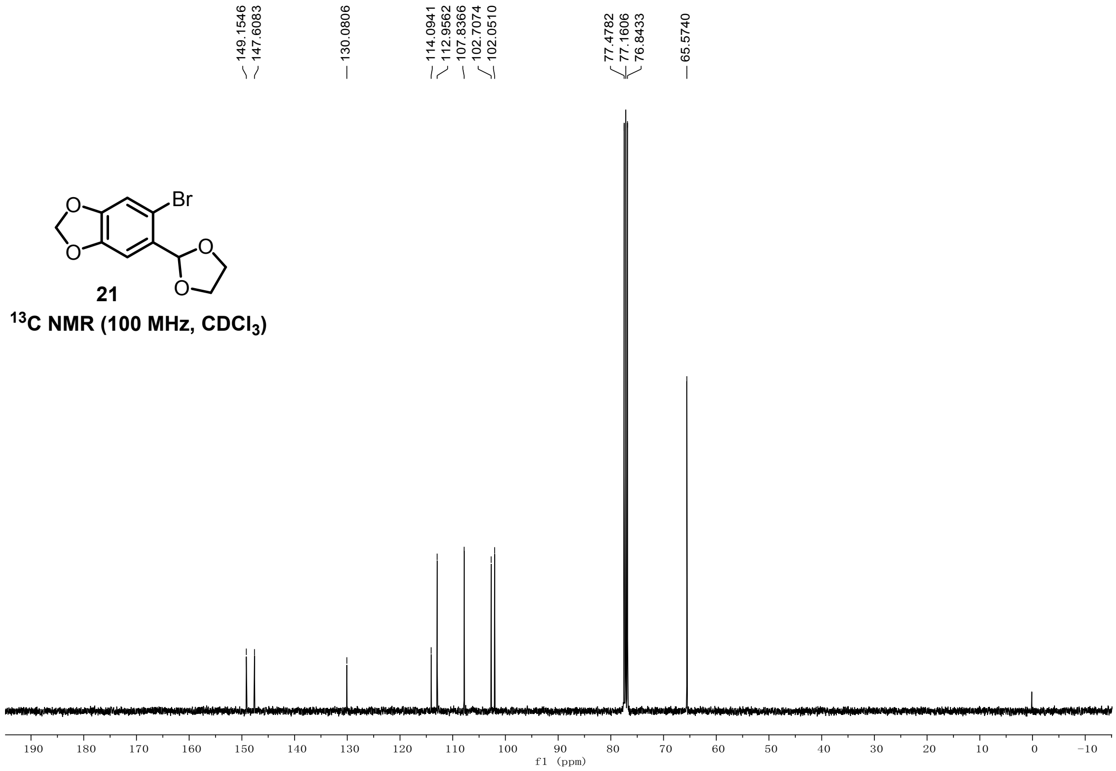

22

^{1}H NMR (400 MHz, CDCl$_3$)

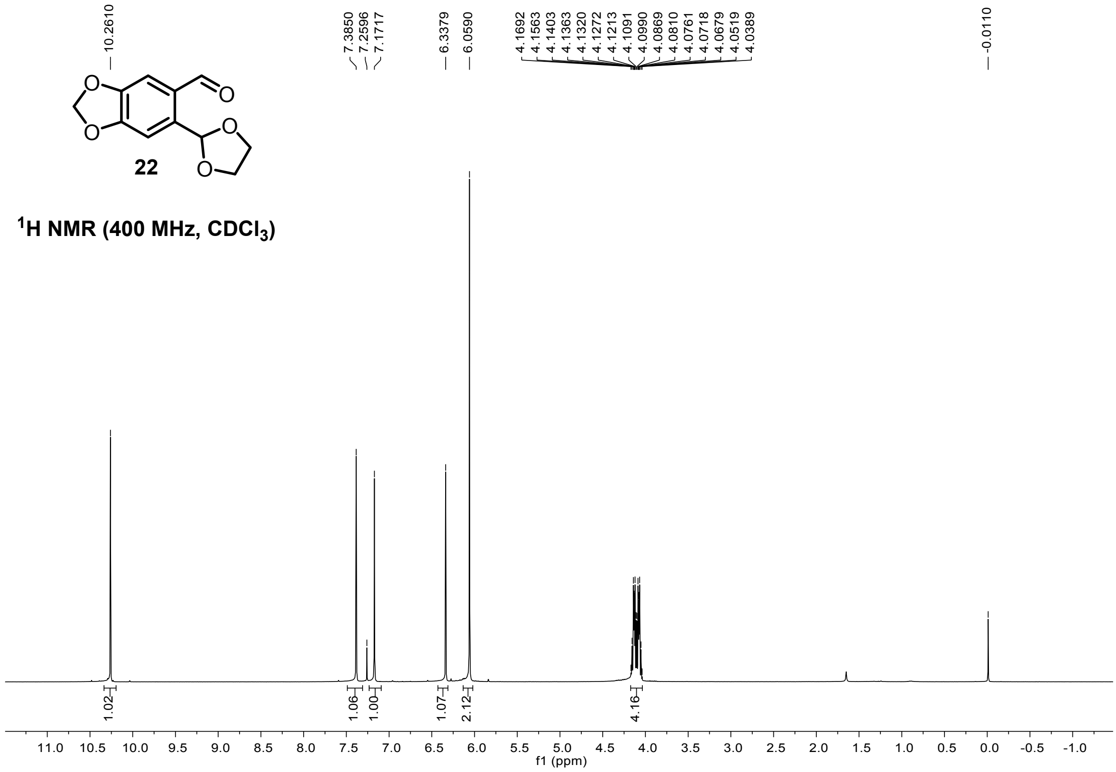

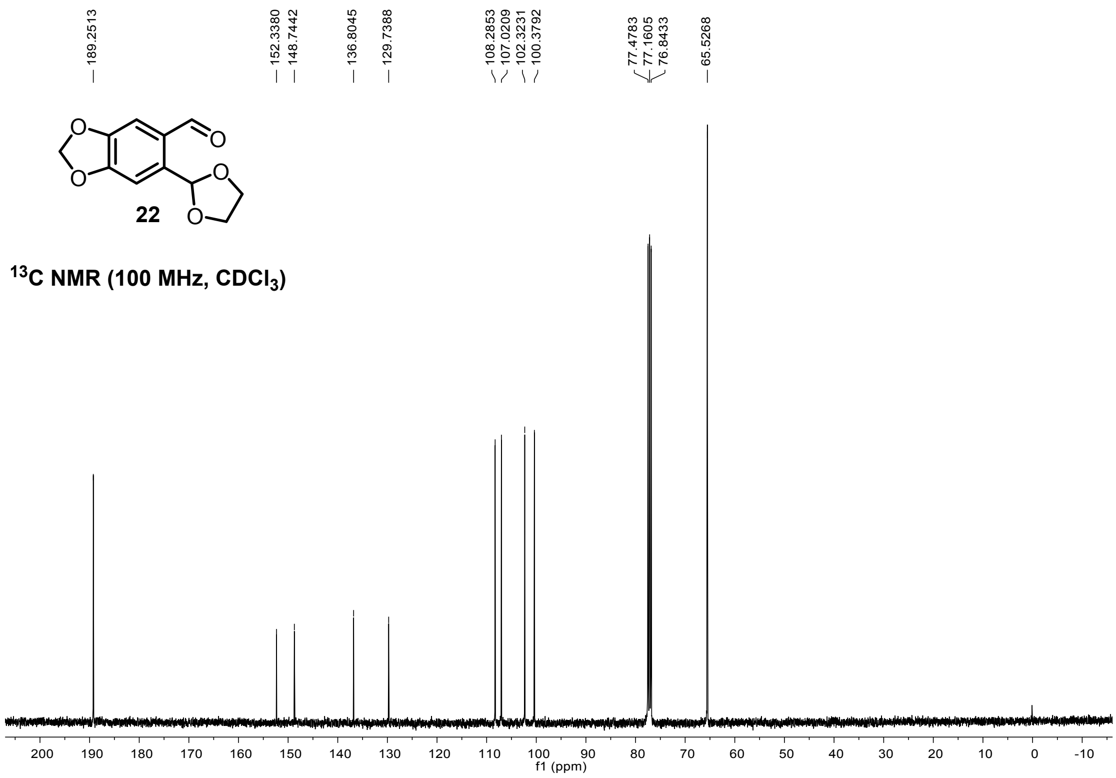
189.2513
152.3380
148.7442
136.8045
129.7388
108.2853
107.0209
102.3231
100.3792
77.4783
77.1605
76.8433
65.5268
O
O
O
O
O
22
13C NMR (100 MHz, CDCl3)
200 190 180 170 160 150 140 130 120 110 100 90 80 70 60 50 40 30 20 10 0 -10
f1 (ppm)

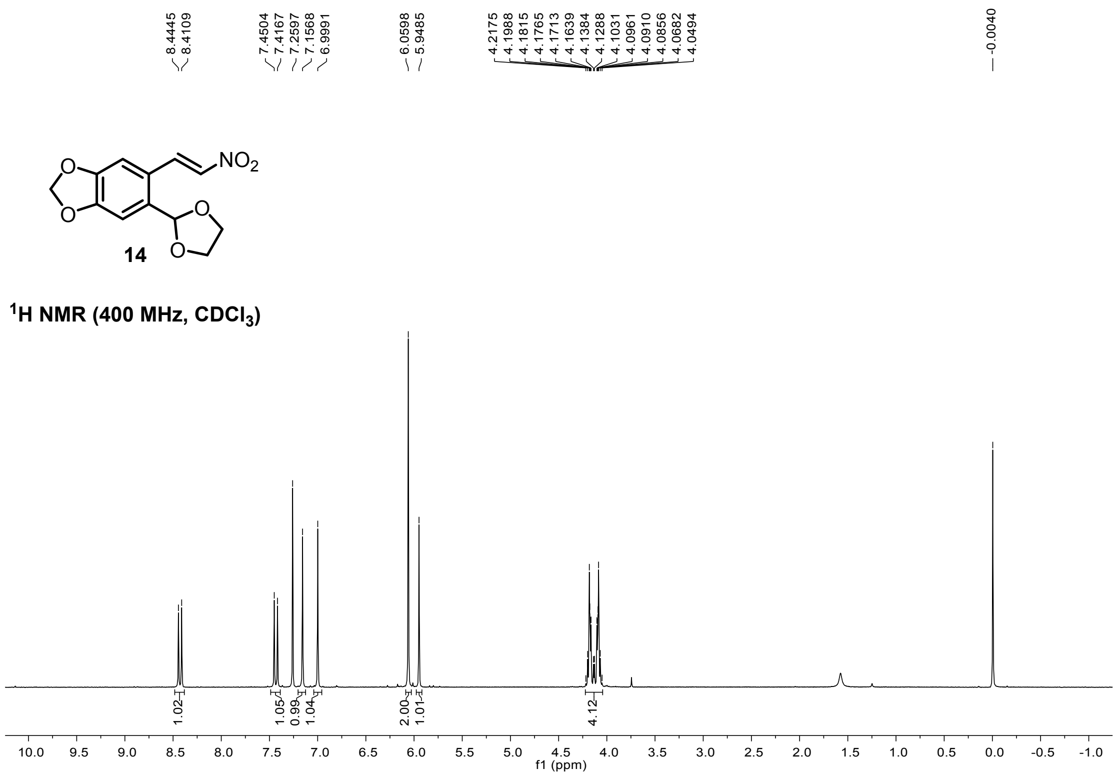
8.4445
8.4109
7.4504
7.4167
7.2597
7.1568
6.9991
6.0598
5.9485
4.2175
4.1988
4.1815
4.1765
4.1713
4.1639
4.1384
4.1288
4.1031
4.0961
4.0910
4.0856
4.0682
4.0494
-0.0040
NO2
O
O
O
O
14
1H NMR (400 MHz, CDCl3)
1.02
1.05
0.99
1.04
2.00
1.01
4.12
10.0 9.5 9.0 8.5 8.0 7.5 7.0 6.5 6.0 5.5 5.0 4.5 4.0 3.5 3.0 2.5 2.0 1.5 1.0 0.5 0.0 -0.5 -1.0
f1 (ppm)

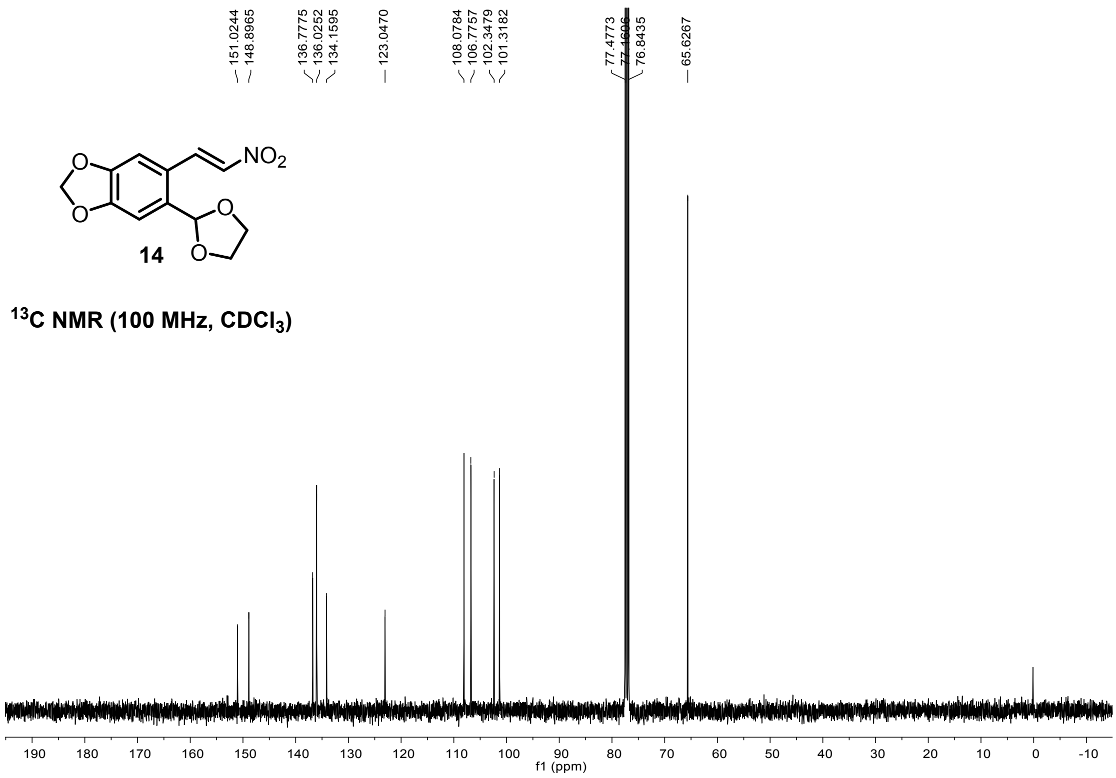
151.0244
148.8965
136.7775
136.0252
134.1595
123.0470
108.0784
106.7757
102.3479
101.3182
77.4773
76.8435
65.6267
NO_2
14
^{13}C NMR (100 MHz, $CDCl_3$)
f1 (ppm)

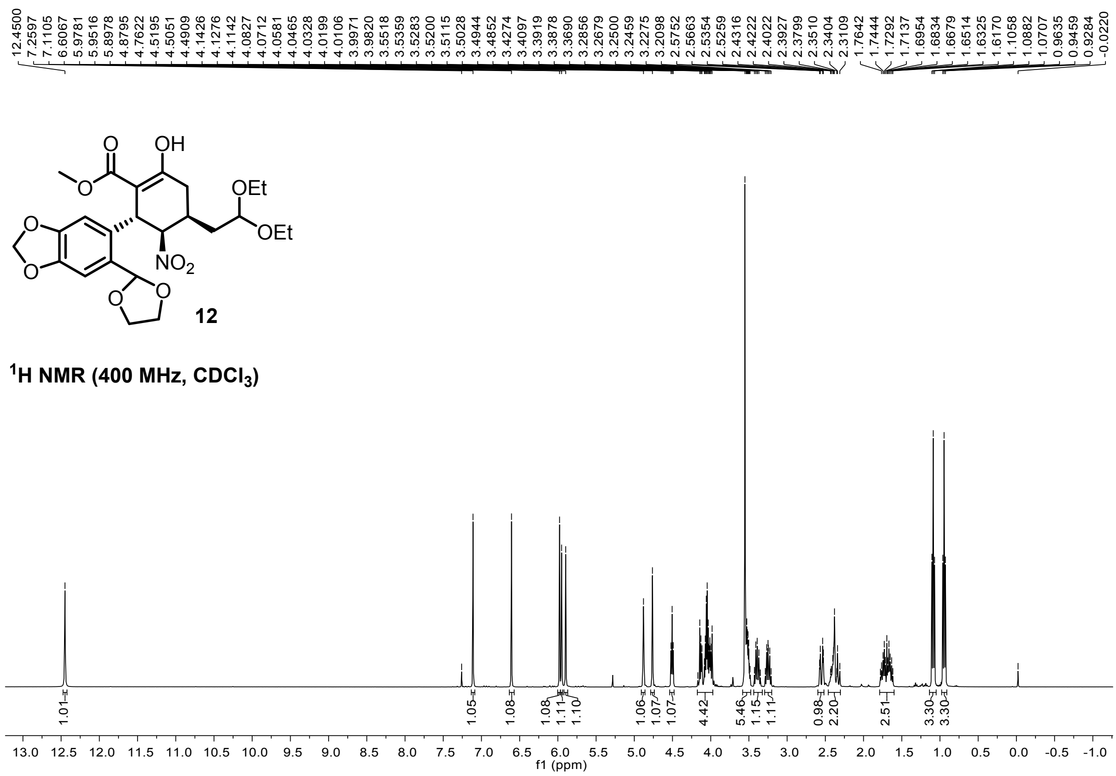
12.4500
7.2597
7.1105
6.6067
5.9781
5.9516
5.8978
4.8795
4.7622
4.5195
4.5051
4.4909
4.1426
4.1276
4.1142
4.0827
4.0712
4.0581
4.0465
4.0328
4.0199
4.0106
3.9971
3.9820
3.5518
3.5359
3.5283
3.5200
3.5115
3.5028
3.4944
3.4852
3.4274
3.4097
3.3919
3.3878
3.3690
3.2856
3.2679
3.2500
3.2459
3.2275
3.2098
2.5752
2.5663
2.5354
2.5259
2.4316
2.4222
2.4022
2.3927
2.3799
2.3510
2.3404
2.3109
1.7642
1.7444
1.7292
1.7137
1.6954
1.6834
1.6679
1.6514
1.6325
1.6170
1.1058
1.0882
1.0707
0.9635
0.9459
0.9284
-0.0220
OH
OEt
OEt
NO_2
12
^{1}H NMR (400 MHz, $CDCl_3$)
f1 (ppm)

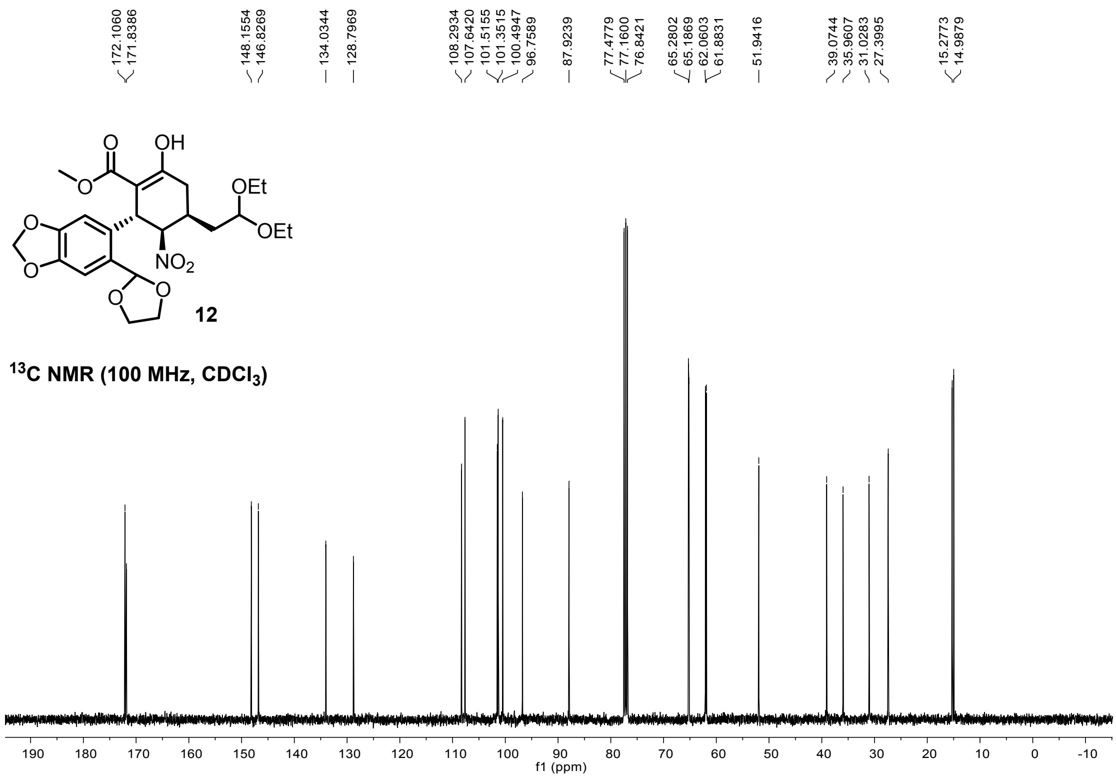
172.1060
171.8386
148.1554
146.8269
134.0344
128.7969
108.2934
107.6420
101.5155
101.3515
100.4947
96.7589
87.9239
77.4779
77.1600
76.8421
65.2802
65.1869
62.0603
61.8831
51.9416
39.0744
35.9607
31.0283
27.3995
15.2773
14.9879
OH
OEt
OEt
NO2
12
13C NMR (100 MHz, CDCl3)
f1 (ppm)

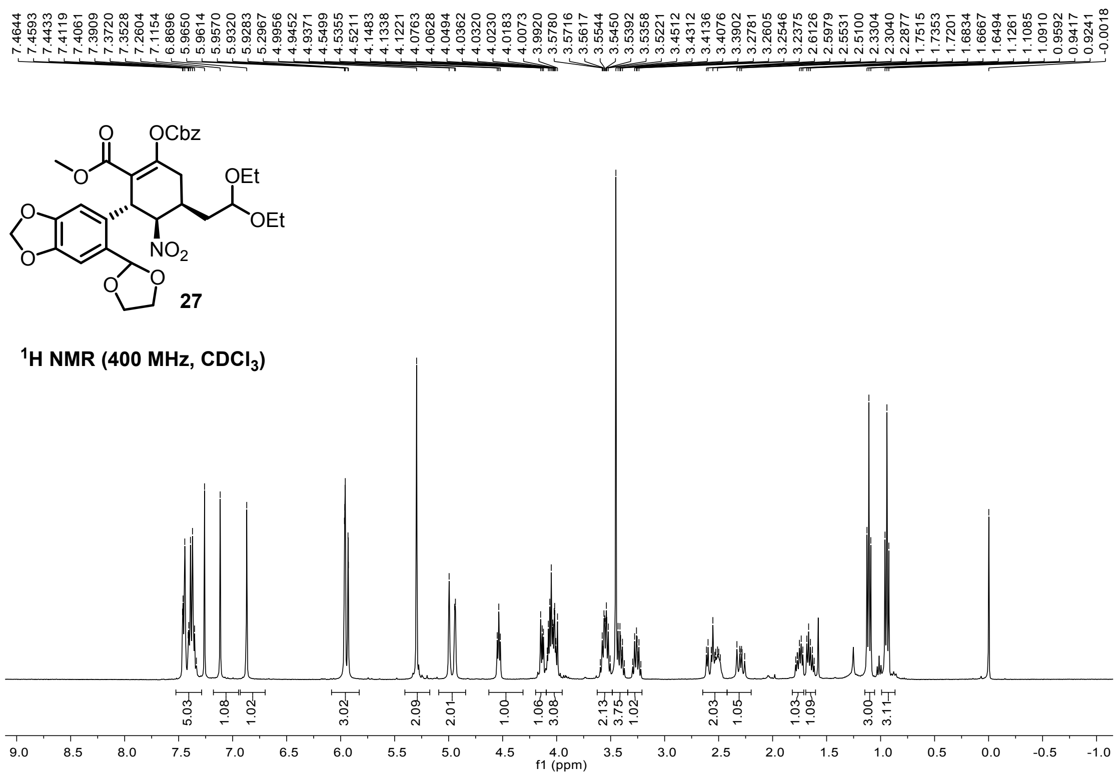
OCbz
OEt
OEt
NO2
27
1H NMR (400 MHz, CDCl3)
f1 (ppm)

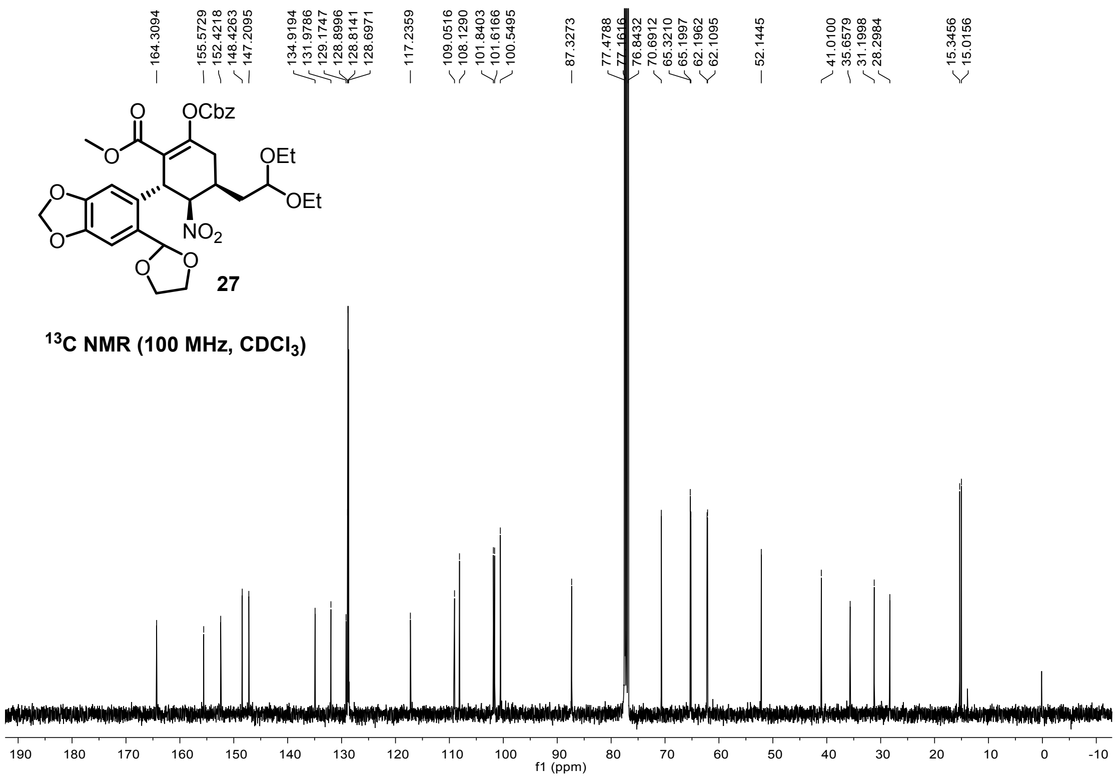
OCbz
OEt
OEt
NO2
27
13C NMR (100 MHz, CDCl3)
f1 (ppm)

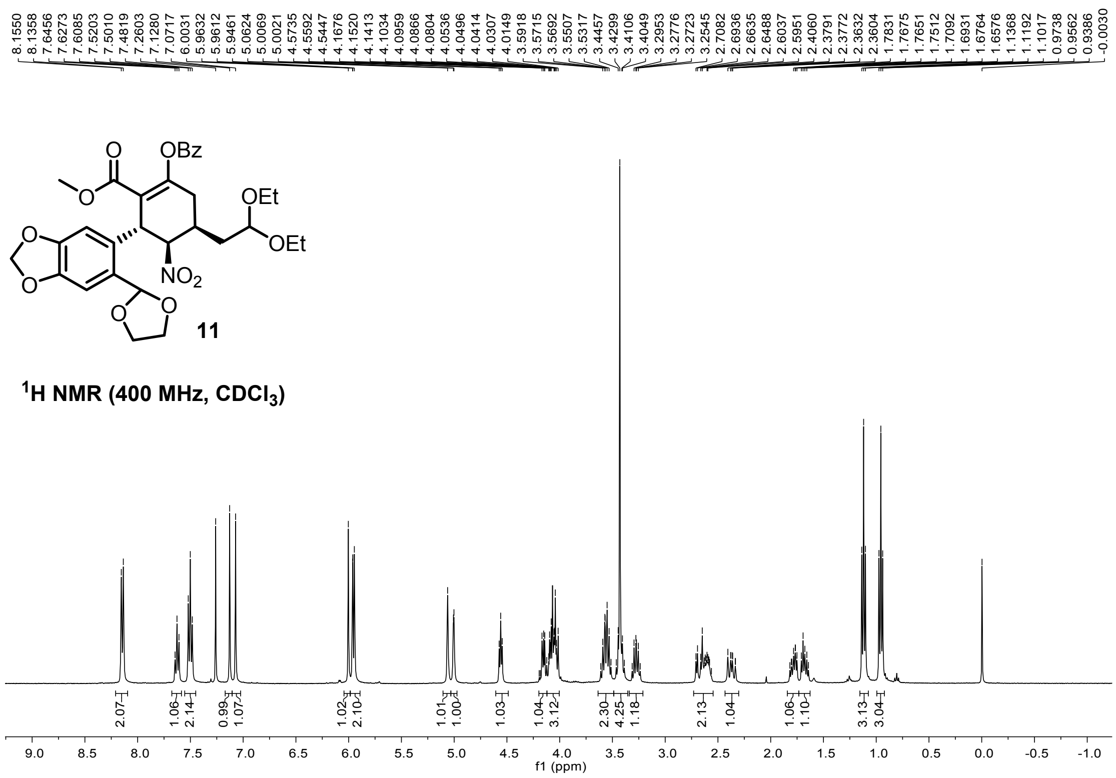
OBz
OEt
OEt
NO2
11
1H NMR (400 MHz, CDCl3)
f1 (ppm)

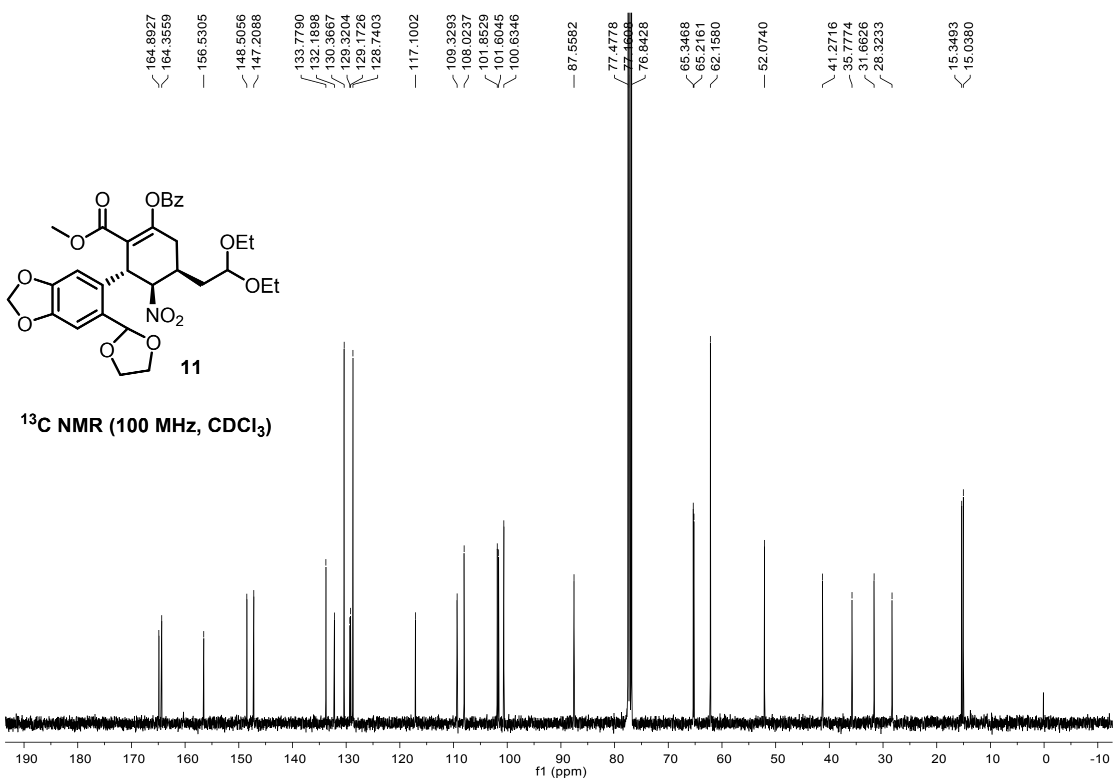
164.8927
164.3559
156.5305
148.5056
147.2088
133.7790
132.1898
130.3667
129.3204
129.1726
128.7403
117.1002
109.3293
108.0237
101.8529
101.6045
100.6346
87.5582
77.4778
76.8428
65.3468
65.2161
62.1580
52.0740
41.2716
35.7774
31.6626
28.3233
15.3493
15.0380
OBz
O
O
OEt
OEt
O
O
NO2
O
O
11
13C NMR (100 MHz, CDCl3)
190
180
170
160
150
140
130
120
110
100
90
80
70
60
50
40
30
20
10
0
-10
f1 (ppm)

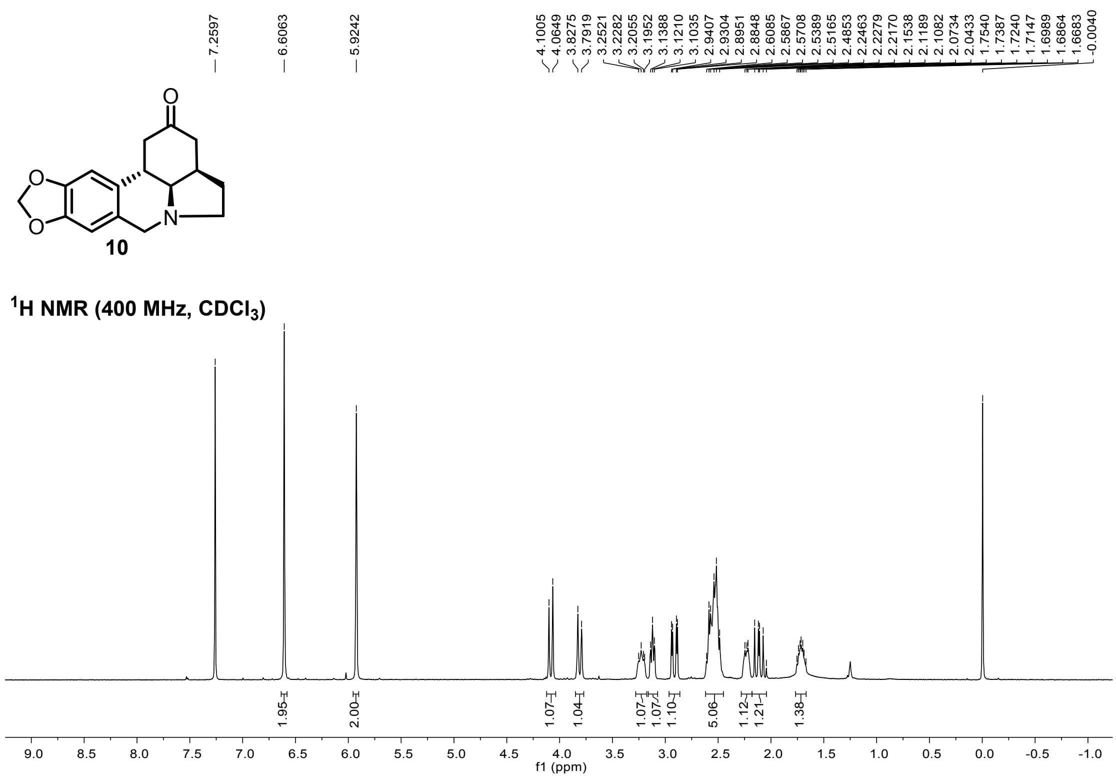
7.2597
6.6063
5.9242
4.1005
4.0649
3.8275
3.7919
3.2521
3.2282
3.2055
3.1952
3.1388
3.1210
3.1035
2.9407
2.9304
2.8951
2.8848
2.6085
2.5867
2.5708
2.5389
2.5165
2.4853
2.2463
2.2279
2.2170
2.1538
2.1189
2.1082
2.0734
2.0433
1.7540
1.7387
1.7240
1.7147
1.6989
1.6864
1.6683
-0.0040
O
O
O
N
10
1H NMR (400 MHz, CDCl3)
1.95
2.00
1.07
1.04
1.07
1.07
1.10
5.06
1.12
1.21
1.38
9.0 8.5 8.0 7.5 7.0 6.5 6.0 5.5 5.0 4.5 4.0 3.5 3.0 2.5 2.0 1.5 1.0 0.5 0.0 -0.5 -1.0
f1 (ppm)

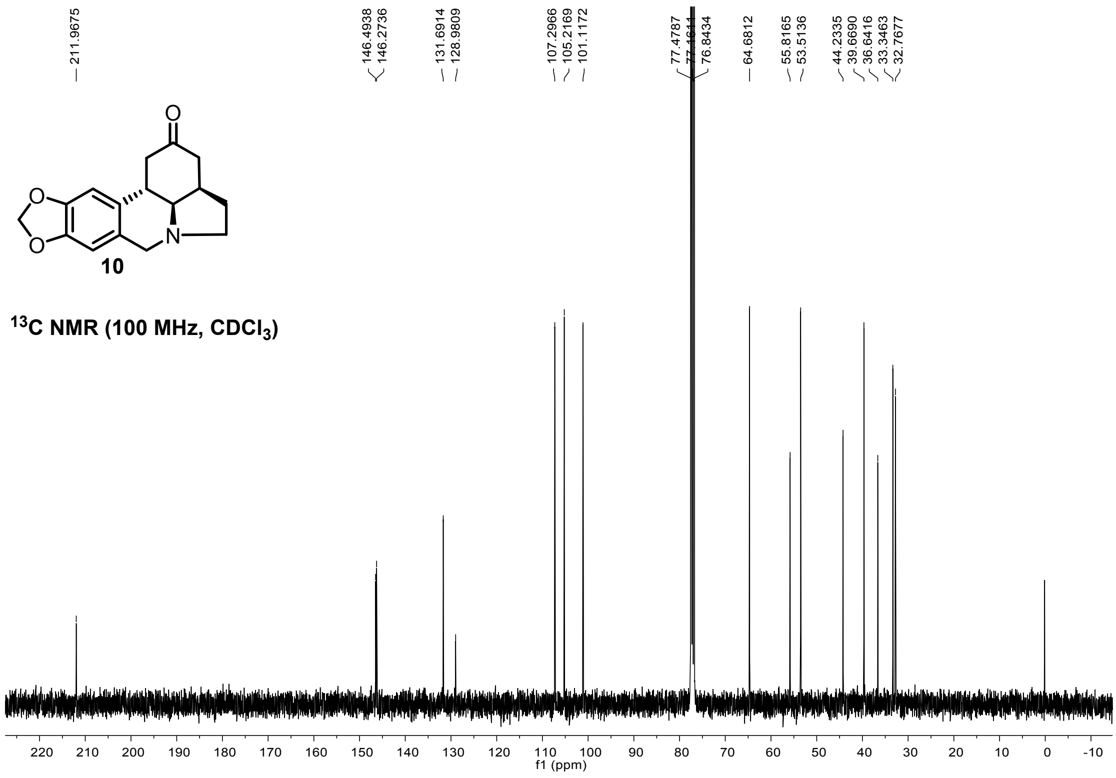
211.9675
146.4938
146.2736
131.6914
128.9809
107.2966
105.2169
101.1172
77.4787
77.1641
76.8434
64.6812
55.8165
53.5136
44.2335
39.6690
36.6416
33.3463
32.7677
O
O
O
N
10
13C NMR (100 MHz, CDCl3)
220 210 200 190 180 170 160 150 140 130 120 110 100 90 80 70 60 50 40 30 20 10 0 -10
f1 (ppm)

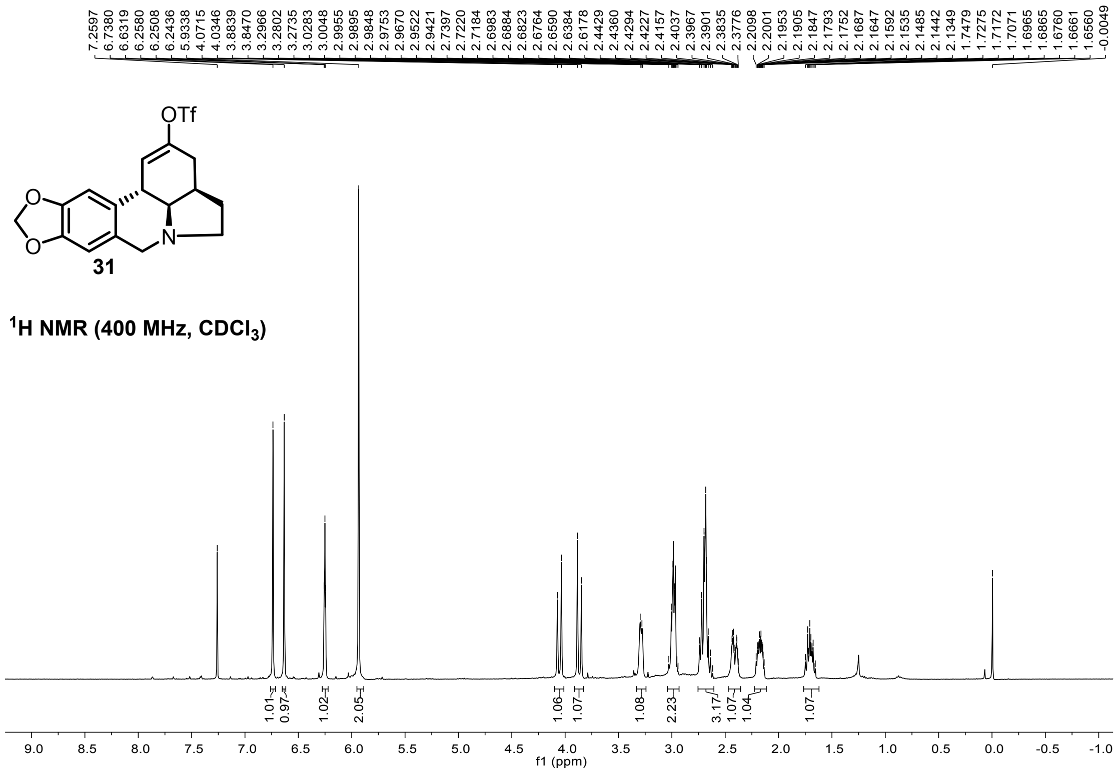

31

^{1}H NMR (400 MHz, $CDCl_3$)

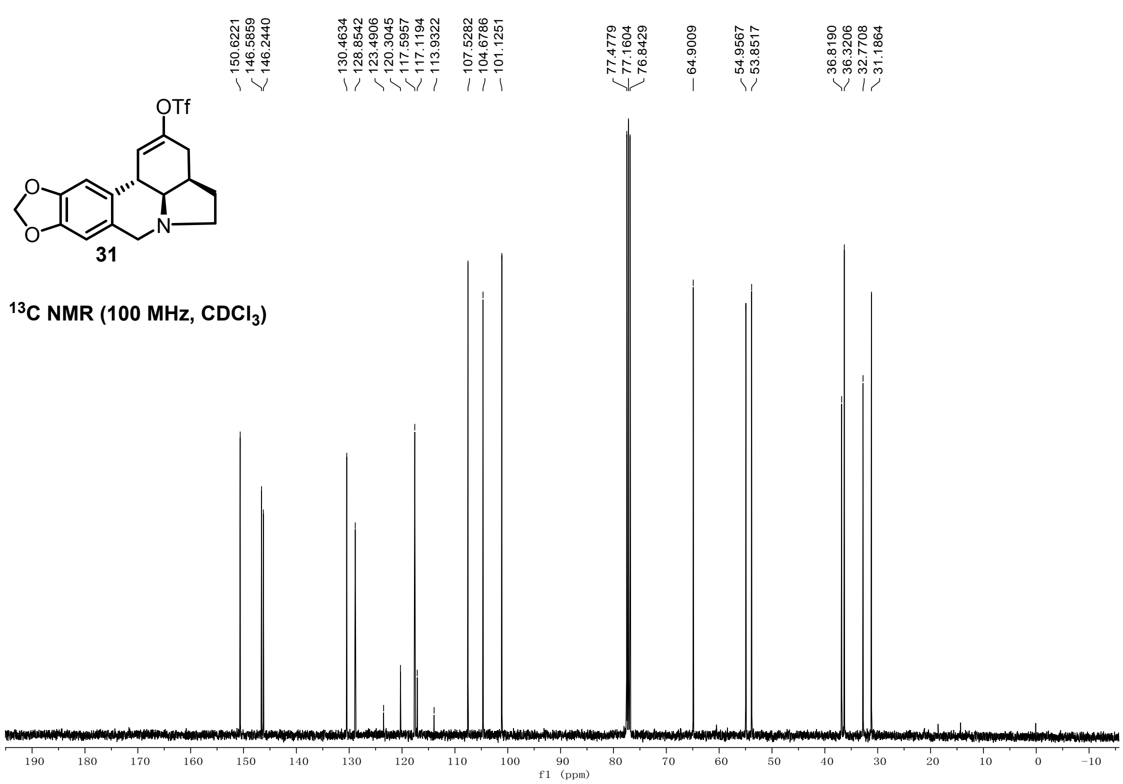

31

^{13}C NMR (100 MHz, $CDCl_3$)

DEPT 90

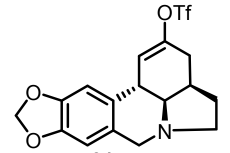

31

^{13}C NMR (100 MHz, $CDCl_3$)

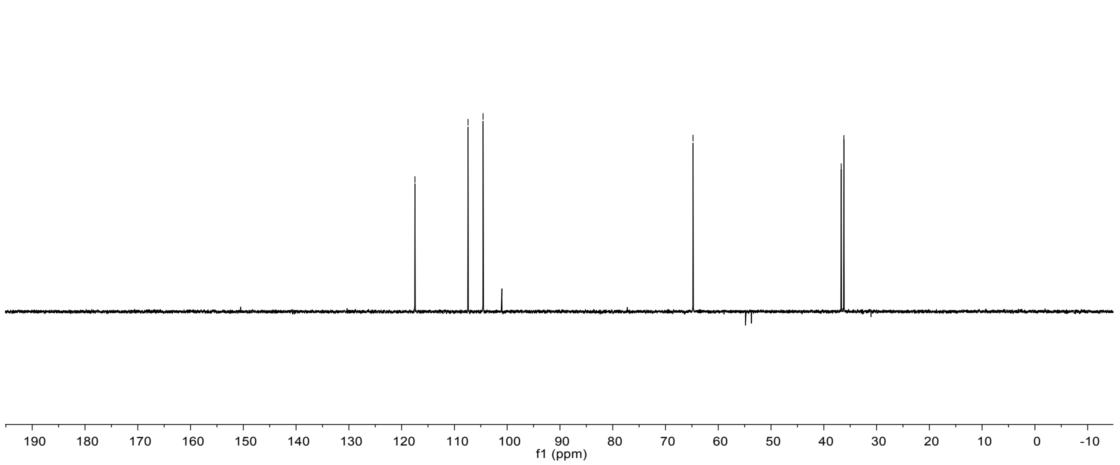

DEPT 135

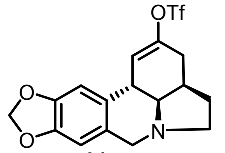

31

^{13}C NMR (100 MHz, $CDCl_3$)

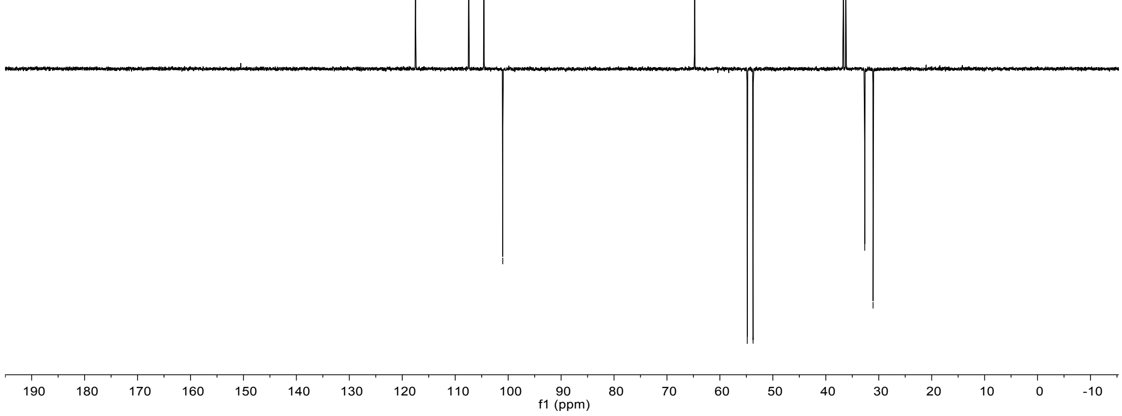

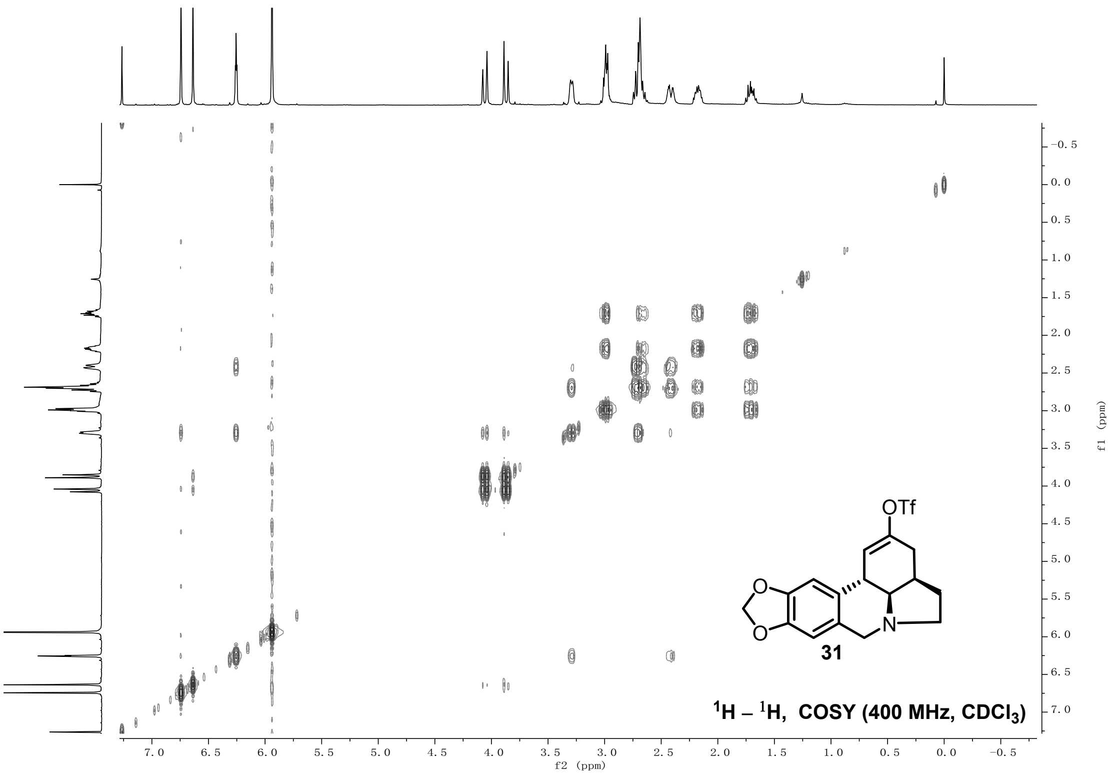

OTf
O
O
N
31
1H – 1H, COSY (400 MHz, CDCl3)
f2 (ppm)
f1 (ppm)
7.0 6.5 6.0 5.5 5.0 4.5 4.0 3.5 3.0 2.5 2.0 1.5 1.0 0.5 0.0 -0.5
-0.5 0.0 0.5 1.0 1.5 2.0 2.5 3.0 3.5 4.0 4.5 5.0 5.5 6.0 6.5 7.0

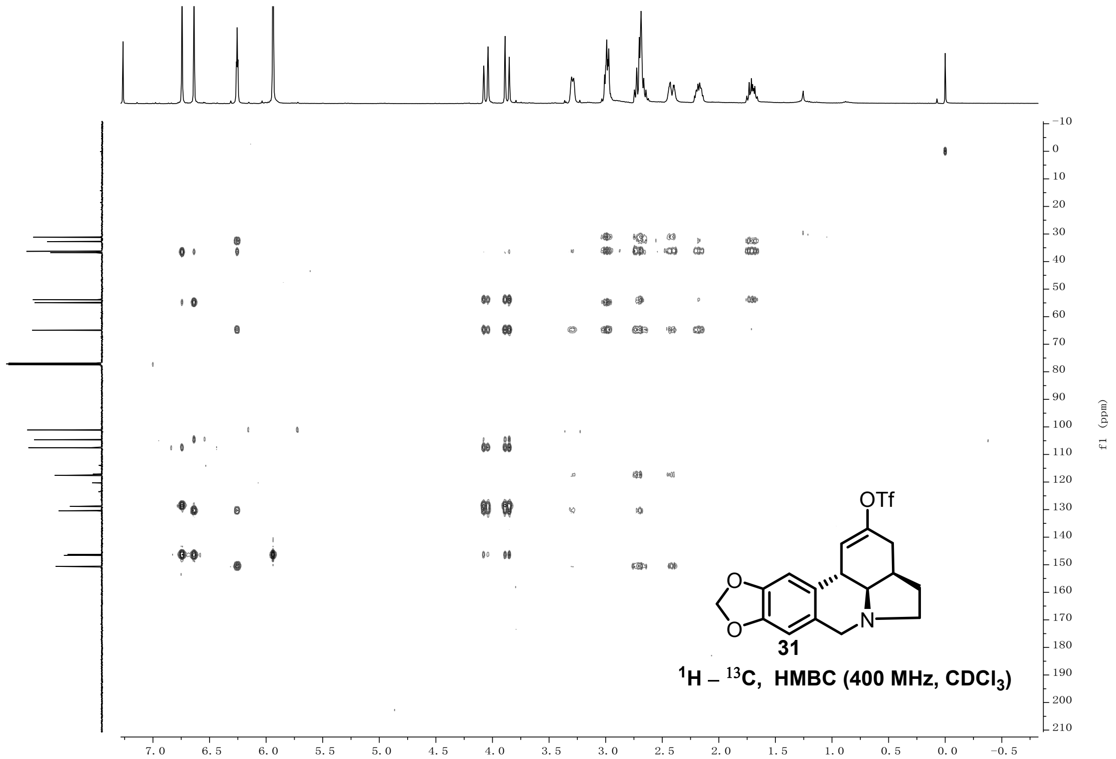

OTf
O
O
N
31
1H – 13C, HMBC (400 MHz, CDCl3)
f2 (ppm)
f1 (ppm)
7.0 6.5 6.0 5.5 5.0 4.5 4.0 3.5 3.0 2.5 2.0 1.5 1.0 0.5 0.0 -0.5
-10 0 10 20 30 40 50 60 70 80 90 100 110 120 130 140 150 160 170 180 190 200 210

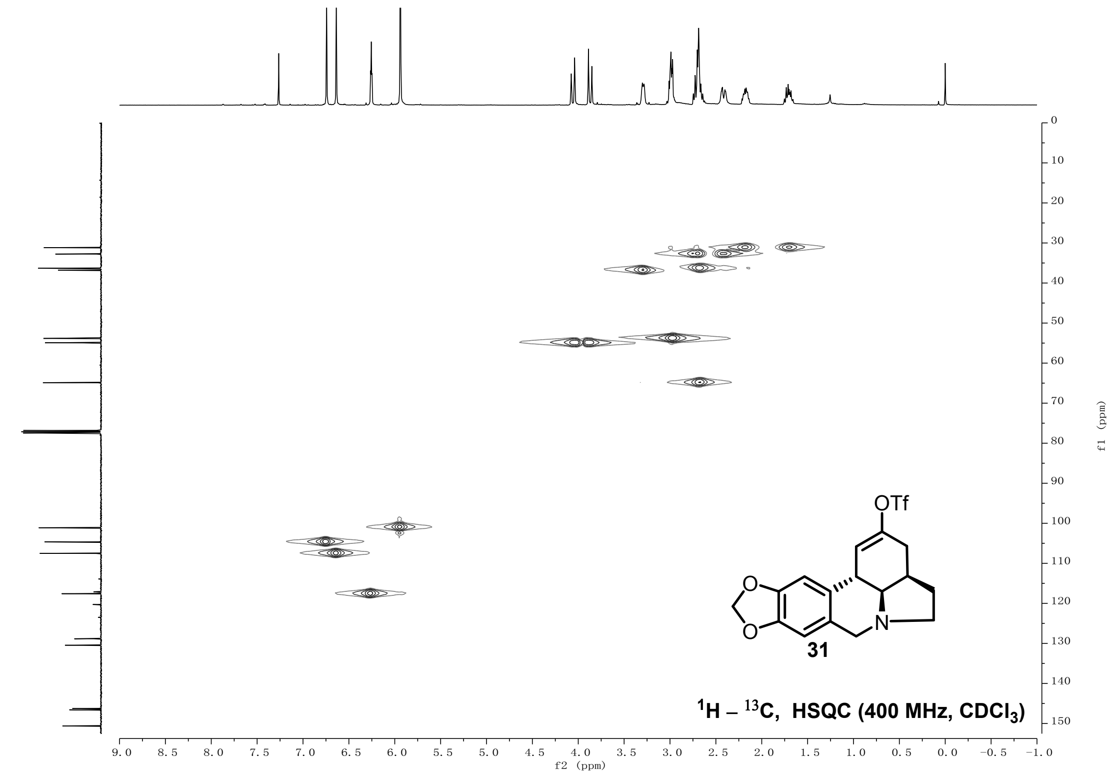
OTf
31
^{1}H – ^{13}C, HSQC (400 MHz, $CDCl_3$)
f2 (ppm)
f1 (ppm)

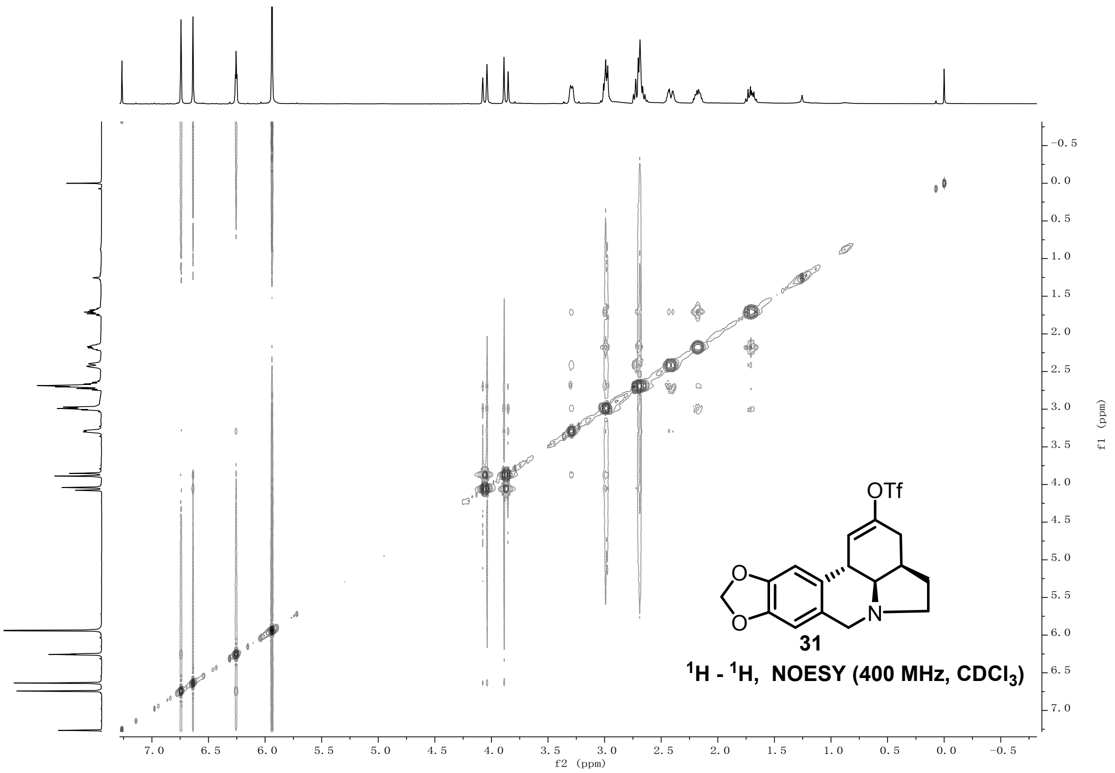
OTf
31
^{1}H - ^{1}H, NOESY (400 MHz, $CDCl_3$)
f2 (ppm)
f1 (ppm)

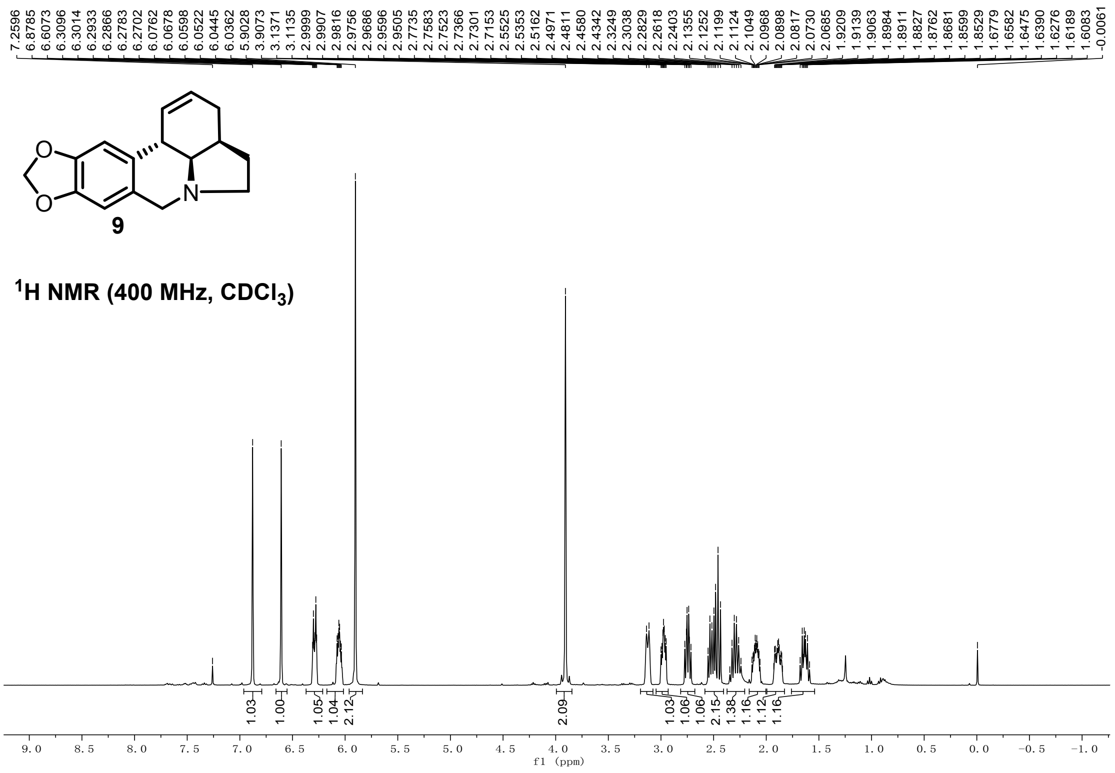
7.2596
6.8785
6.6073
6.3096
6.3014
6.2933
6.2866
6.2783
6.2702
6.0762
6.0678
6.0598
6.0522
6.0445
6.0362
5.9028
3.9073
3.1371
3.1135
2.9999
2.9907
2.9816
2.9756
2.9686
2.9596
2.9505
2.7735
2.7583
2.7523
2.7366
2.7301
2.7153
2.5525
2.5353
2.5162
2.4971
2.4811
2.4580
2.4342
2.3249
2.3038
2.2829
2.2618
2.2403
2.1355
2.1252
2.1199
2.1124
2.1049
2.0968
2.0898
2.0817
2.0730
2.0685
1.9209
1.9139
1.9063
1.8984
1.8911
1.8827
1.8762
1.8681
1.8599
1.8529
1.6779
1.6582
1.6475
1.6390
1.6276
1.6189
1.6083
-0.0061
9
1H NMR (400 MHz, CDCl3)
1.03
1.00
1.05
1.04
2.12
2.09
1.03
1.06
1.06
2.15
1.38
1.16
1.12
1.16
f1 (ppm)

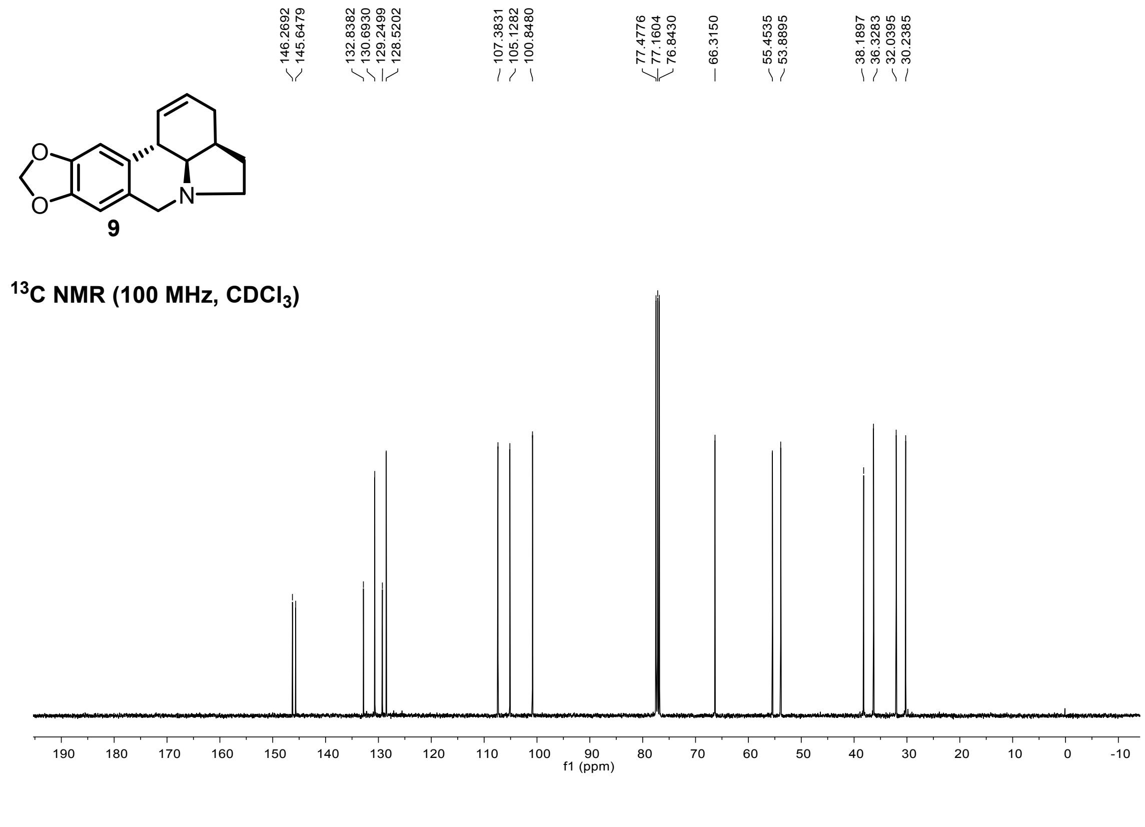
146.2692
145.6479
132.8382
130.6930
129.2499
128.5202
107.3831
105.1282
100.8480
77.4776
77.1604
76.8430
66.3150
55.4535
53.8895
38.1897
36.3283
32.0395
30.2385
9
13C NMR (100 MHz, CDCl3)
f1 (ppm)

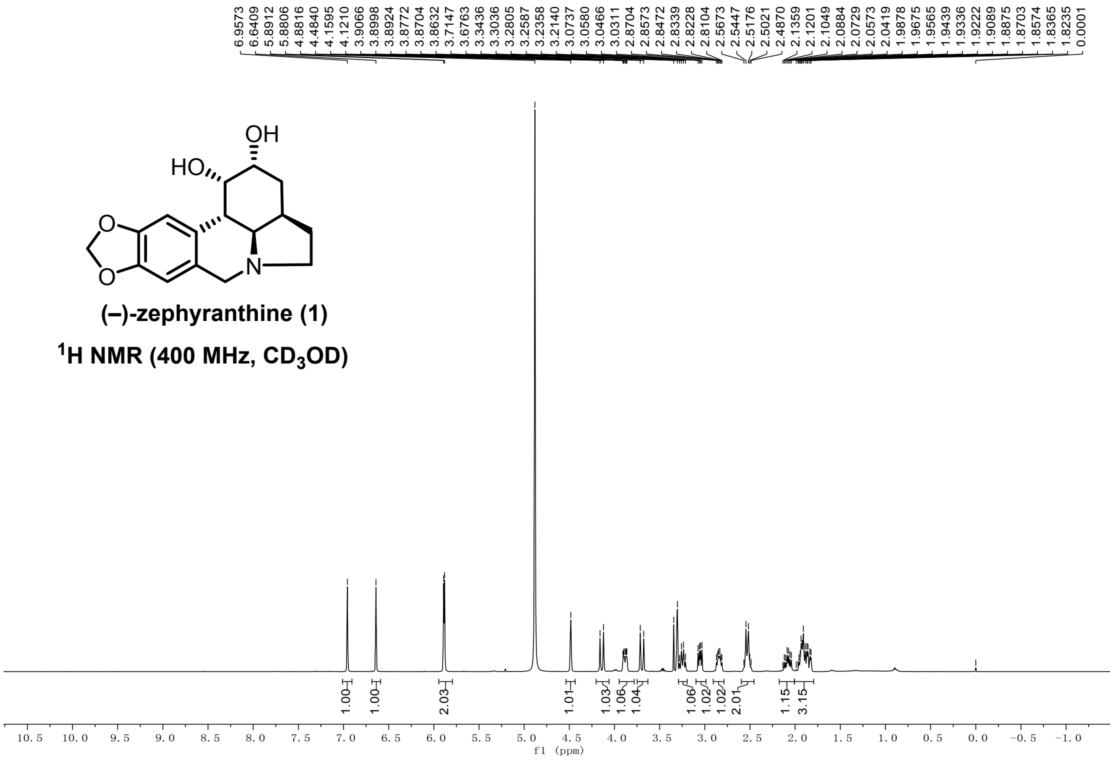
6.9573
6.6409
5.8912
5.8806
4.8816
4.4840
4.1595
4.1210
3.9066
3.8998
3.8924
3.8772
3.8704
3.8632
3.7147
3.6763
3.3436
3.3036
3.2805
3.2587
3.2358
3.2140
3.0737
3.0580
3.0466
3.0311
2.8704
2.8573
2.8472
2.8339
2.8228
2.8104
2.5673
2.5447
2.5176
2.5021
2.4870
2.1359
2.1201
2.1049
2.0884
2.0729
2.0573
2.0419
1.9878
1.9675
1.9565
1.9439
1.9336
1.9222
1.9089
1.8875
1.8703
1.8574
1.8365
1.8235
0.0001
OH
HO
O
O
N
(–)-zephyranthine (1)
1H NMR (400 MHz, CD3OD)
1.00
1.00
2.03
1.01
1.03
1.06
1.04
1.06
1.02
1.02
2.01
1.15
3.15
f1 (ppm)

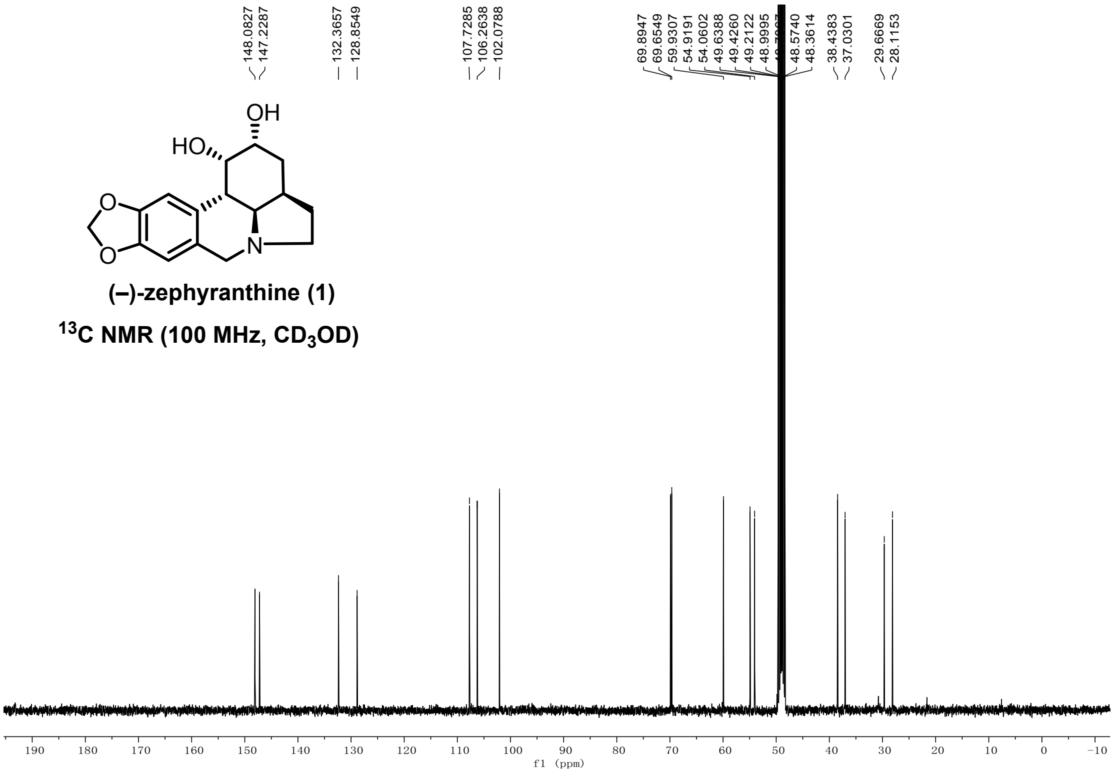
148.0827
147.2287
132.3657
128.8549
107.7285
106.2638
102.0788
69.8947
69.6549
59.9307
54.9191
54.0602
49.6388
49.4260
49.2122
48.9995
48.5740
48.3614
38.4383
37.0301
29.6669
28.1153
OH
HO
O
O
N
(–)-zephyranthine (1)
13C NMR (100 MHz, CD3OD)
f1 (ppm)

DEPT 90

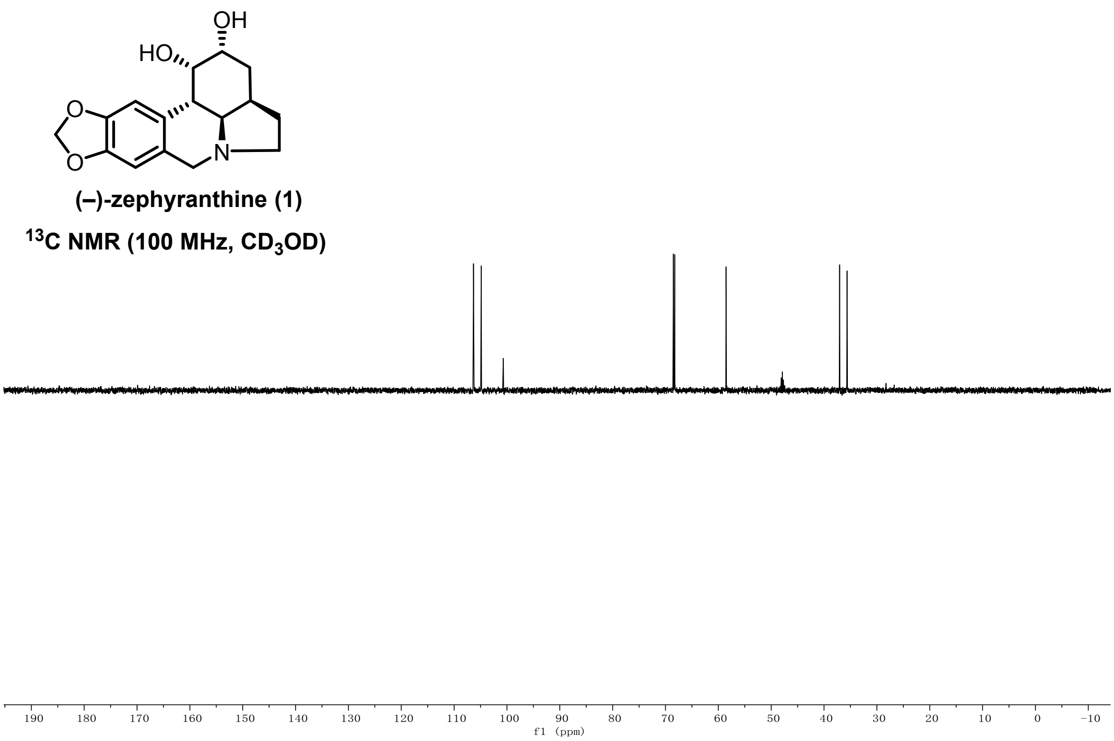

DEPT 135

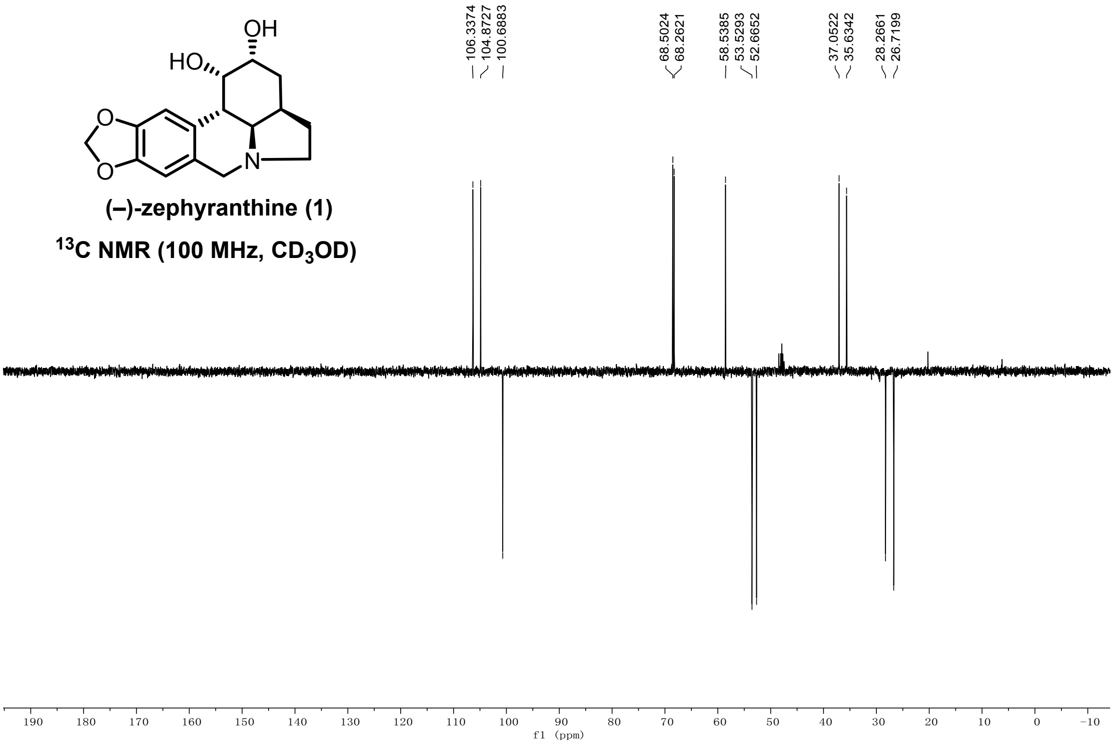

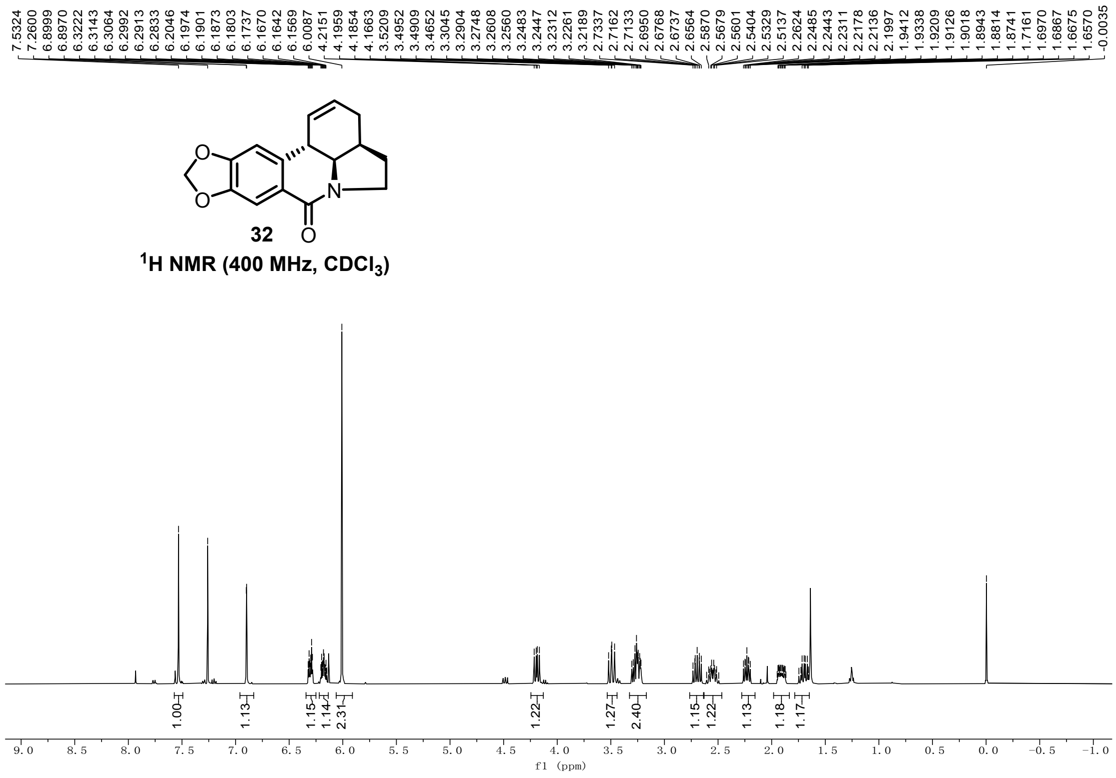
7.5324
7.2600
6.8999
6.8970
6.3222
6.3143
6.3064
6.2992
6.2913
6.2833
6.2046
6.1974
6.1901
6.1873
6.1803
6.1737
6.1670
6.1642
6.1569
6.0087
4.2151
4.1959
4.1854
4.1663
3.5209
3.4952
3.4909
3.4652
3.3045
3.2904
3.2748
3.2608
3.2560
3.2483
3.2447
3.2312
3.2261
3.2189
2.7337
2.7162
2.7133
2.6950
2.6768
2.6737
2.6564
2.5870
2.5679
2.5601
2.5404
2.5329
2.5137
2.2624
2.2485
2.2443
2.2311
2.2178
2.2136
2.1997
1.9412
1.9338
1.9209
1.9126
1.9018
1.8943
1.8814
1.8741
1.7161
1.6970
1.6867
1.6675
1.6570
-0.0035
32
^{1}H NMR (400 MHz, CDCl$_3$)
1.00
1.13
1.15
1.14
2.31
1.22
1.27
2.40
1.15
1.22
1.13
1.18
1.17
f1 (ppm)

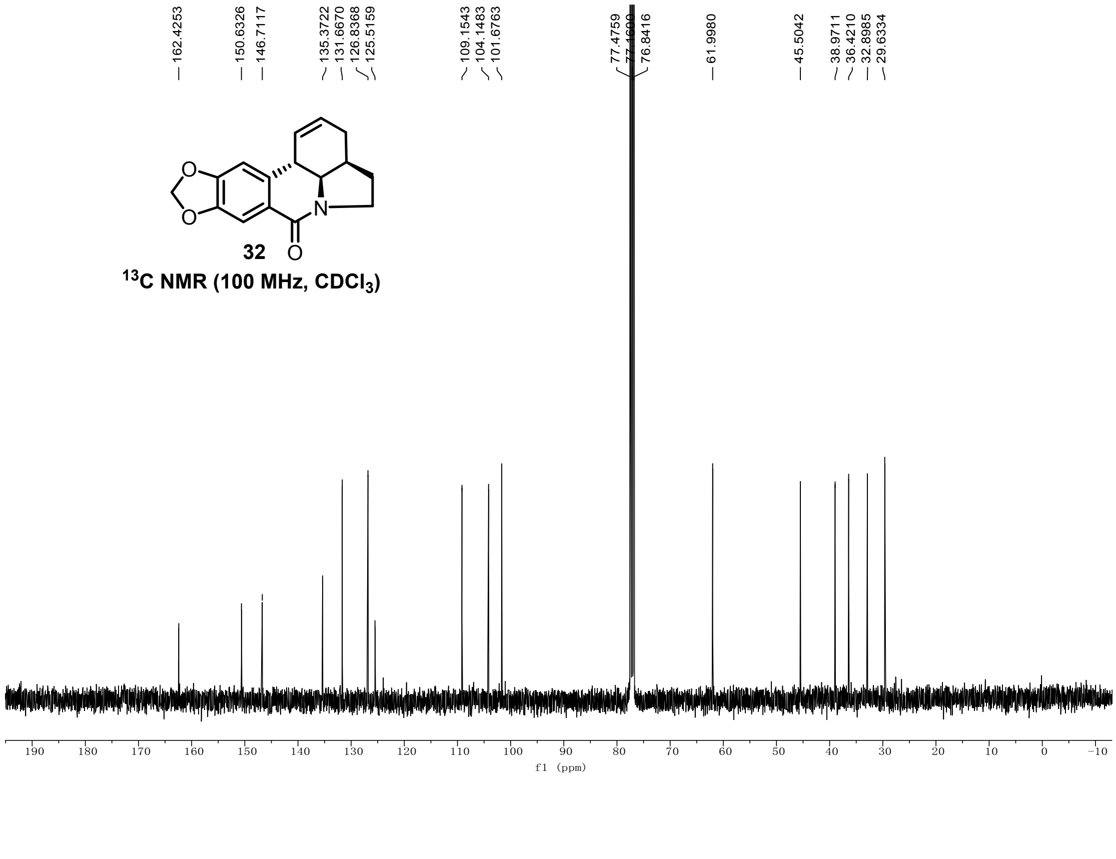
162.4253
150.6326
146.7117
135.3722
131.6670
126.8368
125.5159
109.1543
104.1483
101.6763
77.4759
76.8416
61.9980
45.5042
38.9711
36.4210
32.8985
29.6334
32
^{13}C NMR (100 MHz, CDCl$_3$)
f1 (ppm)

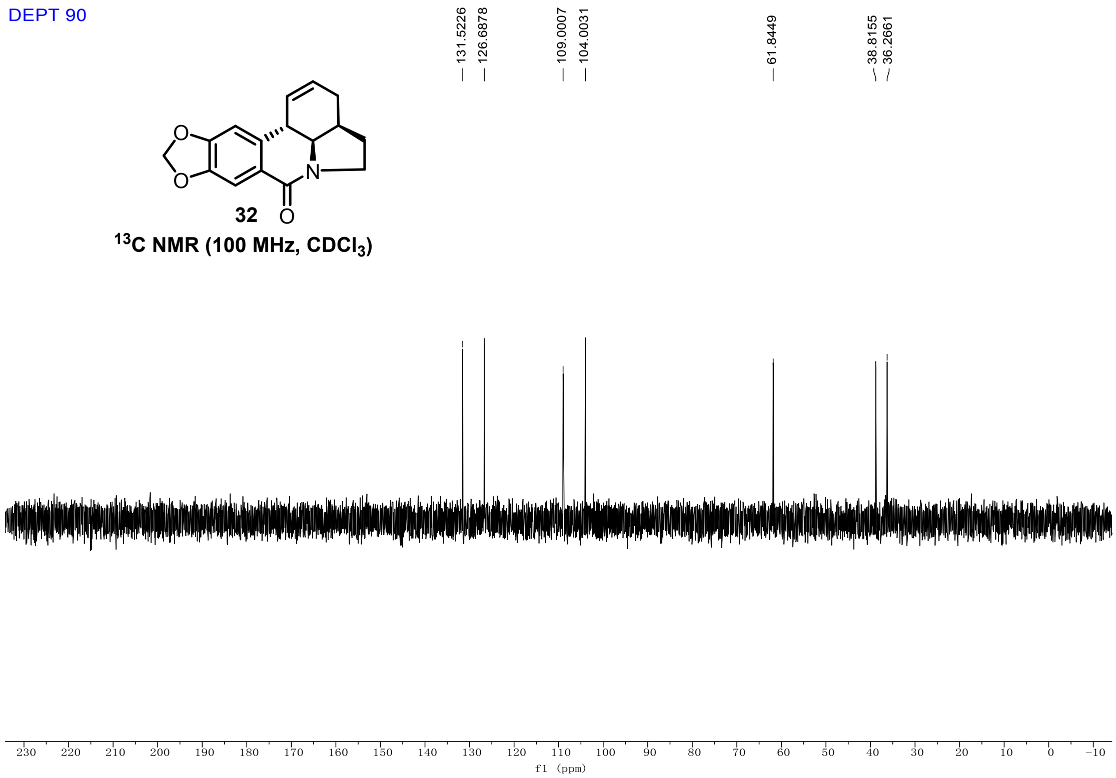
DEPT 90
131.5226
126.6878
109.0007
104.0031
61.8449
38.8155
36.2661
32
13C NMR (100 MHz, CDCl3)
230 220 210 200 190 180 170 160 150 140 130 120 110 100 90 80 70 60 50 40 30 20 10 0 -10
f1 (ppm)

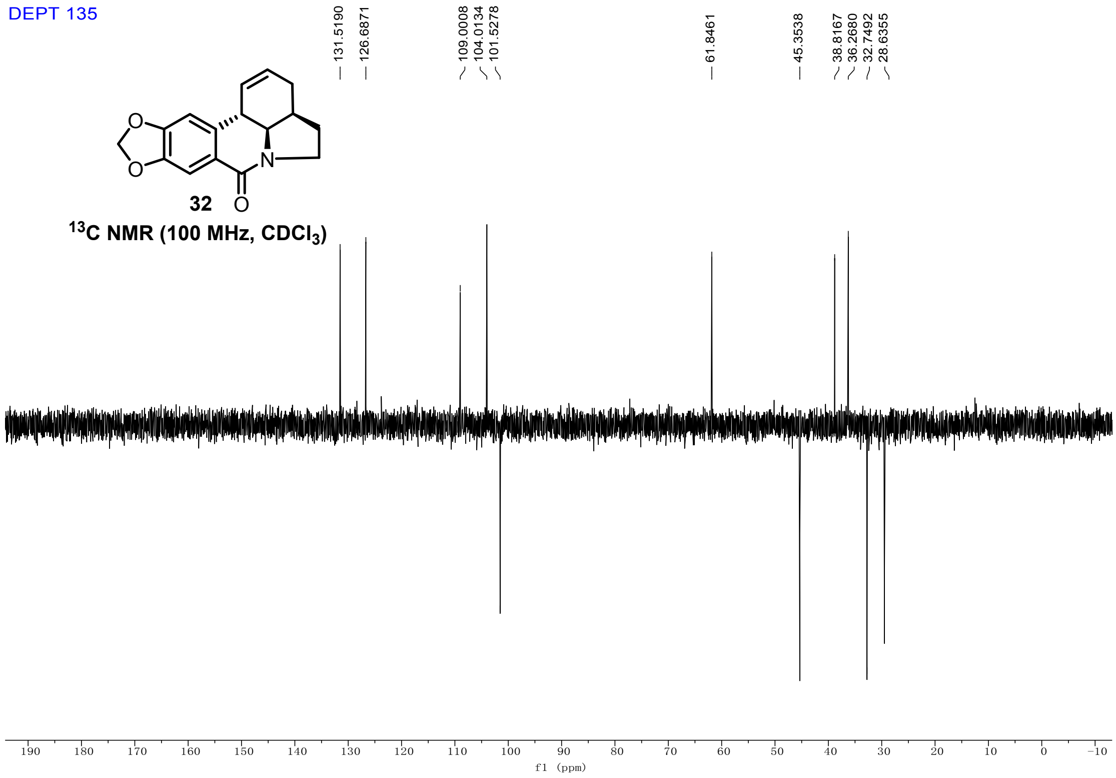
DEPT 135
131.5190
126.6871
109.0008
104.0134
101.5278
61.8461
45.3538
38.8167
36.2680
32.7492
28.6355
32
13C NMR (100 MHz, CDCl3)
190 180 170 160 150 140 130 120 110 100 90 80 70 60 50 40 30 20 10 0 -10
f1 (ppm)

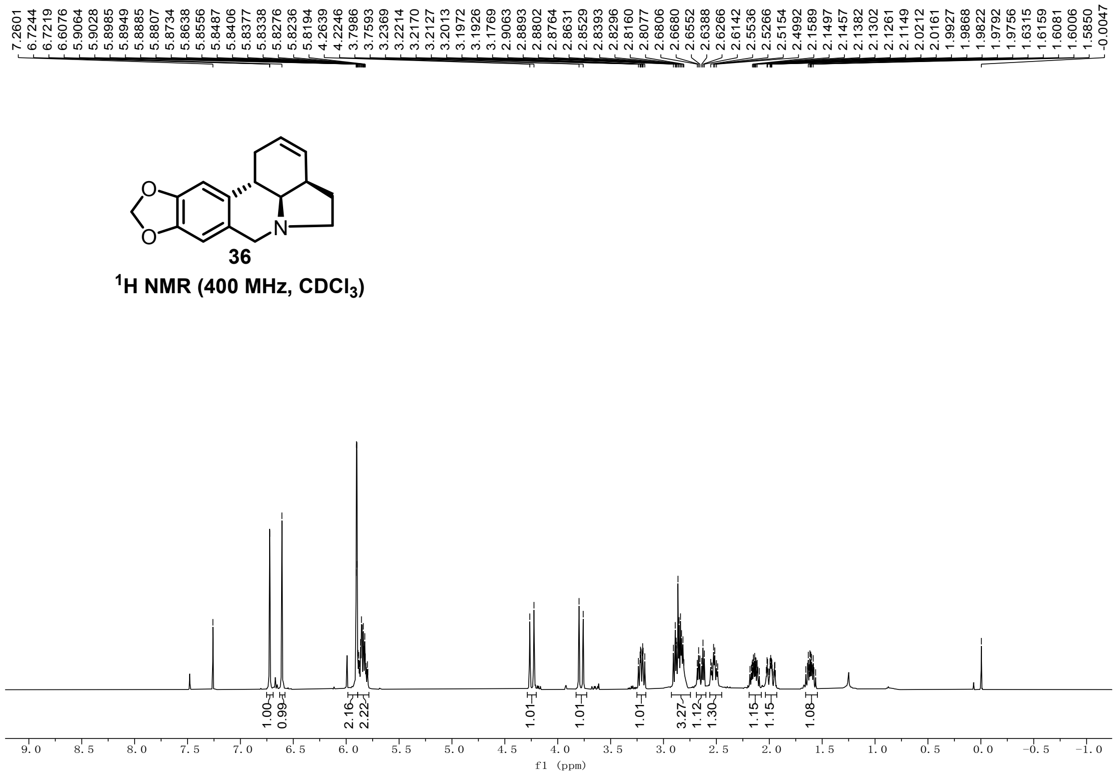
7.2601
6.7244
6.7219
6.6076
5.9064
5.9028
5.8985
5.8949
5.8885
5.8807
5.8734
5.8638
5.8556
5.8487
5.8406
5.8377
5.8338
5.8276
5.8236
5.8194
4.2639
4.2246
3.7986
3.7593
3.2369
3.2214
3.2170
3.2127
3.2013
3.1972
3.1926
3.1769
2.9063
2.8893
2.8802
2.8764
2.8631
2.8529
2.8393
2.8296
2.8160
2.8077
2.6806
2.6680
2.6552
2.6388
2.6266
2.6142
2.5536
2.5266
2.5154
2.4992
2.1589
2.1497
2.1457
2.1382
2.1302
2.1261
2.1149
2.0212
2.0161
1.9927
1.9868
1.9822
1.9792
1.9756
1.6315
1.6159
1.6081
1.6006
1.5850
-0.0047
36
1H NMR (400 MHz, CDCl3)
1.00
0.99
2.16
2.22
1.01
1.01
1.01
3.27
1.12
1.30
1.15
1.15
1.08
f1 (ppm)

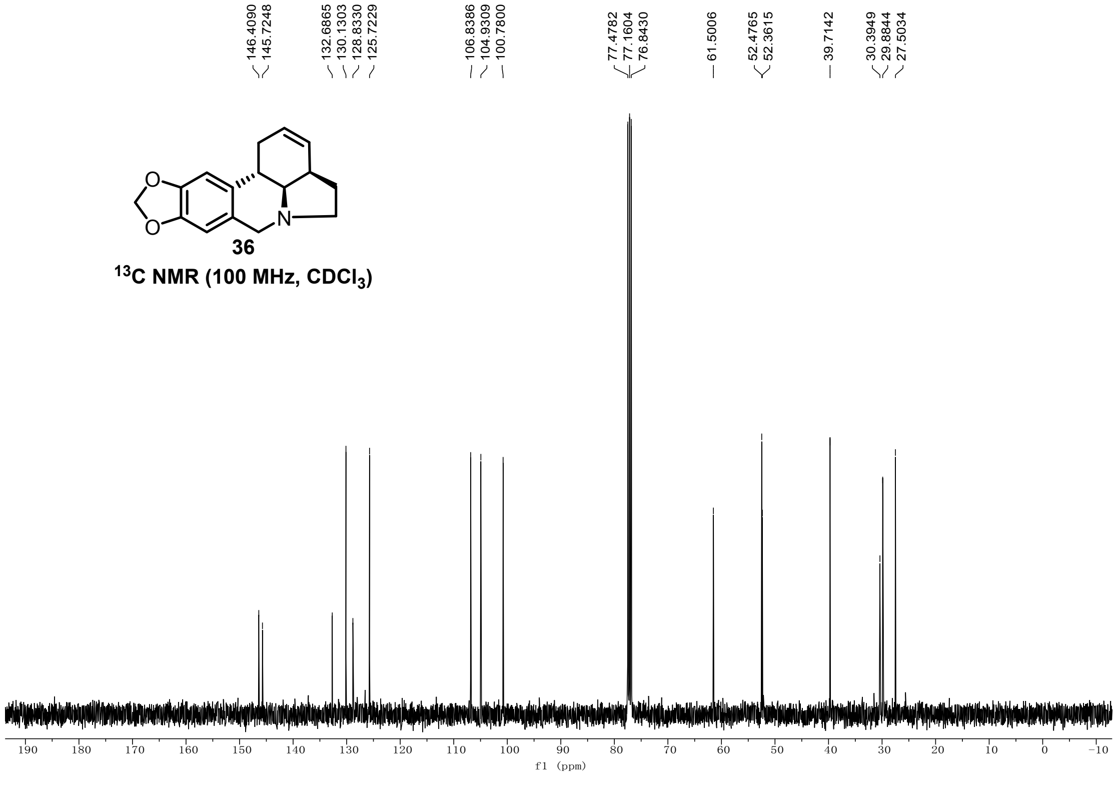
146.4090
145.7248
132.6865
130.1303
128.8330
125.7229
106.8386
104.9309
100.7800
77.4782
77.1604
76.8430
61.5006
52.4765
52.3615
39.7142
30.3949
29.8844
27.5034
36
13C NMR (100 MHz, CDCl3)
f1 (ppm)

DEPT 90

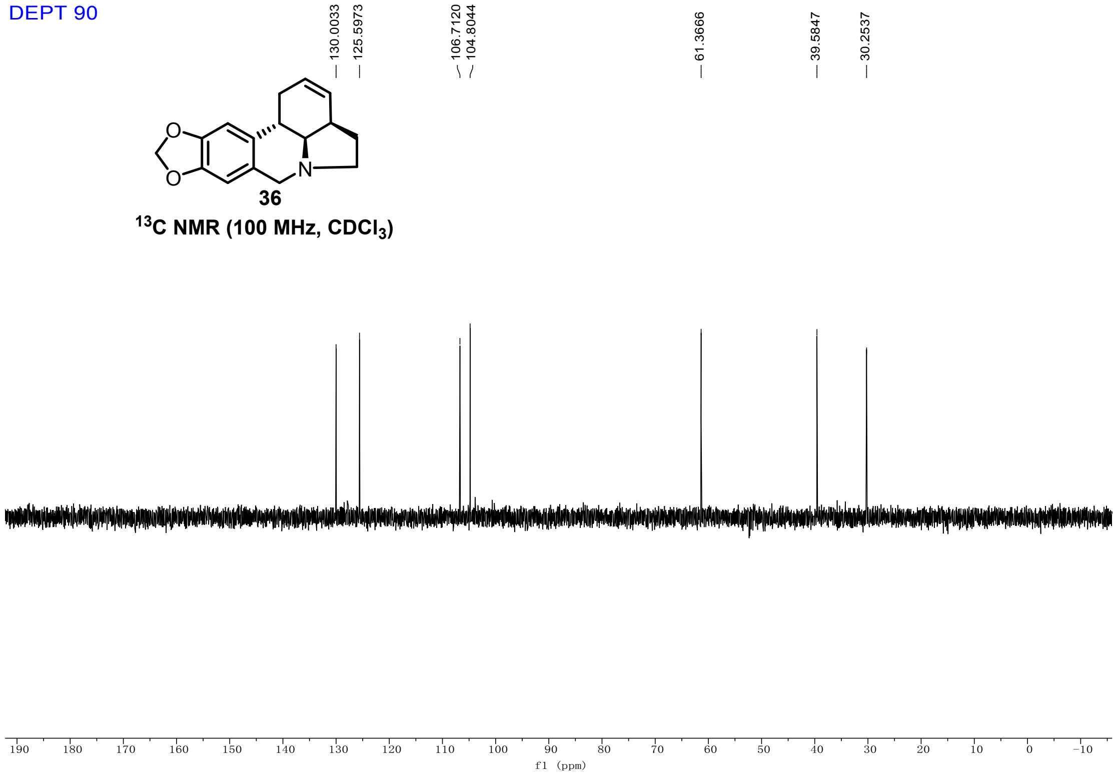

DEPT 135

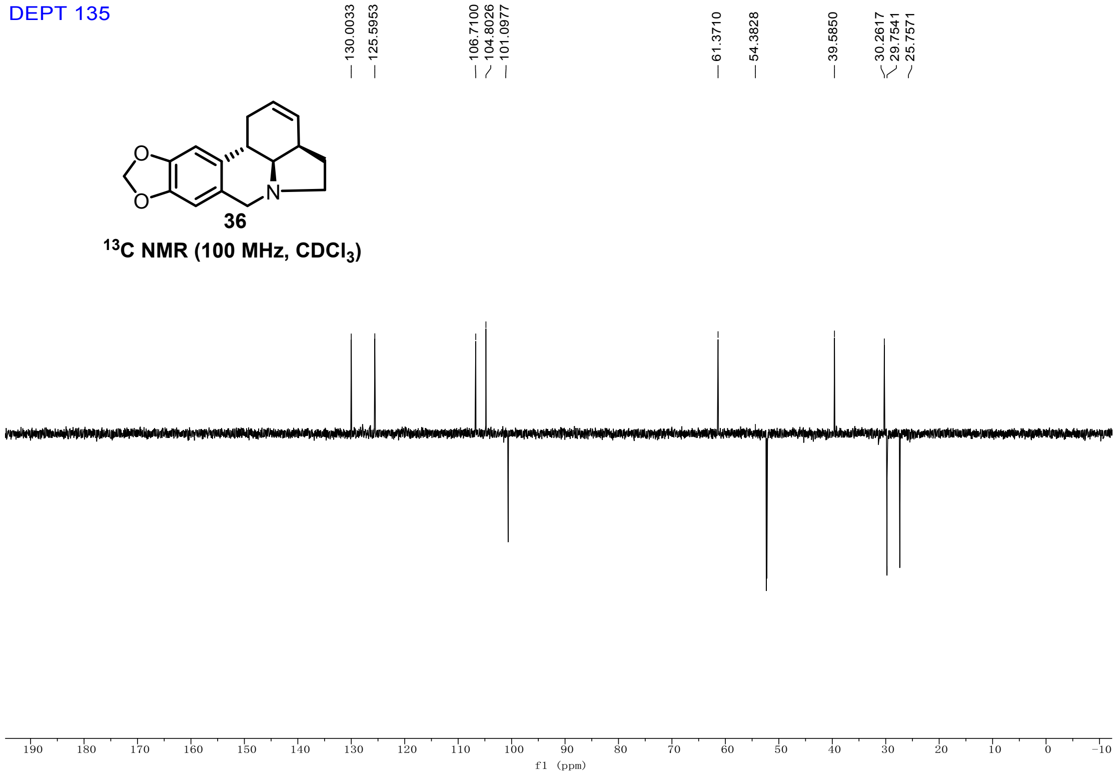

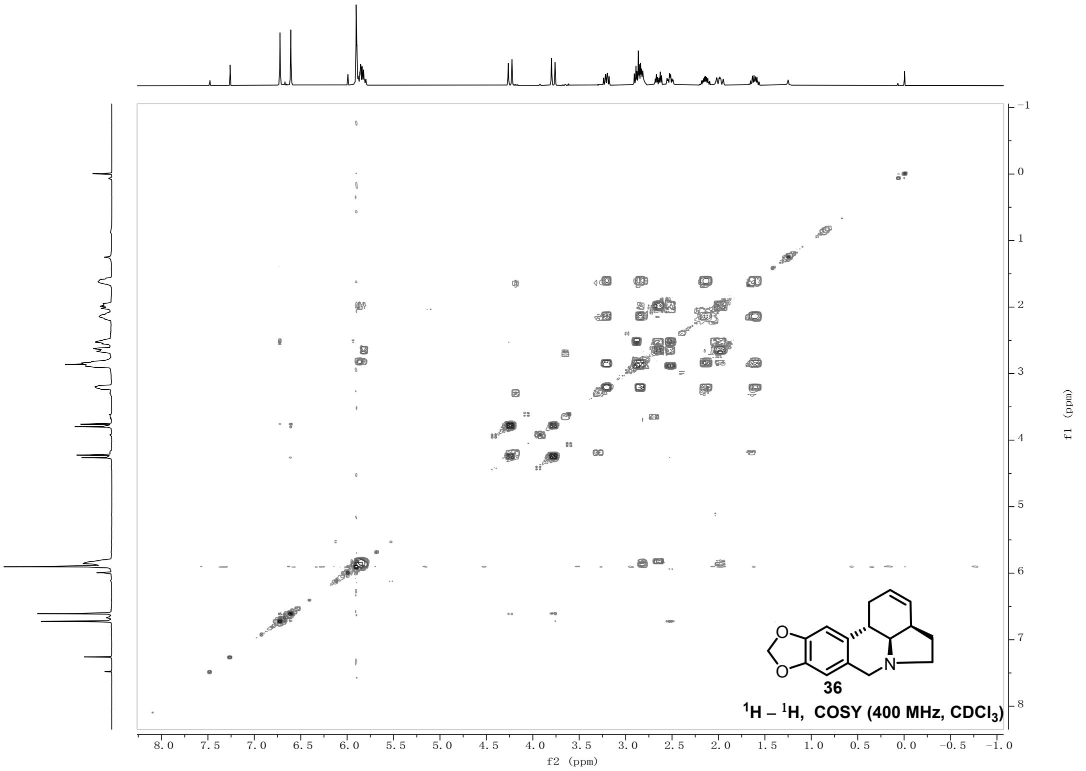
36
1H – 1H, COSY (400 MHz, CDCl3)
f2 (ppm)
f1 (ppm)

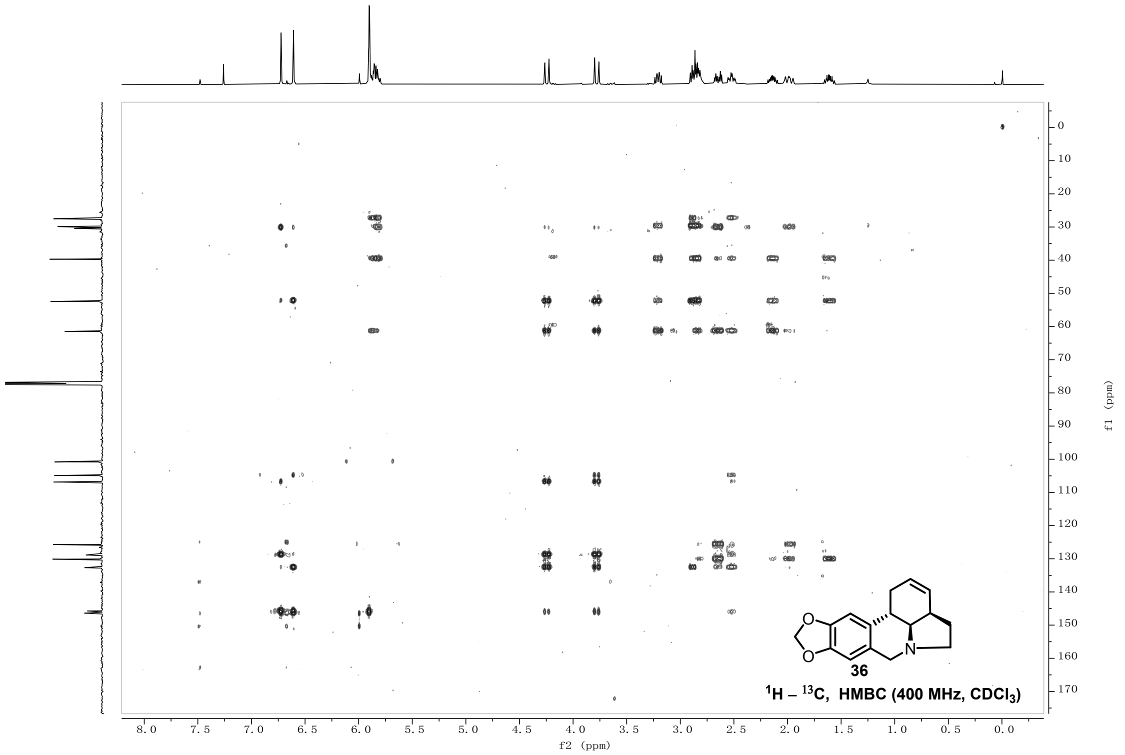
36
1H – 13C, HMBC (400 MHz, CDCl3)
f2 (ppm)
f1 (ppm)

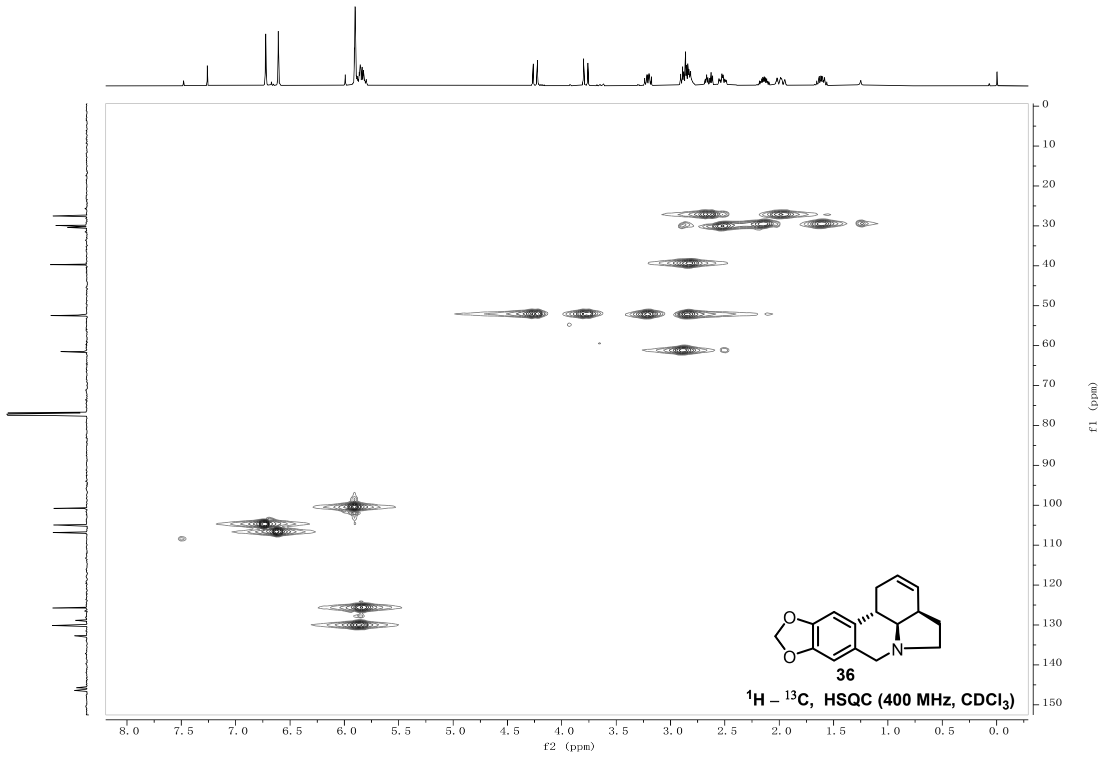

36
1H – 13C, HSQC (400 MHz, CDCl3)
f2 (ppm)
f1 (ppm)

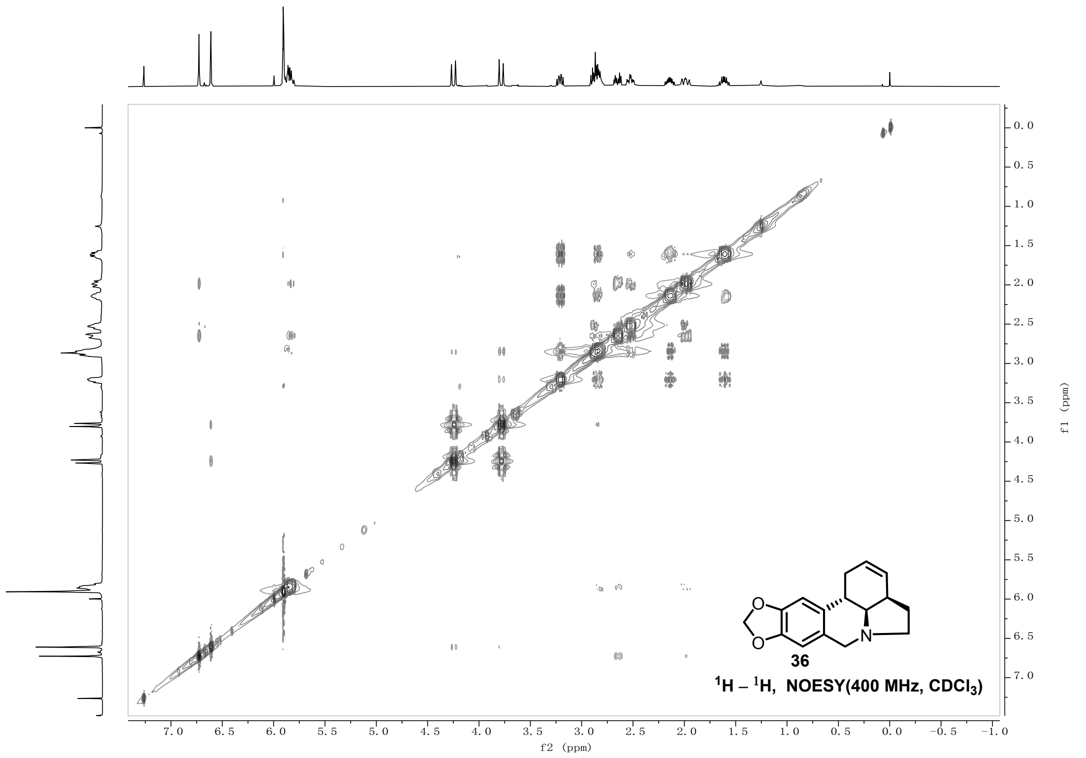

36
1H – 1H, NOESY(400 MHz, CDCl3)
f2 (ppm)
f1 (ppm)

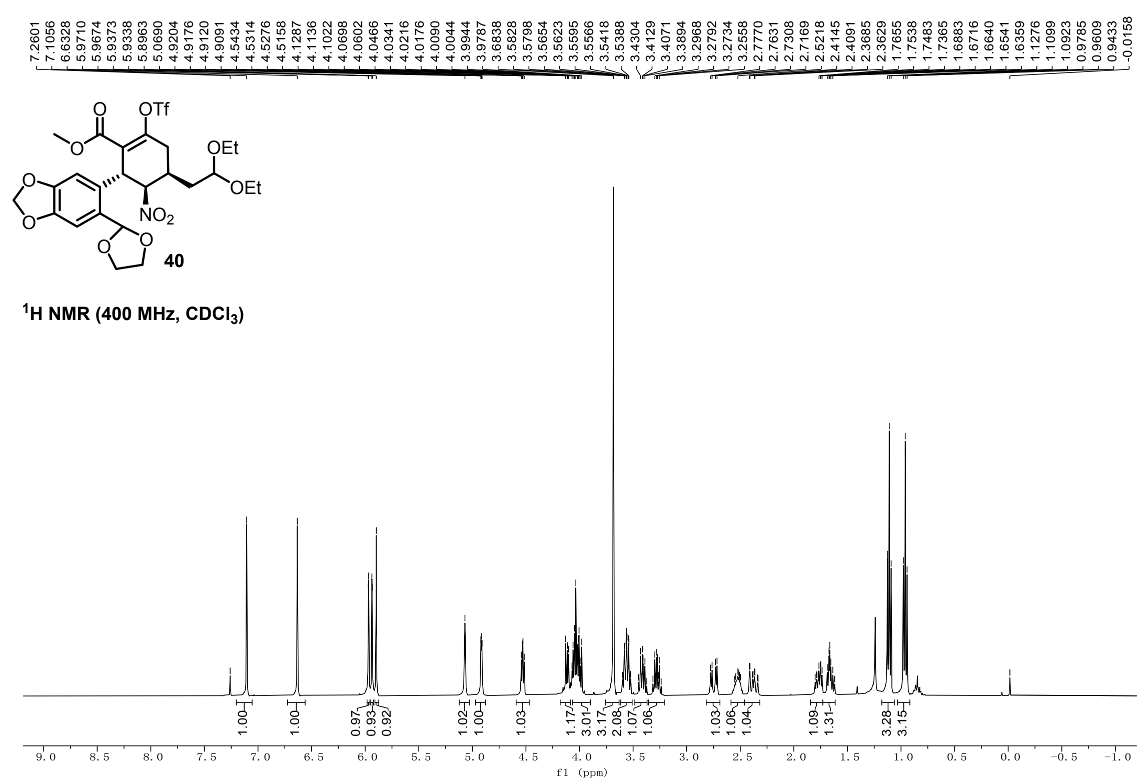
7.2601
7.1056
6.6328
5.9710
5.9674
5.9373
5.9338
5.8963
5.0690
4.9204
4.9176
4.9120
4.9091
4.5434
4.5314
4.5276
4.5158
4.1287
4.1136
4.1022
4.0698
4.0602
4.0466
4.0341
4.0216
4.0176
4.0090
4.0044
3.9944
3.9787
3.6838
3.5828
3.5798
3.5654
3.5623
3.5595
3.5566
3.5418
3.5388
3.4304
3.4129
3.4071
3.3894
3.2968
3.2792
3.2734
3.2558
2.7770
2.7631
2.7308
2.7169
2.5218
2.4145
2.4091
2.3685
2.3629
1.7655
1.7538
1.7483
1.7365
1.6883
1.6716
1.6640
1.6541
1.6359
1.1276
1.1099
1.0923
0.9785
0.9609
0.9433
-0.0158
OTf
OEt
OEt
NO2
40
1H NMR (400 MHz, CDCl3)
1.00
1.00
0.97
0.93
0.92
1.02
1.00
1.03
1.17
3.01
3.17
2.08
1.07
1.06
1.03
1.06
1.04
1.09
1.31
3.28
3.15
f1 (ppm)

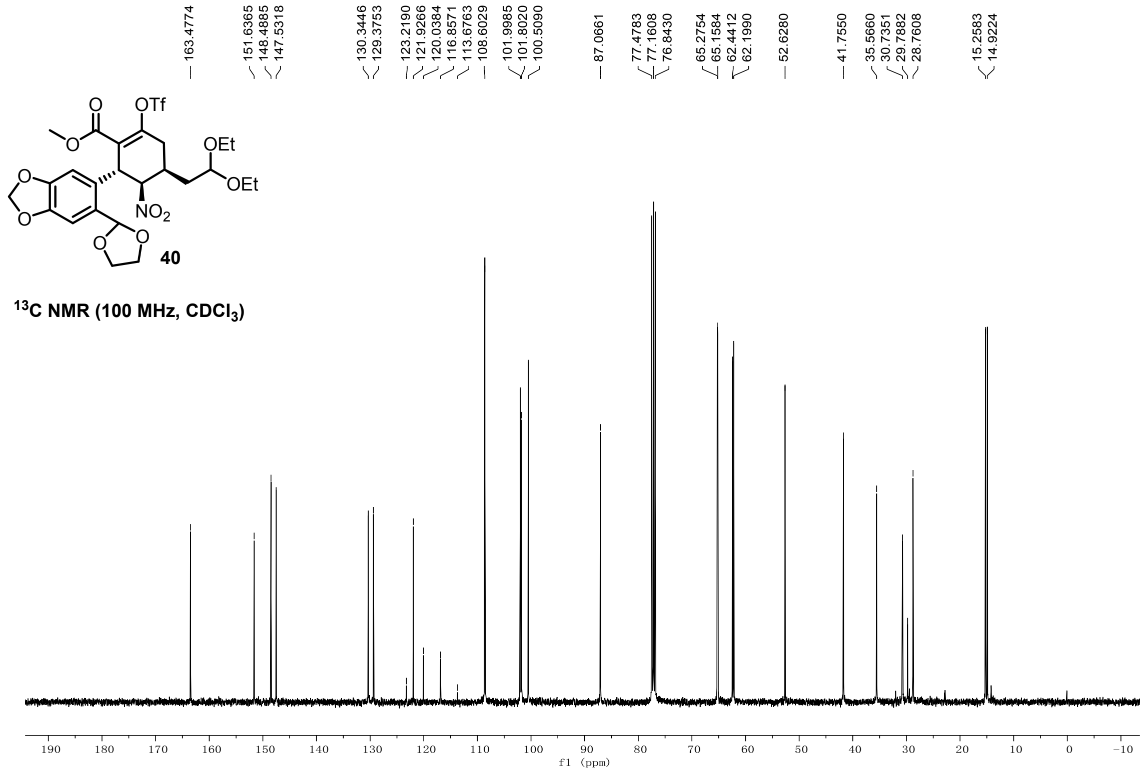
163.4774
151.6365
148.4885
147.5318
130.3446
129.3753
123.2190
121.9266
120.0384
116.8571
113.6763
108.6029
101.9985
101.8020
100.5090
87.0661
77.4783
77.1608
76.8430
65.2754
65.1584
62.4412
62.1990
52.6280
41.7550
35.5660
30.7351
29.7882
28.7608
15.2583
14.9224
OTf
OEt
OEt
NO2
40
13C NMR (100 MHz, CDCl3)
f1 (ppm)

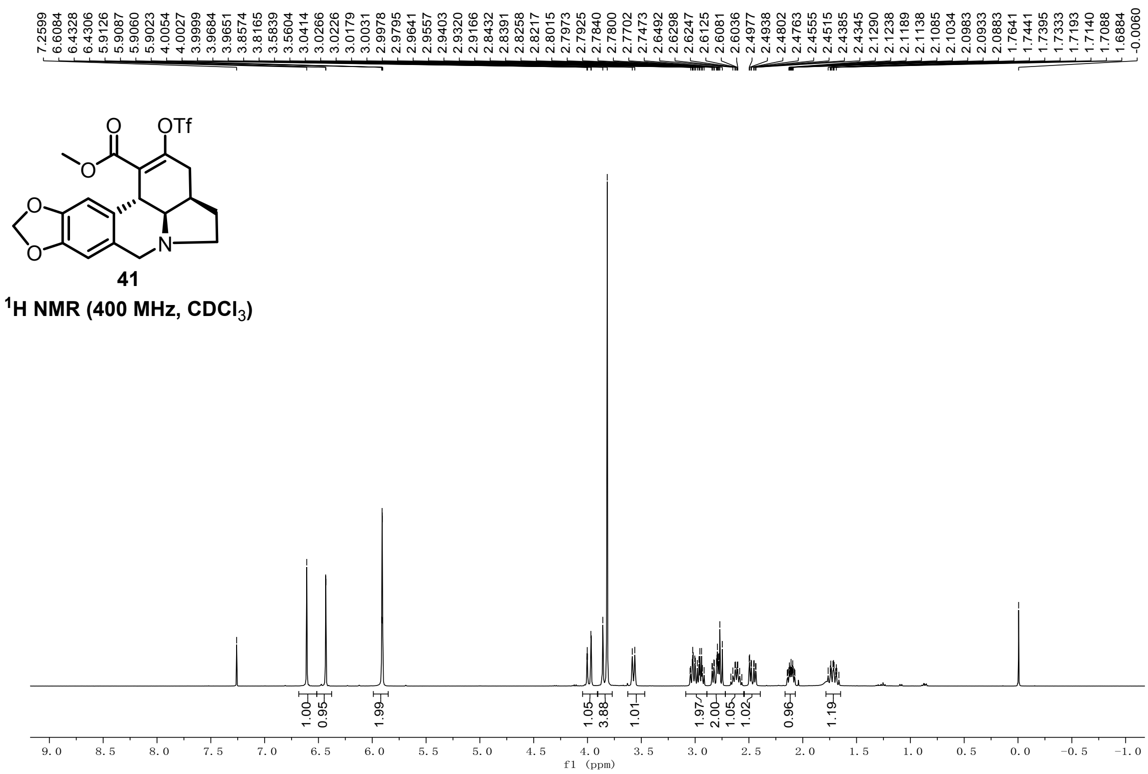
7.2599 6.6084 6.4328 6.4306 5.9126 5.9087 5.9060 5.9023 4.0054 4.0027 3.9999 3.9684 3.9651 3.8574 3.8165 3.5839 3.5604 3.0414 3.0266 3.0226 3.0179 3.0031 2.9978 2.9795 2.9641 2.9557 2.9403 2.9320 2.9166 2.8432 2.8391 2.8258 2.8217 2.8015 2.7973 2.7925 2.7840 2.7800 2.7702 2.7473 2.6492 2.6298 2.6247 2.6125 2.6081 2.6036 2.4977 2.4938 2.4802 2.4763 2.4555 2.4515 2.4385 2.4345 2.1290 2.1238 2.1189 2.1138 2.1085 2.1034 2.0983 2.0933 2.0883 1.7641 1.7441 1.7395 1.7333 1.7193 1.7140 1.7088 1.6884 -0.0060
OTf
O
O
O
N
41
^{1}H NMR (400 MHz, $CDCl_3$)
1.00 0.95 1.99 1.05 3.88 1.01 1.97 2.00 1.05 1.02 0.96 1.19
9.0 8.5 8.0 7.5 7.0 6.5 6.0 5.5 5.0 4.5 4.0 3.5 3.0 2.5 2.0 1.5 1.0 0.5 0.0 -0.5 -1.0
f1 (ppm)

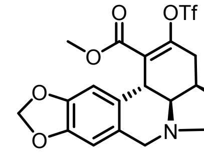

41

^{13}C NMR (100 MHz, $CDCl_3$)

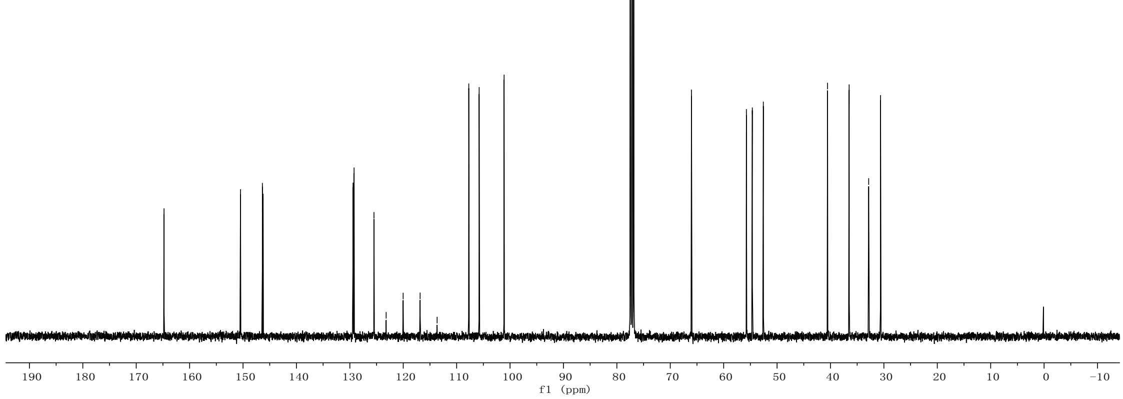

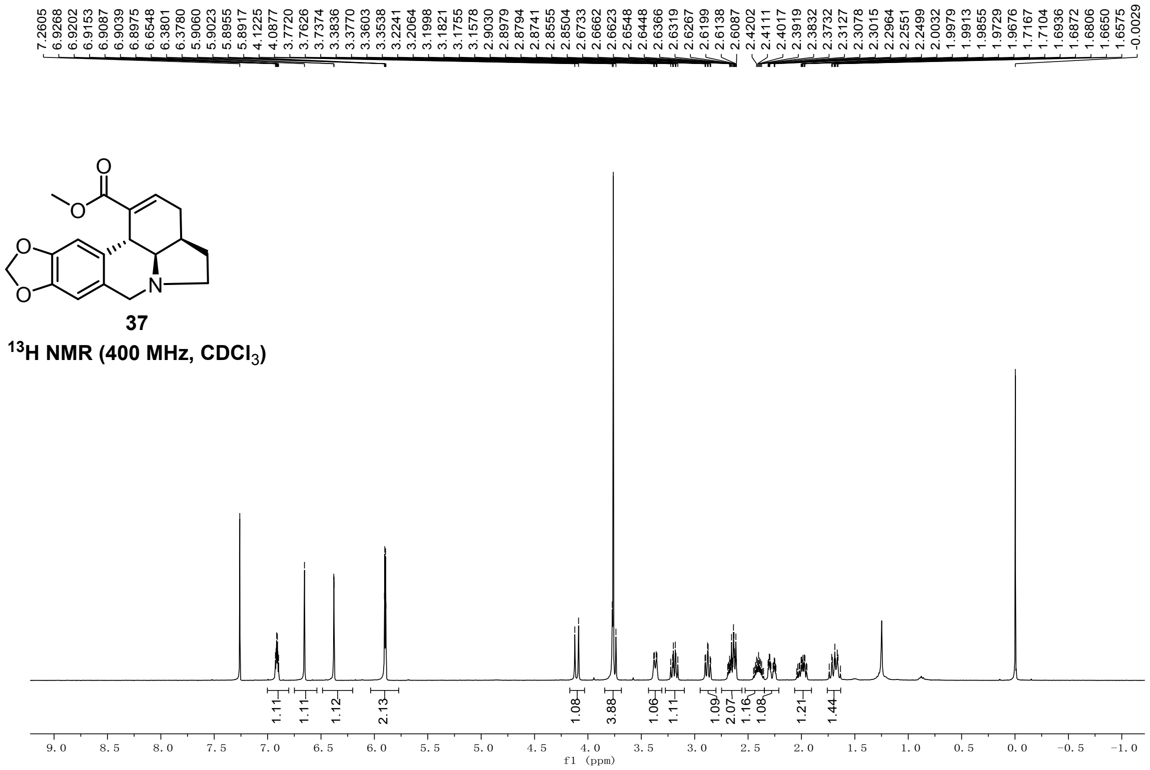

37

^{13}H NMR (400 MHz, CDCl$_3$)

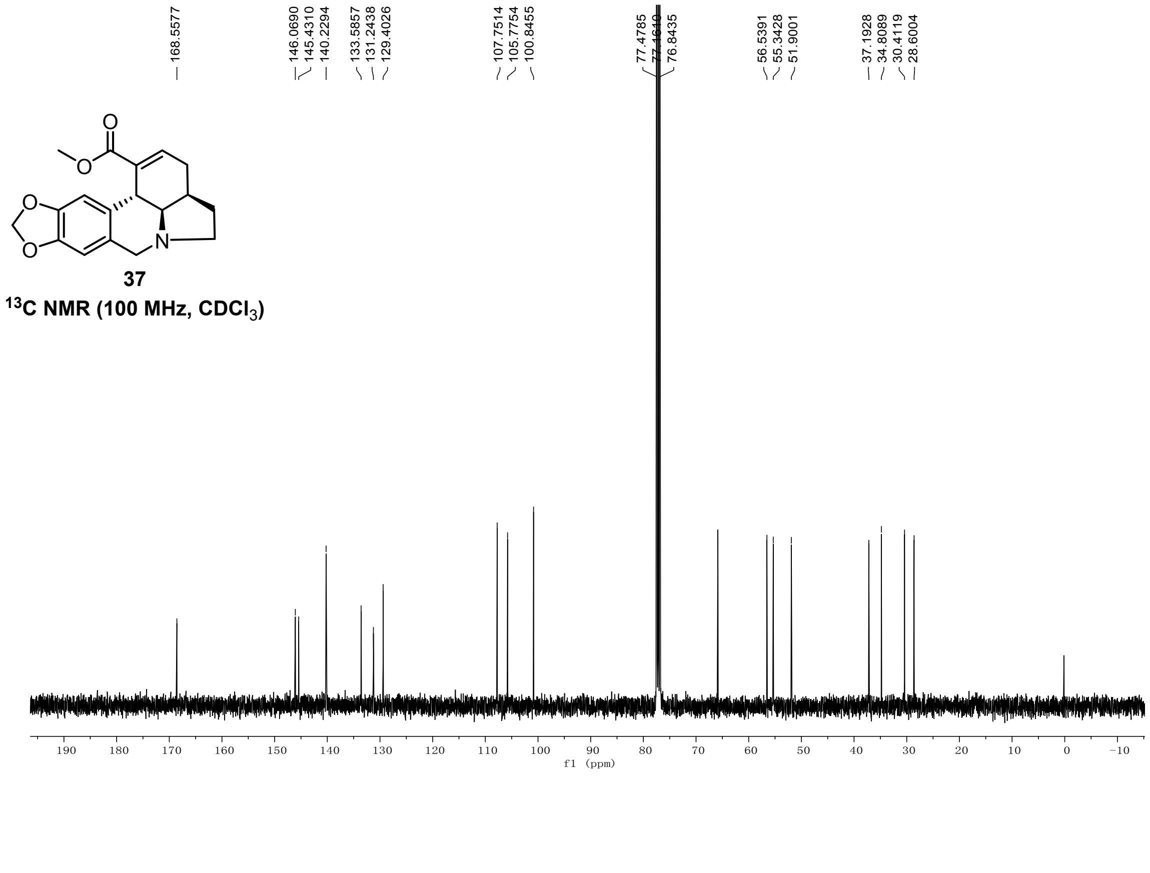

37

^{13}C NMR (100 MHz, CDCl$_3$)

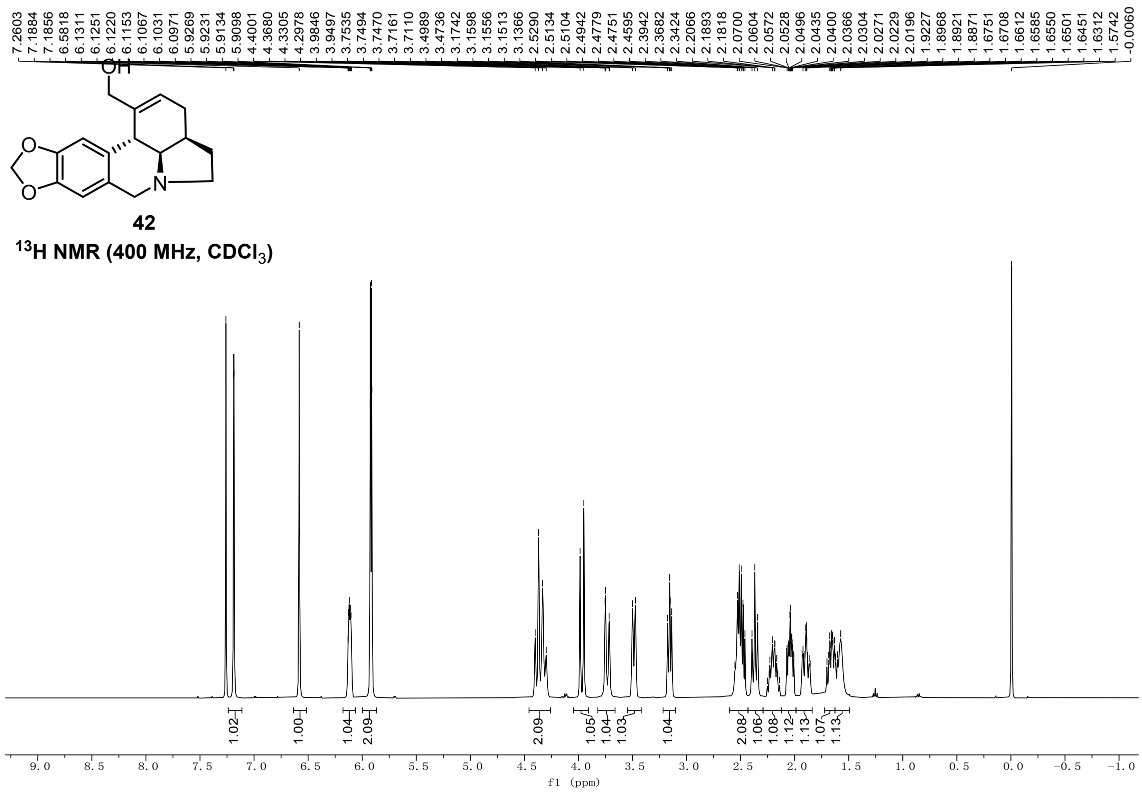
7.2603
7.1884
7.1856
6.5818
6.1311
6.1251
6.1220
6.1153
6.1067
6.1031
6.0971
5.9269
5.9231
5.9134
5.9098
4.4001
4.3680
4.3305
4.2978
3.9846
3.9497
3.7535
3.7494
3.7470
3.7161
3.7110
3.4989
3.4736
3.1742
3.1598
3.1556
3.1513
3.1366
2.5290
2.5134
2.5104
2.4942
2.4779
2.4751
2.4595
2.3942
2.3682
2.3424
2.2066
2.1893
2.1818
2.0700
2.0604
2.0572
2.0528
2.0496
2.0435
2.0400
2.0366
2.0304
2.0271
2.0229
2.0196
1.9227
1.8968
1.8921
1.8871
1.6751
1.6708
1.6612
1.6585
1.6550
1.6501
1.6451
1.6312
1.5742
-0.0060
OH
O
O
N
42
^{13}H NMR (400 MHz, $CDCl_3$)
1.02
1.00
1.04
2.09
2.09
1.05
1.04
1.03
1.04
2.08
1.06
1.08
1.12
1.13
1.07
1.13
9.0 8.5 8.0 7.5 7.0 6.5 6.0 5.5 5.0 4.5 4.0 3.5 3.0 2.5 2.0 1.5 1.0 0.5 0.0 -0.5 -1.0
f1 (ppm)

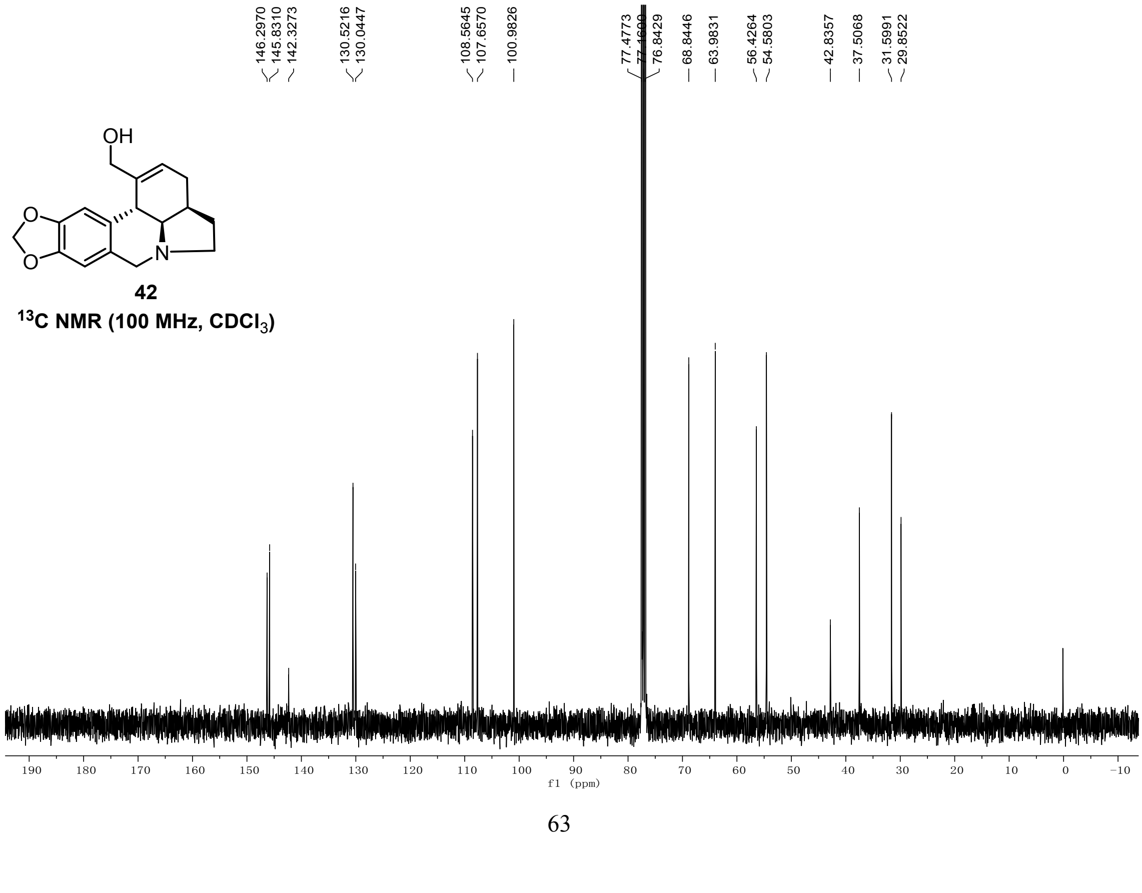
146.2970
145.8310
142.3273
130.5216
130.0447
108.5645
107.6570
100.9826
77.4773
76.8429
68.8446
63.9831
56.4264
54.5803
42.8357
37.5068
31.5991
29.8522
OH
O
O
N
42
^{13}C NMR (100 MHz, $CDCl_3$)
190 180 170 160 150 140 130 120 110 100 90 80 70 60 50 40 30 20 10 0 -10
f1 (ppm)

9. Determination of the Enantiomeric Excess by HPLC Analysis

Sample Info : AD 1 mL/min iprOH : hexane = 10 : 90 5 μL

Additional Info : Peak(s) manually integrated

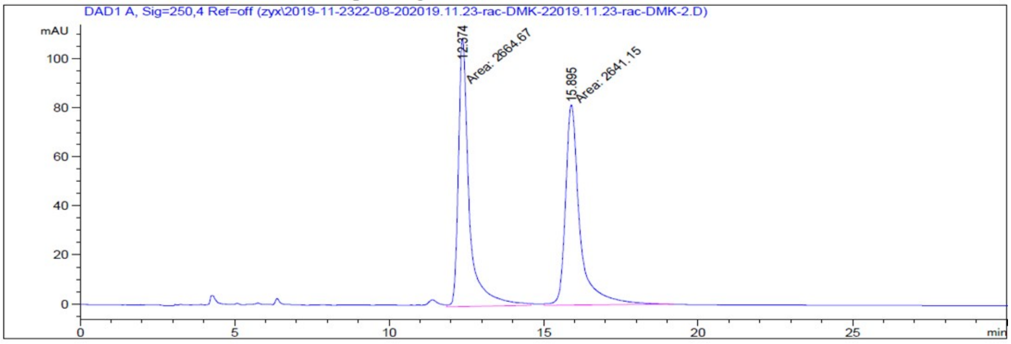

==

Area Percent Report

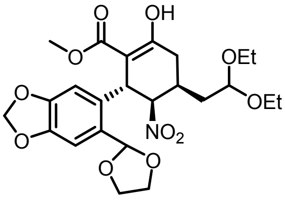

Rac - 12

Signal 1: DAD1 A, Sig=250,4 Ref=off

Peak #	RetTime [min]	Type	Width [min]	Area [mAU*s]	Height [mAU]	Area %
1	12.374	MM	0.4055	2664.66919	109.51096	50.2216
2	15.895	MM	0.5396	2641.15356	81.58147	49.7784
Totals :				5305.82275	191.09244	

==

Sample Info : AD 1 mL/min iprOH : hexane = 10 : 90 5 μL

Additional Info : Peak(s) manually integrated

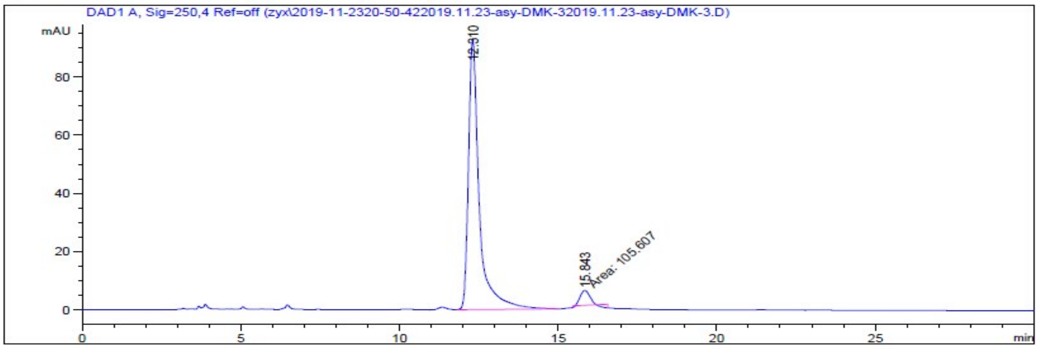

==

Area Percent Report

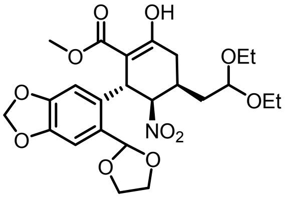

Asy - 12

Signal 1: DAD1 A, Sig=250,4 Ref=off

Peak #	RetTime [min]	Type	Width [min]	Area [mAU*s]	Height [mAU]	Area %
1	12.310	BB	0.3480	2202.74243	93.11471	95.4250
2	15.843	MM	0.3456	105.60723	5.09233	4.5750
Totals :				2308.34966	98.20704	

==

Sample Info : AD 1 mL/min iprOH : hexane = 20 : 80 8 μL

Additional Info : Peak(s) manually integrated

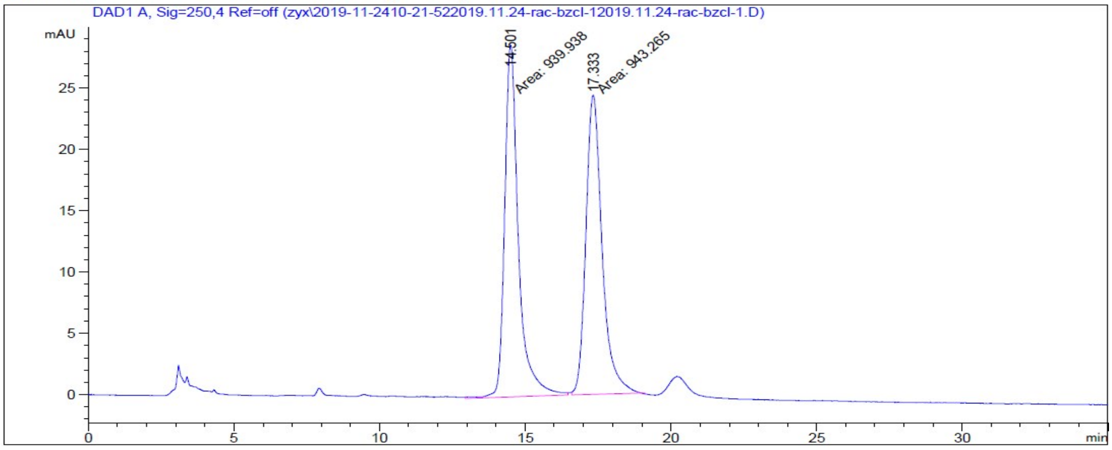

==

Area Percent Report

Signal 1: DAD1 A, Sig=250,4 Ref=off

Peak #	RetTime [min]	Type	Width [min]	Area [mAU*s]	Height [mAU]	Area %
1	14.501	MM	0.5463	939.93756	28.67789	49.9117
2	17.333	MM	0.6450	943.26508	24.37532	50.0883
Totals :				1883.20264	53.05322	

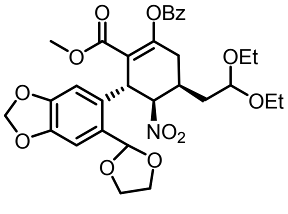

Rac - 11

==

Sample Info : AD 1 mL/min iprOH : hexane = 20 : 80 8 μL

Additional Info : Peak(s) manually integrated

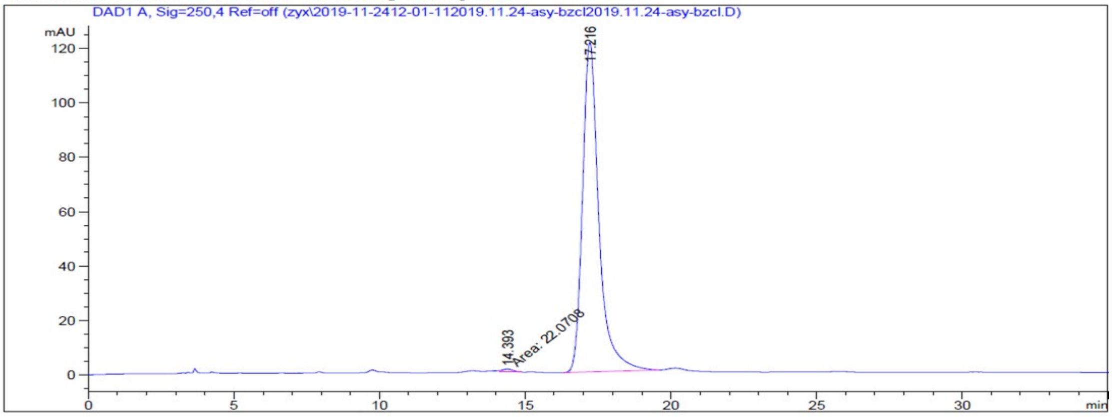

Area Percent Report

Signal 1: DAD1 A, Sig=250,4 Ref=off

Peak #	RetTime [min]	Type	Width [min]	Area [mAU*s]	Height [mAU]	Area %
1	14.393	MM	0.3881	22.07084	9.47901e-1	0.4643
2	17.216	BB	0.5834	4731.91211	121.21936	99.5357

Totals : 4753.98295 122.16726

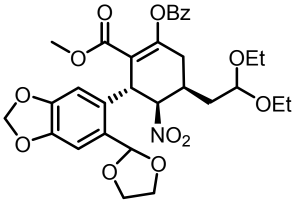

***Asy* - 11**

10. X-Ray Crystallographic Data for 11

Crystal data for **11**: $C_{31}H_{35}NO_{12}$, M = 613.60, a = 10.3885(2) Å, b = 14.7402(4) Å, c = 19.0069(5) Å, α = 90°, β = 90°, γ = 90°, V = 2910.50(12) Å^3, T = 100.(2) K, space group P212121, Z = 4, μ(Cu Kα) = 0.911 mm^{-1}, 28664 reflections measured, 5759 independent reflections (R_{int} = 0.0409). The final R_1 values were 0.0256 ($I > 2\sigma(I)$). The final $wR(F^2)$ values were 0.0631 ($I > 2\sigma(I)$). The final R_1 values were 0.0260 (all data). The final $wR(F^2)$ values were 0.0635 (all data). The goodness of fit on F^2 was 1.074. Flack parameter = 0.06(4).

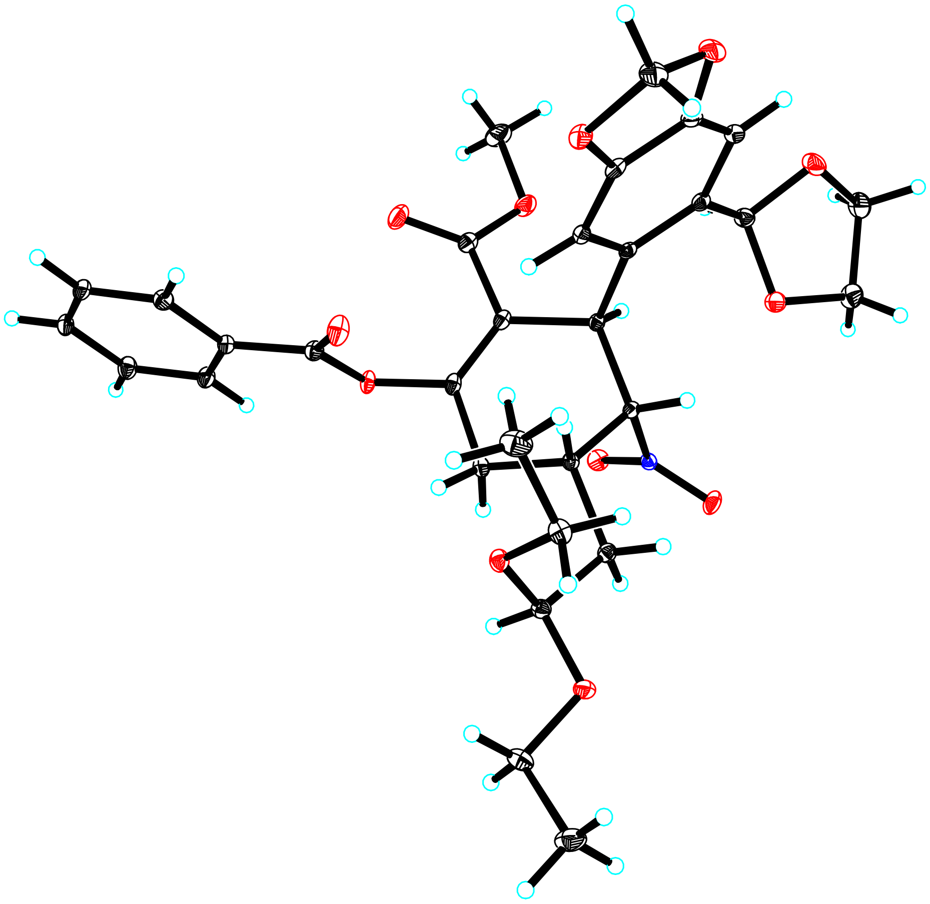

View of a molecule of **11** with the atom-labelling scheme.

Displacement ellipsoids are drawn at the 30% probability level.

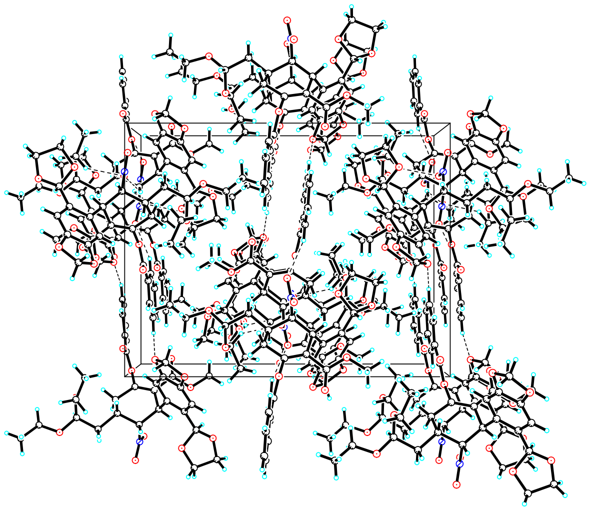

View of the pack drawing of **11**.

Hydrogen-bonds are shown as dashed lines.

Table 1. Crystal data and structure refinement for **11**.

Identification code	global	
Empirical formula	C31 H35 N O12	
Formula weight	613.60	
Temperature	100(2) K	
Wavelength	1.54178 Å	
Crystal system	Orthorhombic	
Space group	$P2_12_12_1$	
Unit cell dimensions	a = 10.3885(2) Å	α = 90°.
	b = 14.7402(4) Å	β = 90°.
	c = 19.0069(5) Å	γ = 90°.
Volume	2910.50(12) $Å^3$	
Z	4	
Density (calculated)	1.400 Mg/m^3	
Absorption coefficient	0.911 mm^{-1}	
F(000)	1296	
Crystal size	0.420 x 0.250 x 0.150 mm^3	
Theta range for data collection	4.85 to 72.37°.	
Index ranges	-12<=h<=12, -18<=k<=14, -21<=l<=23	
Reflections collected	28664	
Independent reflections	5759 [R(int) = 0.0409]	
Completeness to theta = 72.37°	99.8 %	
Absorption correction	Semi-empirical from equivalents	

Max. and min. transmission	0.88 and 0.70
Refinement method	Full-matrix least-squares on F^2
Data / restraints / parameters	5759 / 0 / 400
Goodness-of-fit on F^2	1.074
Final R indices [I>2sigma(I)]	R1 = 0.0256, wR2 = 0.0631
R indices (all data)	R1 = 0.0260, wR2 = 0.0635
Absolute structure parameter	0.06(4)
Largest diff. peak and hole	0.210 and -0.198 e.Å^{-3}
